# Rock-to-Metal Ratio: A Foundational Metric for Understanding Mine Wastes

**DOI:** 10.1021/acs.est.1c07875

**Published:** 2022-04-25

**Authors:** Nedal T. Nassar, Graham W. Lederer, Jamie L. Brainard, Abraham J. Padilla, Joseph D. Lessard

**Affiliations:** † National Minerals Information Center, U.S. Geological Survey, 12201 Sunrise Valley Drive, MS 988, Reston, Virginia 20192, United States; § Environment & Supply Chain Innovation, Apple Inc., One Apple Park Way, Cupertino, California 95014, United States; ⊥ Geology, Energy & Minerals Science Center, U.S. Geological Survey, 12201 Sunrise Valley Drive, Reston, Virginia, 20192, United States

**Keywords:** total material requirement, critical minerals, life cycle inventory, tailings, industrial ecology

## Abstract

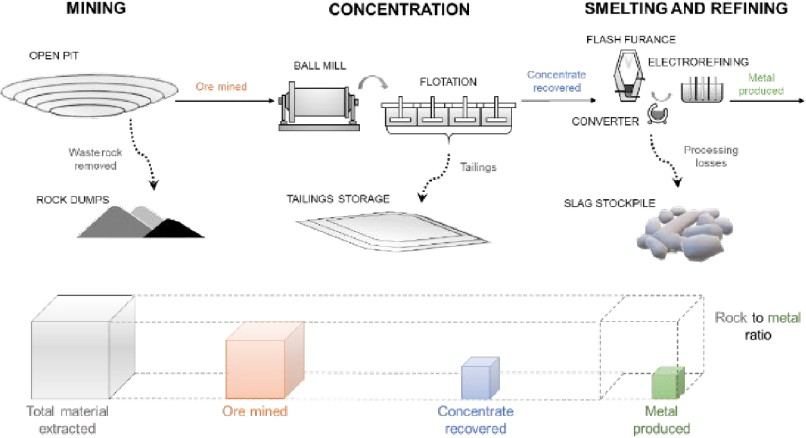

The quantity of ore mined and waste rock (i.e., overburden or barren rock) removed to produce a refined unit of a mineral commodity, its rock-to-metal ratio (RMR), is an important metric for understanding mine wastes and environmental burdens. In this analysis, we provide a comprehensive examination of RMRs for 25 commodities for 2018. The results indicate significant variability across commodities. Precious metals like gold have RMRs in the range of 10^5^–10^6^, while iron ore and aluminum are on the order of 10^1^. The results also indicate significant variability across operations for a single commodity. The interquartile range of RMRs for individual cobalt operations, for example, varies from 465 to 2157, with a global RMR of 859. RMR variability is mainly driven by ore grades and revenue contribution. The total attributable ore mined and waste rock removed in the production of these 25 commodities sums to 37.6 billion metric tons, 83% of which is attributable to iron ore, copper, and gold. RMRs provide an additional dimension for evaluating the impact of materials and material choice trade-offs. The results can enhance life cycle inventories and be extended to evaluate areas of surface disturbances, mine tailings, energy requirements, and associated greenhouse gas emissions.

## Abstract 2

Spanning 8 orders of magnitude across 25 mineral commodities and nearly 2000 operations, the quantity of ore mined and waste rock removed to produce a refined mineral commodity unit provides an additional dimension for evaluating the impact of materials and material choice trade-offs.

## Introduction

Mining of metallic and industrial minerals represents a volumetrically significant flow of material from the lithosphere to the anthroposphere. A direct relationship exists between the amount of material mined and the complexities of extracting, transporting, and transforming naturally occurring rocks into mineral commodities. The quantity of material mined, together with qualities such as ore grade, mineralogy, depth, and location, determines certain aspects of the environmental burdens associated with mineral commodity production. For example, as ore grades of mineral commodities decline, the amount of material that must be extracted to produce a certain amount of the commodity increases and its processing requires a greater amount of energy and other inputs.^1,2^ Importantly, large volumes of ore mined and associated waste removed (i.e., the overburden or barren rock removed to gain access to the ore) do not necessarily lead to large burdens across all environmental impact categories. A heavy mineral sands operation producing titanium may, for example, require the extraction of large amounts of ore and waste rock, but the associated environmental impacts are likely small because the tailings are relatively inert and usually are used to backfill and restore the open pit. In contrast, an underground nickel sulfide mine might extract relatively low volumes of ore and waste rock but may pose considerable environmental challenges due to the potential for acid mine drainage from the tailings.^3^ Nevertheless, quantifying the flows of ore and waste during mining and processing is increasingly necessary for understanding not only the potential environmental impacts but also the current and future supply of these mineral commodities, especially as demand for mineral commodities increases and ore grades decline.

Assessments of the total material requirement (TMR) aim to identify these impacts by explicitly quantifying the “hidden” mass flows associated with extractive operations.^4−6^ The value of these techniques is, however, limited by the availability of reliable and representative data. While some information on the quantities of ore mined and waste rock removed are reported periodically by the U.S. Geological Survey (USGS),^7^ these data are limited to operations in the United States. Additionally, due to concerns regarding the release of company proprietary information, the USGS data are aggregated so that it is impossible to determine the variability between operations. Previous studies on the TMR for mineral ores,^8,9^ although quite comprehensive in terms of the number of elements examined, are single point estimates based only on an assumed average ore grade (or as a function of average crustal abundance when ore grades are not available) and an assumed stripping ratio (i.e., the ratio of waste-to-ore mined) of 2 across all commodities. Similarly, commercially available life cycle inventories (LCI) (e.g., ecoinvent^10^) typically rely on one or a few representative operations or presumed global averages. While these may be reasonable assumptions when no other data are available the degree of variability between operations is not known or well-quantified.

For the mining and processing of mineral commodities, the quantity of rock required to be mined varies significantly by commodity and deposit, and depends on factors including ore grades, deposit type, mining method (e.g., open pit or underground), ore body depth, and stripping ratios even for a single mineral commodity. Having granular, operation-level information can thus help in not only understanding the variability in the data but can also assist companies in making informed decisions regarding, for example, where they source their raw materials. In this vein, there is a need for methodologically consistent and comprehensive data to enable accounting of companies’ raw material footprints in the same way companies assess their carbon footprints. A means to quantify TMR comprehensively would also add to the growing body of literature that considers environmental metrics needed to quantify material criticality, circularity, and sustainability. Using global warming potential (GWP) as a life cycle metric has helped to normalize the concept of carbon impacts;^11^ a similar but orthogonal metric for material use would help bring to the fore the impact of mined material consumption.

The main objective of this work thus is to develop a contemporary and globally representative estimate for the total amount of material that must be displaced to produce a given mineral commodity. Specifically, the goal is to determine the amount of waste rock, tailings, and processing losses generated during the production of a unit of finished metal: a rock-to-metal ratio (RMR). Additionally, this work aims to improve the understanding of the variability around this ratio and the underlying factors that control it. In this work, the RMR methodology was applied to 25 mineral commodities: aluminum (bauxite), chromium, cobalt, copper, gallium, gold, iridium, iron, lithium, magnesium (metal), molybdenum, nickel, palladium, platinum, rhodium, ruthenium, silicon, silver, tantalum, tin, titanium, tungsten, vanadium, zinc, and zirconium. Although these commodities were selected mainly on the basis of data availability, the diversity of their sources and uses provides a strong foundation for understanding the drivers and variability among the key parameters, which in turn provides essential information to understanding current and future supply potential and associated environmental impacts.

## METHODOLOGY

The RMR was calculated using the following equation:
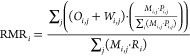
1where for mineral commodity *i* and mining operation *j*, *O* is the quantity of ore mined, *W* is the quantity of waste rock removed, *M* is the quantity of mineral commodity produced at the mine after beneficiation (i.e., mass of the commodity contained in a concentrate), *R* is the overall recovery rate during smelting and (or) refining (referred to as refinery recovery rate for simplicity), and *P* is the realized unit price of the commodity.

The RMR can be calculated for a single operation or aggregated to any desired level, including the global level, by summing across operations for a given year as illustrated in eq 1. The year 2018 was used as the base production year for calculating the RMR due to it being the most recent year with sufficiently complete data. However, there were several individual operations that did not operate at full capacity or for the entire duration of calendar year 2018 or began operations in 2018 but did not reach commercial production until the following year. In those cases, we used production data for the previous (2017) or following (2019) year, if available, to calculate a RMR that is more representative of the expected production level for that operation.

### System Boundaries

For each mineral commodity, a system boundary was defined. Each system included the mining and processing stages beginning with extraction of raw material at the mine site (run-of-mine) and ending with the production of the first or most common marketable refined end-product for each commodity (e.g., refined gold metal, direct reduced iron, or molybdic oxide, see Table S1). For some commodities, the system boundary included multiple refined end products (e.g., tantalum metal and oxide; tungsten metal and ammonium paratungstate). In all situations, the end product production quantity is reported in terms of commodity material-contained.

Certain commodities are commonly produced from multiple source materials, including nonrock sources such as brines. For cases in which there is no mined rock material involved in the production of a commodity, the RMR would not be applicable. To ensure consistency in the application of the methodology, our coverage excluded nonrock sources from the RMR calculation for those commodities. For example, lithium production from brine represents a different raw material source that, for the purposes of the RMR, is not comparable to the hard rock ores considered here. As such, only the portion of global lithium production from hard rock materials (e.g., spodumene, lepidolite, and petalite ores) are included in the RMR calculation. Similarly, magnesium metal is produced from brine and hard rock sources. Accordingly, the RMR was calculated only for magnesium metal production from evaporite minerals and carbonate rock. Metal production from ash and waste residues (e.g., vanadium from petroleum refining waste) was also excluded. Similarly, operations that reprocess mine tailings to recovery mineral commodities were excluded because the ore was mined in previous years. Technically, any reprocessed mine tailings should reduce the RMR calculation of the previous year. Most of these operations contribute little to global production and were thus excluded in this analysis.

### Data Sources and Global Coverage

Where possible, the parameters of the RMR eq (eq 1) were derived from reported information at the level of individual operations. The primary source for mine-level data was the SNL Metals and Mining (“SNL”) data set from S&P Global Market Intelligence.^12^ Relevant available data included annual ore production tonnages, mill-head grades, stripping ratios, and concentrator recovery rates. These data were either directly reported in corporate publications such as annual and quarterly company reports and presentations and U.S. Securities and Exchange Commission filings or their equivalent or derived by S&P Global Market Intelligence from information on similar operations.

For each individual entry, we also calculated its share of global production for the commodities that it produced. The primary source of total global commodity production data, including at the country-level, was the USGS.^13^ For some commodities, additional production data were available at the mine- or individual operation-level, which resulted in calculated total global commodity production quantities that are greater (by no more than 1%) than the USGS published estimates. In such instances, we calculated the share of global production based on the revised global production totals that better reflect current data availability.

Because of the corporate focus of the SNL data, global coverage varied notably by commodity with special emphasis on operational-level reporting for precious metals and the major base metals. In contrast, minor metals such as tungsten and tantalum had minimal reporting. For example, the sum of all copper production reported at the operational-level was within 6% of global copper production reported by the USGS, whereas no operational-level production data were reported for tantalum. The corporate focus of the SNL data was also biased toward commodity production from either large or publicly traded firms, resulting in a lack of data from state-owned enterprises and noncorporate artisanal operations.

Furthermore, the necessary data were not available for all operations of a given commodity. In such cases, we calculated the RMR using the best available data and, if necessary, adjusted the calculation to account for as large of a fraction of global production as possible to obtain a more representative RMR. For example, data for nickel were available for some but not all operations in Indonesia. An RMR for an “Indonesia-remainder” was calculated using the best available information on nickel deposits in Indonesia for the portion of that country’s nickel production that cannot be calculated for individual operations. Similar calculations were made for other countries and other commodities.

**Figure 1:**
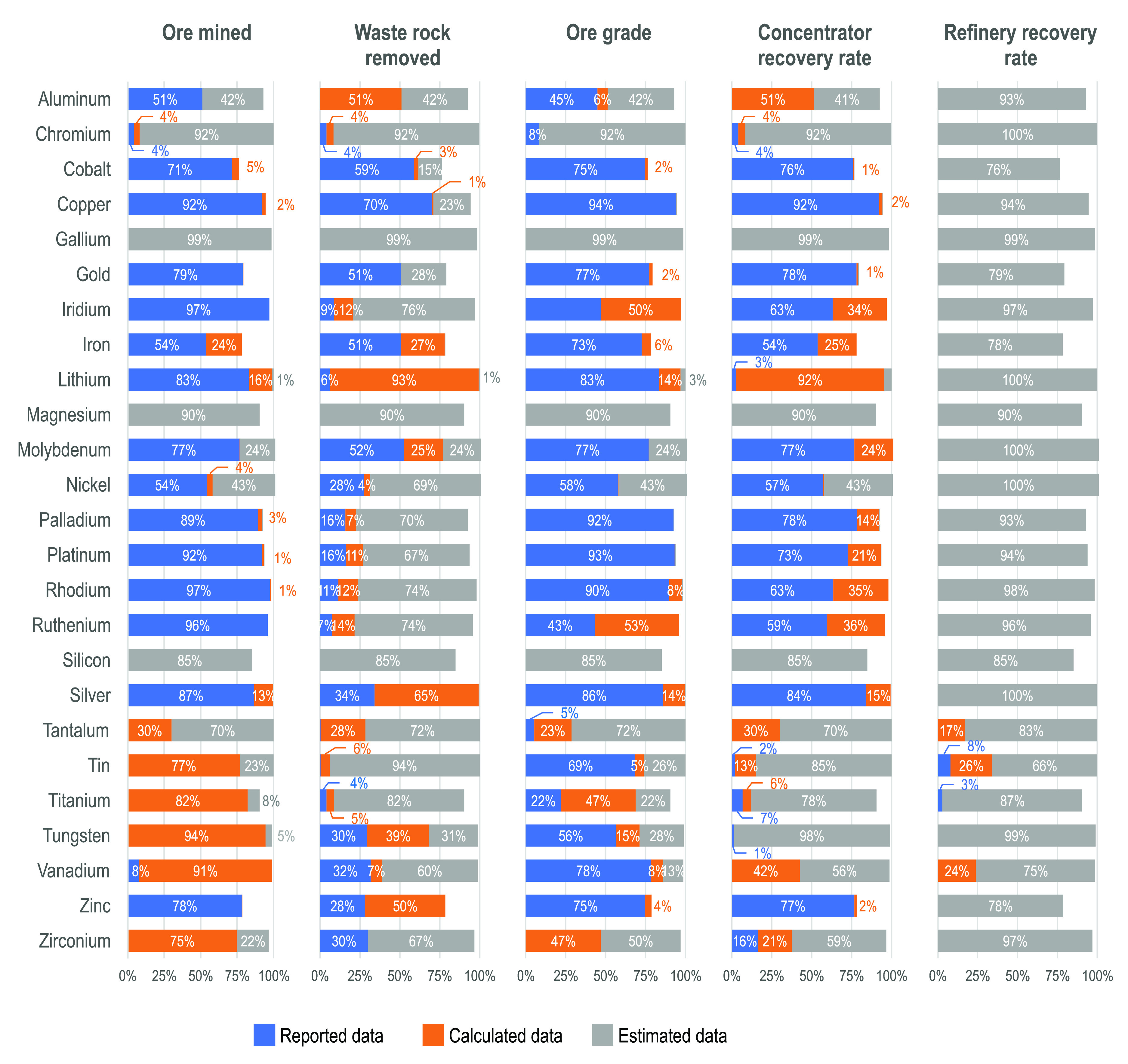
Data coverage for each commodity and variable, expressed as a percentage of world production. Individual values are reported at the facility level (blue), calculated from data reported at the facility level (orange), or estimated based on country totals and remainders (gray). For simplicity, the term “refinery recovery rate” is used to refer to the overall recovery rate of postmineral concentrate processes (e.g., smelters and refineries). Values less than 1% are not labeled.

Additionally, some operations had data for some but not all the parameters in the RMR calculation. The quantity of waste rock removed, for example, is not typically reported by operators. In such instances, these quantities were calculated based on concentrator recovery rates, ore grades, and waste-to-ore ratios. For example, if a mining operation’s concentrate production was reported, the quantity of ore that was milled can be calculated by dividing the concentrate production by the concentrator recovery rate and the mill-head grade. If the mill-head grade was not available, a reported ore grade for proven or probable reserves was used as an alternative. Although stockpiling is common practice, the quantity of ore milled was typically assumed to equal the quantity of ore mined (i.e., all material mined was also milled in the same year). This necessary assumption effectively means that the resultant RMR is specific to the ore that was milled that year rather than what ore was mined or waste rock was removed in that particular year. In general, most mines process the mined ores in the same year making this a minor systems boundary issue. Finally, the quantity of waste rock removed can be estimated from the quantity of ore mined if the waste-to-ore ratio was also available. This approach of starting from a mine’s concentrate production and back-calculating the quantity of ore mined and waste rock removed was the predominant method used. To aid in this process, a decision tree was developed to determine how RMR parameters should be estimated in the absence of directly reported quantities or the inability to calculate the quantities from the relevant factors (e.g., ore grades) (see Figure S1). The global coverage of reported, calculated, and estimated data for each RMR parameter is provided in Figure 1. Importantly, both reported and calculated quantities should be considered high-quality data in comparison to estimated data, which are more aggregate or generalized.

As illustrated in Figure 1, the quantity of ore mined, the ore grade, and the concentrator recovery rates were mostly reported or calculated based on reported data for major base and precious metals, but predominately estimated for the other commodities. This was especially the case for commodities with a small number of active operations such as gallium, tantalum, and vanadium. In such instances, we utilized production and related data from other publications, including various company reports and statements, Roskill commodity reports,^14−19^ industry reports, and geologic publications. Additional details regarding these special cases are provided in the following section.

The overall coverage should be interpreted as the global representativeness of the resultant RMR for that commodity, which is no less than 78% for any of the commodities analyzed. Importantly, this coverage refers only to the scope described in the section on System Boundaries and the Special Case Methods below. Moreover, it is specific to the end products noted for each mineral commodity in Table S1. For example, the RMR for magnesium includes only hard rock sources and thus excludes magnesium from brines. It is also specific to magnesium metal and excludes magnesium compounds. RMRs should thus only be interpreted as representative for the system boundaries and end products that are covered in the analysis.

### Special Case Methods

A small number of commodities required broader assumptions and (or) adjustments in our methodology in order to calculate a representative RMR due to either lack of granular data (e.g., artisanal operations for tantalum, tin, and tungsten), unconventional mining or processing methods (e.g., vanadium), extraction methods for which RMR is not applicable (e.g., lithium and magnesium metal from brines or vanadium from petroleum refining waste), and (or) decoupled mining and processing operations (e.g., silicon and gallium). Our methodology was adjusted as follows.

#### 3T Metals (Tantalum, Tin, Tungsten)

Unlike conventional large-scale mining operations, artisanal and small-scale mining (ASM) is typically conducted by individual miners on enriched placer deposits that can be easily exploited with limited mechanized equipment. ASM operations contribute a sizable portion of the annual tantalum, tin, and tungsten global supply. However, the lack of reliable data on the production and mined material grades and tonnages makes direct quantification of the RMR for artisanal operations very difficult. Thus, we calculate the RMR at the country-level and assume that, because of the nature of ASM operations, only ore is extracted and therefore no additional waste is associated with the mining operation (i.e., there is minimal removal of overburden soil and rock, and the majority of total material extracted is the gangue rock that is removed during the concentration of the ore, which is captured in our calculations by a concentrator recovery rate). In the absence of reported ore grades, we assumed average grades of 0.5%W, 1% Sn, or 0.164% Ta based on published studies of artisanal operations exploiting placer and alluvial deposits in Africa.^20,21^

#### Silicon

Leading global silicon metal producers are vertically integrated multinational operations that typically process raw material from multiple sources at multiple locations resulting in a data gap between raw material (quartzite) production and silicon metal extraction. Because of the lack of data availability at more granular levels than the multinational company level, we calculate the RMR at the global level for production of silicon metal from high-purity (metallurgical grade) quartzite feedstock based on the ecoinvent LCI recovery profile,^22^ and exclude ferrosilicon as well as other forms of silicon for industrial uses (e.g., silicon carbide) in our calculation. While our single-point silicon estimate does not offer the same granularity in data as the other commodities in our study, we believe it serves as a useful comparison against previous studies that use single-point estimates for all commodities studied.

#### Vanadium

Approximately 80% of global primary vanadium is derived as a coproduct from vanadiferous titanomagnetite ores, recovered from vanadium-rich steel slags (“vanadium slags”) produced during steelmaking. Although steelmaking operations are typically decoupled from the mining/ore source, to calculate a representative RMR at the steelmaking plant-level where vanadium recovery takes place, we treat mining and steelmaking as joint operations with the vanadium slag representing the “concentrate” stage of a conventional mining and beneficiation operation.

#### Gallium

Gallium is extracted as a byproduct during the processing of bauxite and zinc ores, and as a result gallium recovery at the smelter is typically decoupled from the mined ore source. Furthermore, gallium producers typically depend on imported feedstock for gallium extraction. Thus, we calculated the RMR at the country-level only for countries that recover gallium, using trade data to determine the origin of the ores, and calculate a “gallium ore” composition as a proportionally weighted blend of domestic and imported ore based on the country of origin.

#### Brines

As previously noted, nonhard rock sources such as brines are excluded from the RMR calculation. This has implications for lithium and magnesium metal. In recent years, Australia’s production of lithium from hard rock sources, namely spodumene, has increased markedly. As such, brine production accounted for roughly 30% of total lithium production in 2018 but was as high as 55% only a few years prior in 2015.^13^ Similarly, magnesium metal is mainly produced from hard-rock sources (e.g., dolomite and carnallite), with magnesium metal from brines being sourced only from Israel and the United States, which are estimated to have accounted for less than 10% of global primary magnesium production in 2018.^15^ Nevertheless, RMR results for both lithium and magnesium metal should only be interpreted as being representative of hard-rock sources.

### Refinery Recovery Rate

To obtain the “ultimate” amount of metal produced, a refinery recovery rate was utilized. Including operation-level refinery recovery rates, while theoretically possible, is quite complex given that mineral concentrates are shipped globally to different smelters and refineries and one would need to trace the flows of the commodities from the mines to the appropriate smelter and (or) refinery. For some commodities, this is not possible as the trade data are not sufficiently detailed. It is also unnecessary because the recovery rates of these downstream operations are relatively high (often 90% or more^23−31^) and vary minimally across operations. An overall global *R* average of 90% was thus used for each mineral commodity, except when specific information was available. Details are provided in the Supporting Information (Table S2).

### Parameter Data Description

**Figure 2:**
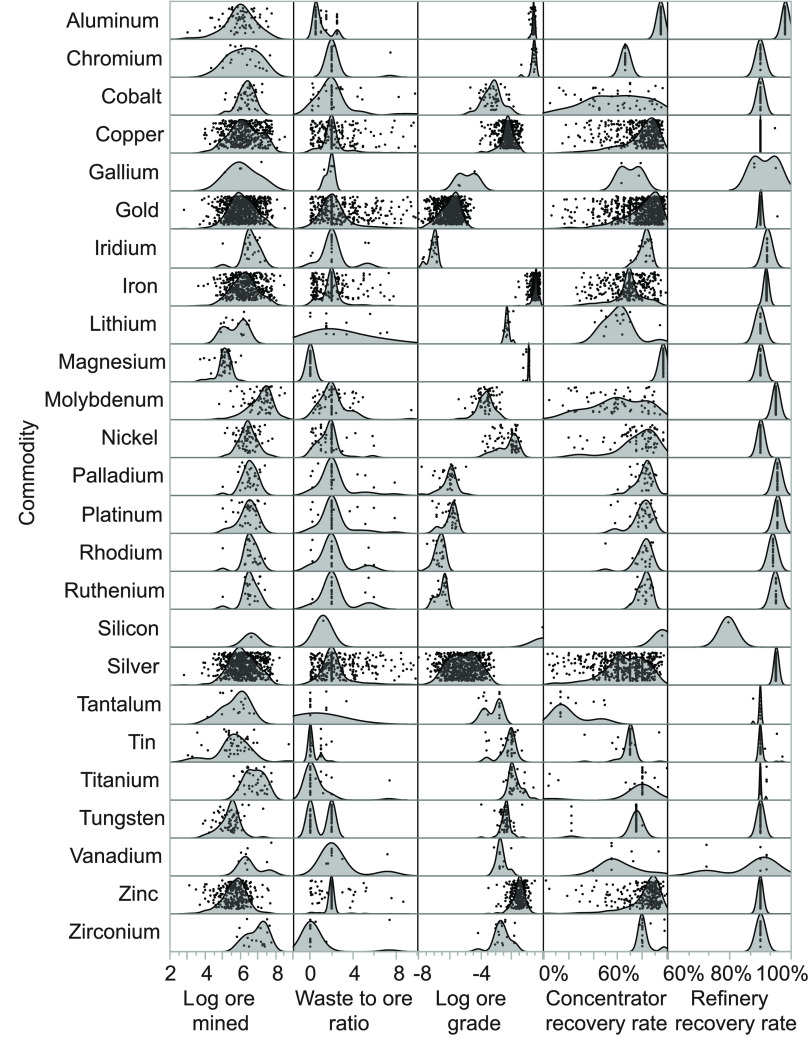
For each mineral commodity examined, the figure displays the (1) quantity of ore mined (log_10_ scale), (2) waste-to-ore ratio, (3), ore grade (log_10_ scale), (4) concentrator recovery rate, and (5) refinery recovery rate. Each individual operation or country-remainder is represented by a single point and the overall distribution is displayed by the gray area.

Figure 2 provides the distributions of the data utilized in the analysis for each RMR parameter by mineral commodity. As illustrated in Figure 2, the parameter data are distributed in a narrow range for some commodities (e.g., ore grades for aluminum, chromium, and magnesium) but not others (e.g., ore grades for silver). Across parameters, the data are much more narrowly distributed for waste-to-ore ratios, concentrator recovery rates, and refinery recovery rates than for ore grades and the quantities of ore mined, both of which are displayed on log_10_ scales. From Figure 2, one can also see that the number of operations varies notably by commodity. Data for many operations were available for some commodities such as copper, gold, iron and silver, but only a small number of operations had data for minor or specialty commodities. This reflects both the number of mines currently operating and data availability. The specific number of operations analyzed and the percent of global production covered for each commodity are provided in the Supporting Information (Table S1).

Note that Figure 2 displays reported, calculated, and estimated data. The estimated data that were assumed constant across many operations (e.g., refinery recovery rates) are visually identifiable as the distributions with single values. Similarly, the bimodal distribution of waste-to-ore ratios for some commodities (e.g., tungsten) reflects the assumption that a factor of 2 is utilized for surface mine operations and a factor of 0 is utilized for underground and artisanal operations when no specific information was available.

### Allocation of Impacts

Given that most operations produce more than a single commodity, the burdens (i.e., the quantity of ore mined and waste rock removed) were allocated to an individual commodity based on its revenue share, which was estimated as the product of the mine production and realized unit price relative to the revenue from all commodities. Unit prices were obtained from the USGS.^32^ A uniform unit price was used for each commodity except for certain lithium, tantalum, and vanadium operations for which uniform prices would not provide an accurate representation of revenue shares and for which commodity-specific revenues were reported by the companies. This was necessary for operations that produce multiple grades of concentrate (e.g., Bald Hill and Pilgangoora 1 in Australia). Details are provided in the Supporting Information (Table S3).

This economic allocation is one of the most widely recommended baseline methods in most life-cycle assessment (LCA) allocation situations and its appropriateness stems from the rationale that economic value drives actions.^33^ Using economic allocation thus allows for the appropriate allocation of burdens for coproduct or byproduct metals, which provide a moderate to limited revenue contribution to most mining operations.^34^ Additionally, mineral commodities that occur but are not recovered receive no burden allocation. Again, this is important for many byproducts (e.g., gallium) that may be extracted with the ores but not always recovered.^35^ If these byproducts do become economic to recover at a later date, then the RMR may need to be adjusted to account for the new revenue streams that are generated from their recovery. This would be similar to the reprocessing of mine tailings, with additional allocations needed to account for any mineral commodities that were previously not recovered. A methodological question remains as to when to allocate the burdens: the year the ore was mined or the year the tailings were retreated. As previously noted, tailings retreatments were excluded from this analysis, effectively suggesting that the allocations belong to the year the ore was mined. Other analysts may elect to allocate the burdens differently.

## Results and Discussion

**Figure 3:**
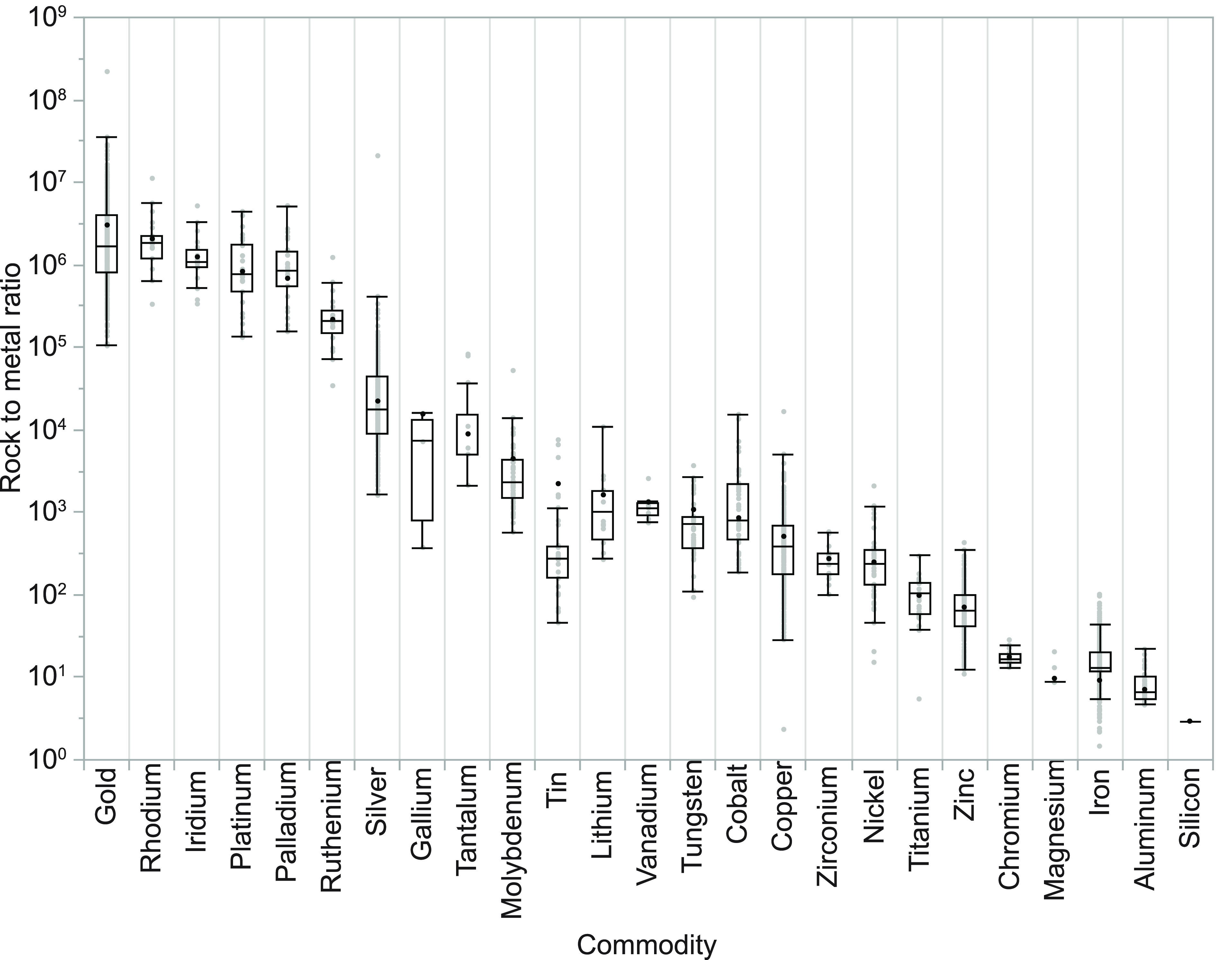
Box and whisker plot of RMRs (log_10_ scale on vertical axis) for each mineral commodity. The box is defined by the 1st and 3rd quartile, with the median shown as a horizontal line; whiskers extend 1.5 times the interquartile range beyond the box. RMRs for individual operations are shown in gray circles. Mineral commodities are ordered by global RMR values, which are shown as black circles.

RMRs were calculated for each commodity both for individual operations as well as the global level (eq 1). Global RMRs across all commodities ranged by almost exactly 6 orders of magnitude from 3 for Si to 3 × 10^6^ for gold (Table S4 and Figure S2), with RMRs for individual operations ranging by 8 orders of magnitude, from 1.5 to 2.2 × 10^8^ (*n* = 1928 individual operations or country-estimates) (Figure 3 and Table S5). Precious metals, led by gold, make up the upper end of this range with individual RMRs ranging between 1.6 × 10^3^ and 2.2 × 10^8^, whereas the ferrous and nonferrous metals generally plot at the lower end, with RMRs ranging between 1.5 and 5.2 × 10^4^. While iron has one of the lowest global RMR values of the commodities analyzed in this analysis (∼10^1^), it has the largest attributable total quantity of ore mined and waste rock removed at approximately 12.9 billion metric tons after adjusting for global coverage (Figure S3 and Table S6). This is due to large quantities of iron ore mined (over 1 billion metric tons per year). In contrast, the global mine production of gold is quite small (∼3 thousand metric tons). However, because gold has the highest global RMR, it also has a very high attributable total amount of ore mined and waste rock removed at approximately 9.1 billion metric tons (after adjusting for coverage), third only to iron ore and copper (9.4 billion metric tons). Indeed, the total attributable quantity of ore mined and waste rock removed, after adjustments for coverage, for these three commodities represents 83.4% of all the attributable ore mined and waste rock removed from the entire set of 25 mineral commodities examined (37.6 billion metric tons, Table S6).

**Figure 4:**
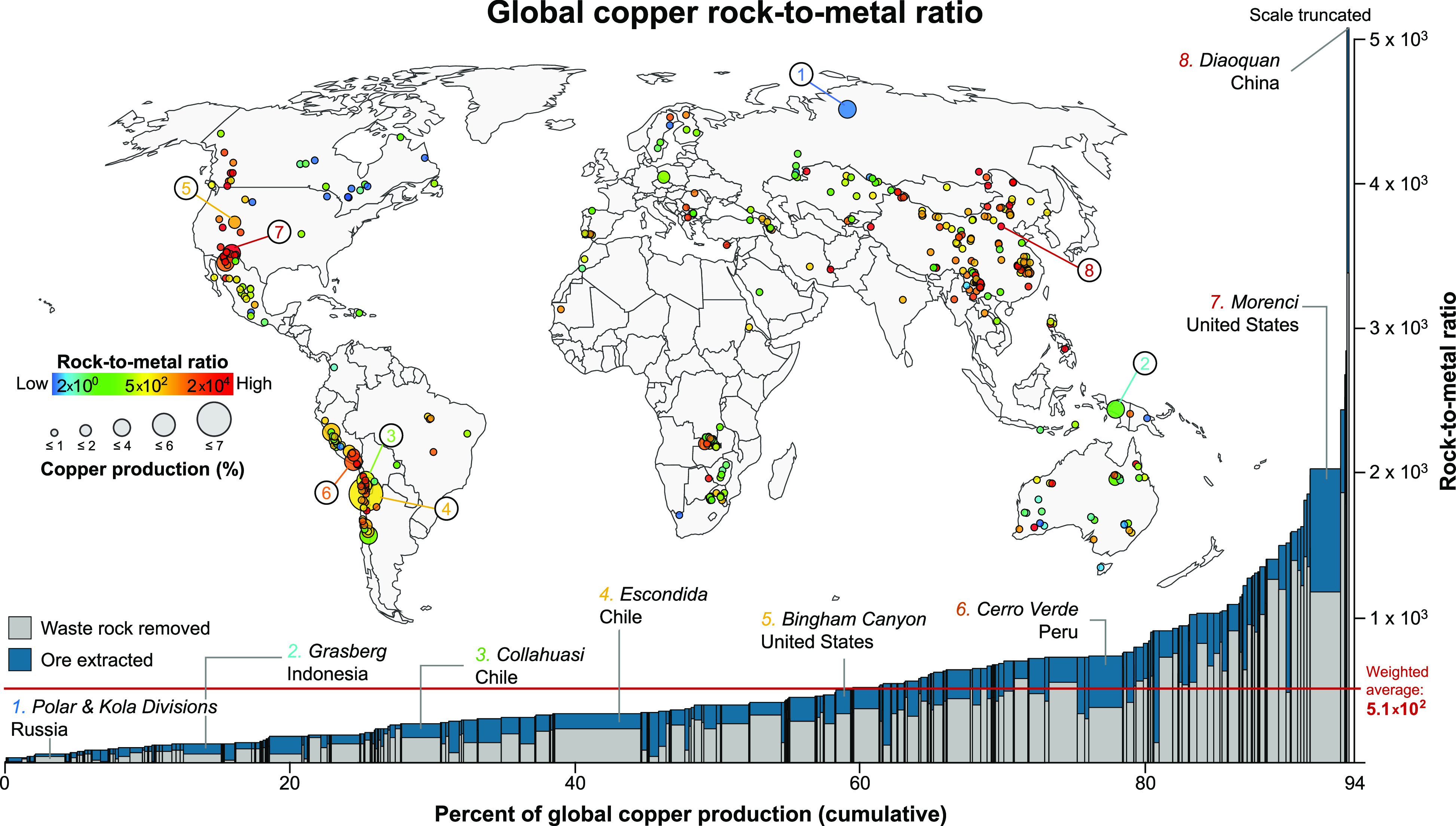
Map of the global distribution of copper operations and bar plot of cumulative share of total global copper production. Each individual operation is plotted as a single circle on the map and single bar on the plot. The colors of the circles indicate individual RMRs, which range from a low of 2.3 to a high of 1.7 × 10^4^ and yield a global RMR of 5.1 × 10^2^ (*n* = 431). The sizes of the circles are proportional to an operation’s share (in percent) of total global copper production, which range from a low of <0.001% to a high of 6.1% for a total global coverage of 94% of 2018 global copper production reported by the U.S. Geological Survey.^32^ Operations are ordered from lowest to highest RMR on the bar plot.

We also find that RMRs at the individual operation level vary widely, in some cases across several orders of magnitude, within a single commodity (Figure 3 and Table S5). For example, RMRs for copper range from 2.3 to 1.7 × 10^4^, with 90% of global copper production having RMRs of 1.5 × 10^3^ or less (see Figure 4 for copper and Figures S4–S27 and Tables S5 and S7 for all commodities analyzed). While several factors contribute to the wide range, the results suggest that operation size, as measured by their share of total global production, and the geographic location are not significant determining factors. For example, for the 431 copper operations included in our analysis, small producers (e.g., Diaoquan in China, and Minera Valle Central in Chile) and large producers (e.g., Bingham Canyon in the United States, Cerro Verde in Peru, and Collahuasi in Chile) occur across the entire RMR spectrum (Figure 4). We also find that, whereas the large copper producers stand out (e.g., Escondida in Chile), smaller producers together also account for significant portions of production and influence the overall global RMR (e.g., operations with RMR > 1.0 × 10^3^). In contrast, factors such as the revenue allocation for individual operations that produce more than one commodity can affect the RMR in that the burden of wastes associated with an individual operation can be proportionally distributed across the commodities resulting in lower RMRs for all commodities (e.g., Nornickel’s Kola and Polar Divisions in Russia, which primarily derive their revenues from nickel;^36^ see Figure 4).

### Factor Analysis

**Figure 5:**
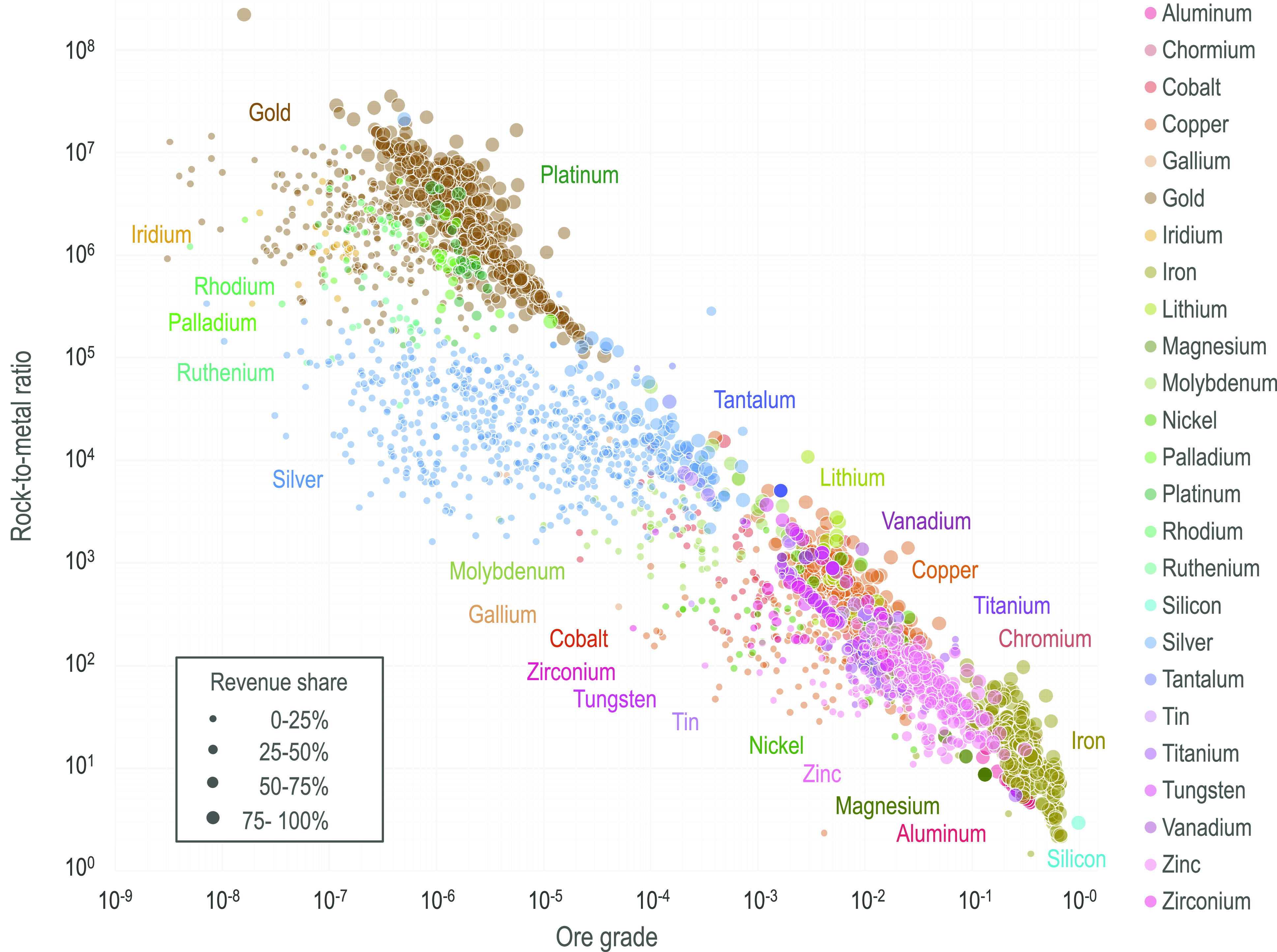
Rock-to-metal ratio (vertical axis) versus ore grade (horizontal axis) by mineral commodity by individual operation. Axes are on a log_10_–log_10_ scale. Colors correspond to different commodities. Marker size corresponds to revenue share (economic allocation) attributable to the mineral commodity at the specific operation.

The magnitude and variability of RMRs depend on the input parameters used in the calculation, namely ore grade, waste-to-ore ratio, concentrator recovery rate, refinery recovery rate, and economic allocation (revenue share). Much of the variability between commodities can be explained by the differences in ore grade, which in turn relate to differences in crustal abundance. For example, aluminum has a global RMR of 7.1, is mined from bauxite with an average ore grade of 25.6% Al, and constitutes 11.4% of continental crust.^37^ By contrast, platinum has a global RMR of 8.3 × 10^5^, an average ore grade of 1.4 ppm, and a crustal abundance of 0.5 ppb.^37^ Ore grade also exerts the primary control on variability in the RMR between mining operations producing the same commodity. The distribution of ore grades for an individual commodity reflects the different deposit types from which it is mined. For example, hard rock titanium deposits typically have higher grades than heavy mineral beach placers and therefore a lower RMR. Ore grades for different deposit types may form distinct populations or a continuous distribution spanning several orders of magnitude. As previously noted, RMRs for copper range from 2.3 to 1.7 × 10^4^ and correspond to ore grades ranging from 5% to 0.01% Cu. The relationship between ore grade and RMR is illustrated in Figure 5, with the same data plotted for subsets of the commodities per graphic provided in Figures S28–S32 for visual clarity.

Another important variable that affects the RMR is revenue share. For example, silver is often recovered as a byproduct of lead–zinc, gold, or copper; because the waste and losses associated with mining these metals are distributed over several commodities, silver has a lower RMR than if no other commodities were recovered from the same operations. The effect of byproduct recovery is that an operation can produce a commodity with the same RMR at a much lower ore grade in comparison to a facility mining the same commodity as a primary product. In Figure 5 the effect of byproduct recovery is shown graphically by points with decreasing revenue share (marker size) shifted left toward lower grades at the same RMR.

Mining method (surface or underground) and material type (hard rock or unconsolidated sediment) are also important factors that determine RMRs because of the different amount of waste material that must be removed. For example, even with the same ore grade, an underground mine with minimal waste rock removal would have a lower RMR than an open pit mine with an average stripping ratio of 2 tons of waste removed for every ton of ore. Similarly, unconsolidated sediment, such as alluvial tin and placer gold deposits, can be mined with no overburden stripping or waste removal, compared to hard rock open pit mines with similar grades.

To confirm these findings, predictor screening tests^38^ using a bootstrap forest model with 100 decision trees were used to determine which factors exert the strongest effect on the RMR. The contribution of each factor to the RMR is presented as a percentage in Table S8 and Figure S33. Overall, ore grade is the most influential variable controlling the RMR (at an overall contribution of 68.9%), followed by revenue share (16.9%), waste to rock ratio (5.4%), refinery recovery rate (4.9%), and concentrator recovery rate (4.0%). Notably, the RMR for each commodity may be strongly controlled by one or more variables. For example, bauxite ore grades occupy a relatively narrow range, therefore waste to ore ratio explains the variability in the RMR, whereas revenue share is the primary factor determining the RMR for silver.

Given the strong dependency on ore grades, the results of this work are generally comparable (i.e., similar order of magnitude) to previous studies on ore-TMR^8,9^ that were based solely on an “average” ore grade (or crustal abundance) and a constant strip ratio of 2. The RMRs presented do, however, indicate considerable variation for the RMR within commodities that was previously not described. Indeed, because these results show that the variability in the RMR for an individual commodity can span several orders of magnitude, one should rely on “average” ore grades and other generalized parameters only when more specific data are not available.

Importantly, ore grades and the amount of waste rock removed (and, in turn, the waste-to-ore and the rock-to-metal ratios) can vary notably throughout the life of the mine. Because this analysis is a snapshot of a single year it includes a mix of mines at various stages of their life. As such, biases due to factors that are age-of-mine dependent are assumed to be minimal, especially for mineral commodities with a large number of mines included in the assessment.

### Relating RMR to Crustal Abundance

The correlations between ore grade, production, reserves, price, and crustal abundance have been the focus of much research. McKelvey^39,40^ recognized that the amount of reserves of metals reflects their abundance in Earth’s crust. Skinner^41^ expanded this analysis to illustrate the relationship between crustal abundance and mine production, as well as the relationship between ore grade and energy.

**Figure 6:**
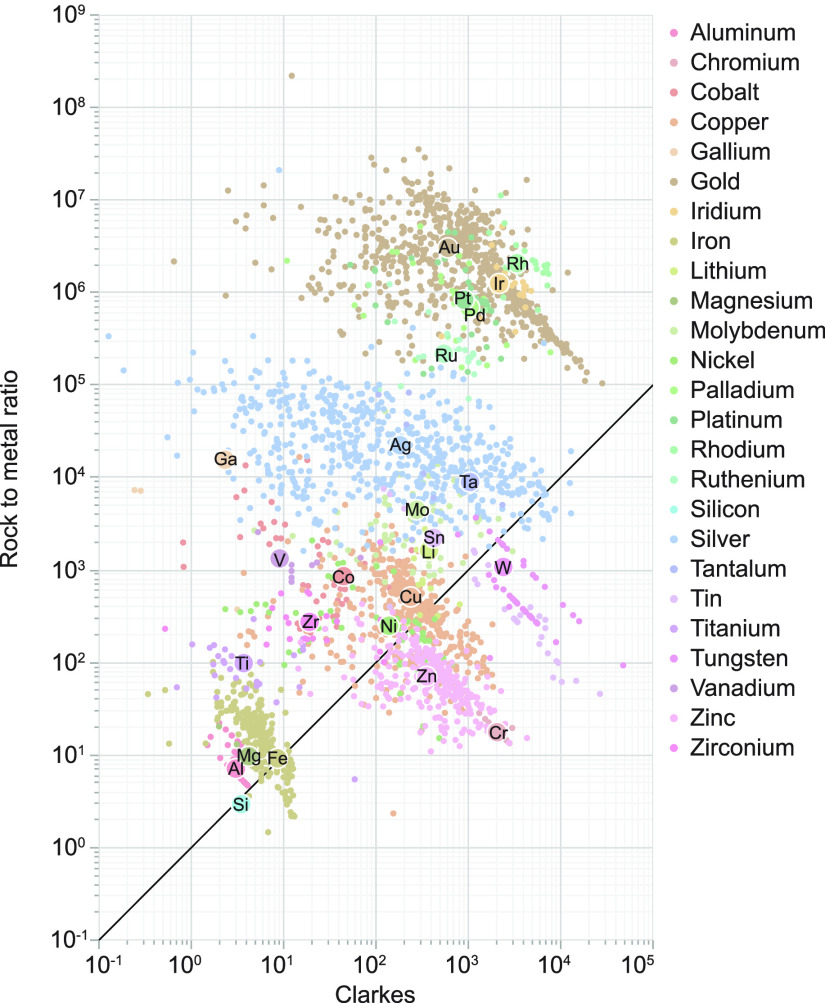
Scatter plot of RMRs vs enrichment factor relative to continental crust (Clarkes). The horizonal axis represents the work nature has done to concentrate an element, whereas the vertical axis represents the concentration required by society to recover an element. Vertical and horizontal axes are plot on log_10_ scales. Large circles represent global RMR averages; small circles represent individual operations.

RMRs are correlated with average continental crustal abundance values.^37^ This correlation reflects the dependence of RMRs on ore grade (Figure 5), which in turn depend on crustal abundance. Ore grades result from the primordial abundance of an element on Earth as well as the geological processes that have differentiated the crust and concentrated elements into mineral resources. A convenient unit of measurement for enrichment factors is the Clarke, a dimensionless number given by the formula:
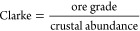
2Using eq 2, average enrichment factors can be calculated for each commodity (Figure 6). Abundant elements, such as Fe, Al, Si, and Mg, are minable at 1 to 10 Clarkes, whereas scarcer elements such as Pt, Au, and Ta require enrichment of 100 to 1000 Clarkes.

The Clarke number of ores indicates the degree of enrichment by geological processes. By analogy, the RMR represents the anthropogenic enrichment required to convert ore to metallic mineral commodities. By plotting RMRs against the Clarke numbers of ores (Figure 6), several observations become apparent. In general, there is a positive relationship between RMR and Clarkes. Major elements, such as Fe, Al, and Mg, are abundant and require much less anthropogenic enrichment, whereas minor and trace elements require enrichment by both geological and anthropogenic processes. Some elements show exceptions to this general trend: Ga, for example, is mined from deposits that are not particularly enriched relative to crustal background and therefore require much industrial processing to produce Ga in a usable form. Cr, on the other hand, is concentrated so effectively by natural processes that relatively little additional industrial processing is required.

### Applications, Limitations, and Future Work

The RMR provides a consistent, versatile framework that can facilitate the comparison of mined materials across commodities and between operations. The system boundaries are scalable and adaptable to focus on specific commodities, commodity forms, countries, or mining operations of interest. Individually, the parameters of the RMR, such as ore grade and waste-to-ore ratio, can also provide useful information and be scaled to provide insights regarding regional and global averages and variability.

The utility of the RMR can be extended to calculate other factors such as areas of surface disturbances, total mass of solid waste generated, energy requirements, and associated greenhouse gas emissions. For example, the information generated in calculating the RMR could be used to better understand the volumes and contents of waste going to tailings, a topic of increased international interest given recent tailing dam failures.^42^ The RMR can also provide an additional dimension when evaluating the impact of materials or material choice trade-offs. For example, material substitution is often proposed as a strategy to mitigate material criticality^43,44^ and to promote sustainability;^45,46^ applying the RMR to the materials in question aligns them to a common unit of “rock mined” thereby enabling a fairer comparison of materials alongside other environmental assessments, like their carbon footprints. Furthermore, understanding the RMR and its variability can more completely quantify the benefits of recycling as it pertains to offsetting the need for new materials (e.g., the end-of-life recycling of 1 kg of gold offsets an average of 3000 t of ore and waste that did not need to be mined or removed). Contextualizing mined material use in terms of the rock mined also helps educate the general public and policymakers about the material intensity and the scale of activity involved in supplying the materials and products required for everyday life and the entire economy (e.g., ref (47)).

A manufacturing company that utilizes these mineral commodities in their products or processes may use the RMR to inform purchasing decisions. It is important to remember, however, that the RMR is only one component of the environmental burdens associated with a mining operation. Although average RMRs (or TMRs) may be correlated with the energy needed for transportation and comminution of mined material, and therefore generally correlated to the associated greenhouse gas (GHG) emissions,^48^ there are other factors that need to be taken into consideration. For example, underground mines typically have lower RMRs but also require energy for ventilation and temperature control that surface mines do not. Similarly, the proximity to and type of transportation and energy used by a mining operation may increase or decrease GHG emissions.

Furthermore, the lowest RMR may not necessarily be correlated to lower overall environmental burdens. As noted in the Introduction, the RMR is not an indicator of other potentially harmful impacts such as acid mine drainage from sulfide minerals or chemical and sediment inputs to waterways. Interestingly, minimizing the RMR may favor mining the highest-grade deposits and potentially shorten the life-of-mine for some operations. Additionally, some low RMRs for specific metals may simply be the result of allocation of burdens among commodities that are coproduced by a single operation. The RMR should thus not be interpreted as an environmental indicator nor should it be assumed to be proportional to environmental impacts or replace a full cradle-to-gate LCA that accounts for all material and energy inputs and emissions to air, water, and land at each life cycle stage. Instead, the underlying RMR data can be incorporated to enhance LCIs, which as noted in the Introduction are typically based on generalized single-point estimates of ore grades and waste-to-ore ratios.

The RMR is also not equivalent to the crustal scarcity indicator,^49^ the surplus ore method,^50^ or other similar methods^51^ that assume a cumulative relationship between grade and tonnage extracted. Resource depletion indicators require assumptions about the likelihood of future mineral resource discoveries, the quantities of undiscovered in situ resources, and the development of extraction technology. In contrast, the RMR calculation presented here represents as closely as possible the actual quantities of materials extracted. The RMR should therefore be thought of as a neutral indicator that on its own does not imply any positive or negative consequences, nor require immediate interventions or policy changes. Because RMRs provided here represent a snapshot in time, it will be important to review, update, and enhance the underlying data as changes in ore grades (generally a decline through time), prices (volatile and cyclical), and other factors are expected. Nevertheless, major changes in the RMR parameters across all operations of any mineral commodity are unlikely in the short term and these results are believed to be representative of the contemporary situation.

## Supporting Information Available

Decision tree for data selection and calculations (Figure S1); periodic tables of RMRs and total attributable material extracted (Figures S2 and S3); maps of the global distributions RMR by operation and commodity (Figures S4–S27); RMR versus ore grade log–log scatterplots (Figures S28–S32); contribution of each factor to the overall RMR (Figure S33); number of operations, percent of global coverage, and descriptions of end products for each mineral commodity analyzed (Table S1); refinery rates utilized in the analysis for certain commodities (Table S2); operations for which economic allocation was based on specific revenue share information (Table S3); summary of results for the rock-to-metal ratio by mineral commodity (Table S4); summary statistics for the RMR by mineral commodity (Table S5); total material extracted, waste rock remove, and ore mined by mineral commodity after adjusting for global coverage (Table S6); percentage of global production with RMR values equal to or less than noted levels (Table S7); proportion of contribution of each factor to the overall RMR (Table S8) (PDF)

## ABBREVIATIONS

ASM: Artisanal and small-scale mining
GHG: Greenhouse gas emissions
LCA: Life cycle assessment
LCI: Life cycle inventory
RMR: Rock-to-metal ratio
TMR: Total Material Requirement
