# Supplementary material for: Rock-to-Metal Ratio: A Foundational Metric for Understanding Mine Wastes
Source: Environ Sci Technol. 2022 Apr 25;56(10):6710–21. doi: 10.1021/acs.est.1c07875 (PMC9118561; doi:10.1021/acs.est.1c07875)
Supplement: Supplementary file 1 — es1c07875_si_001.pdf [file es1c07875_si_001.pdf]

SUPPORTING INFORMATION
for
Rock-to-metal ratio: a foundational metric for understanding mine wastes

Nedal T. Nassar[1†]; Graham W. Lederer[2]; Jamie L. Brainard[1]; Abraham J. Padilla[1]; Joseph D. Lessard[3]

[1]U.S. Geological Survey, National Minerals Information Center
[2]U.S. Geological Survey, Geology, Energy & Minerals Science Center
[3]Environment & Supply Chain Innovation, Apple Inc.
†Correspondences: nnassar@usgs.gov

Contents

Methodological details 2
Description of RMR results 5
Factor analysis 40
References 42

List of Tables

Table S1. Number of operations, percent of global coverage, and descriptions of end products for each mineral commodity analyzed 3
Table S2. Refinery recovery rates utilized in this analysis, for commodities for which more representative data were available 4
Table S3. Operations for which the economic allocation was based on specific revenue shares information 4
Table S4. Summary of results for each component of the rock-to-metal ratio by mineral commodity. 5
Table S5. Summary statistics for the RMR by mineral commodity 6
Table S6. Total material extracted, waste rock removed, and ore mined by mineral commodity after adjusting for global coverage 7
Table S7. Percentages of global production with RMR values equal to or less than noted levels 9
Table S8. Contribution of each factor to the overall RMR expressed as a percentage. 40

List of Figures

Figure S1. Decision tree for data selection and calculations. 2
Figure S2. Periodic table displaying the 2018 global RMR for the mineral commodities analyzed 8
Figure S3. Periodic table displaying the 2018 global total attributable material extracted, after adjusting for global coverage 8
Figure S4. Map of the global distribution of aluminum operations and bar plot of cumulative share of total global aluminum production 11
Figure S5. Map of the global distribution of chromium operations and bar plot of cumulative share of total global chromium production. 12
Figure S6. Map of the global distribution of cobalt operations and bar plot of cumulative share of total global cobalt production 13
Figure S7. Map of the global distribution of gallium operations and bar plot of cumulative share of total global gallium production 14
Figure S8. Map of the global distribution of gold operations and bar plot of cumulative share of total global gold production 15
Figure S9. Map of the global distribution of iridium operations and bar plot of cumulative share of total global iridium production 16
Figure S10. Map of the global distribution of iron operations and bar plot of cumulative share of total global iron production. 17
Figure S11. Map of the global distribution of hard-rock lithium operations and bar plot of cumulative share of total global hard-rock lithium production. . 18
Figure S12. Map of the global distribution of hard-rock magnesium operations and bar plot of cumulative share of total global hard-rock magnesium production. 19
Figure S13. Map of the global distribution of molybdenum operations and bar plot of cumulative share of total global molybdenum production. 20
Figure S14. Map of the global distribution of nickel operations and bar plot of cumulative share of total global nickel production 21
Figure S15. Map of the global distribution of palladium operations and bar plot of cumulative share of total global palladium production 22
Figure S16. Map of the global distribution of platinum operations and bar plot of cumulative share of total global platinum production 23
Figure S17. Map of the global distribution of rhodium operations and bar plot of cumulative share of total global rhodium production 24
Figure S18. Map of the global distribution of ruthenium operations and bar plot of cumulative share of total global ruthenium production. 25
Figure S19. Map of the global distribution of silicon operations and bar plot of cumulative share of total global silicon production 26
Figure S20. Map of the global distribution of silver operations and bar plot of cumulative share of total global silver production 27
Figure S21. Map of the global distribution of tantalum operations and bar plot of cumulative share of total global tantalum production 28
Figure S22. Map of the global distribution of tin operations and bar plot of cumulative share of total global tin production 29
Figure S23. Map of the global distribution of titanium operations and bar plot of cumulative share of total global titanium production 30
Figure S24. Map of the global distribution of tungsten operations and bar plot of cumulative share of total global tungsten production 31
Figure S25. Map of the global distribution of vanadium operations and bar plot of cumulative share of total global vanadium production. 32
Figure S26. Map of the global distribution of zinc operations and bar plot of cumulative share of total global zinc production 33
Figure S27. Map of the global distribution of zirconium operations and bar plot of cumulative share of total global zirconium production 34
Figure S28. Rock-to-metal ratio versus ore grade for aluminum, gold, molybdenum, ruthenium, and titanium by individual operation. 35
Figure S29. Rock-to-metal ratio versus ore grade chromium, iridium, nickel, silicon, and tungsten by individual operation. 36
Figure S30. Rock-to-metal ratio versus ore grade cobalt, iron, palladium, silver, and vanadium by individual operation 37
Figure S31. Rock-to-metal ratio versus ore grade copper, lithium, platinum, tantalum, and zinc 38
Figure S32. Rock-to-metal ratio versus ore grade gallium, magnesium, rhodium, tin, and zirconium by individual operation 39
Figure S33. Contribution of each factor to the overall RMR expressed as a percentage 41

Methodological details

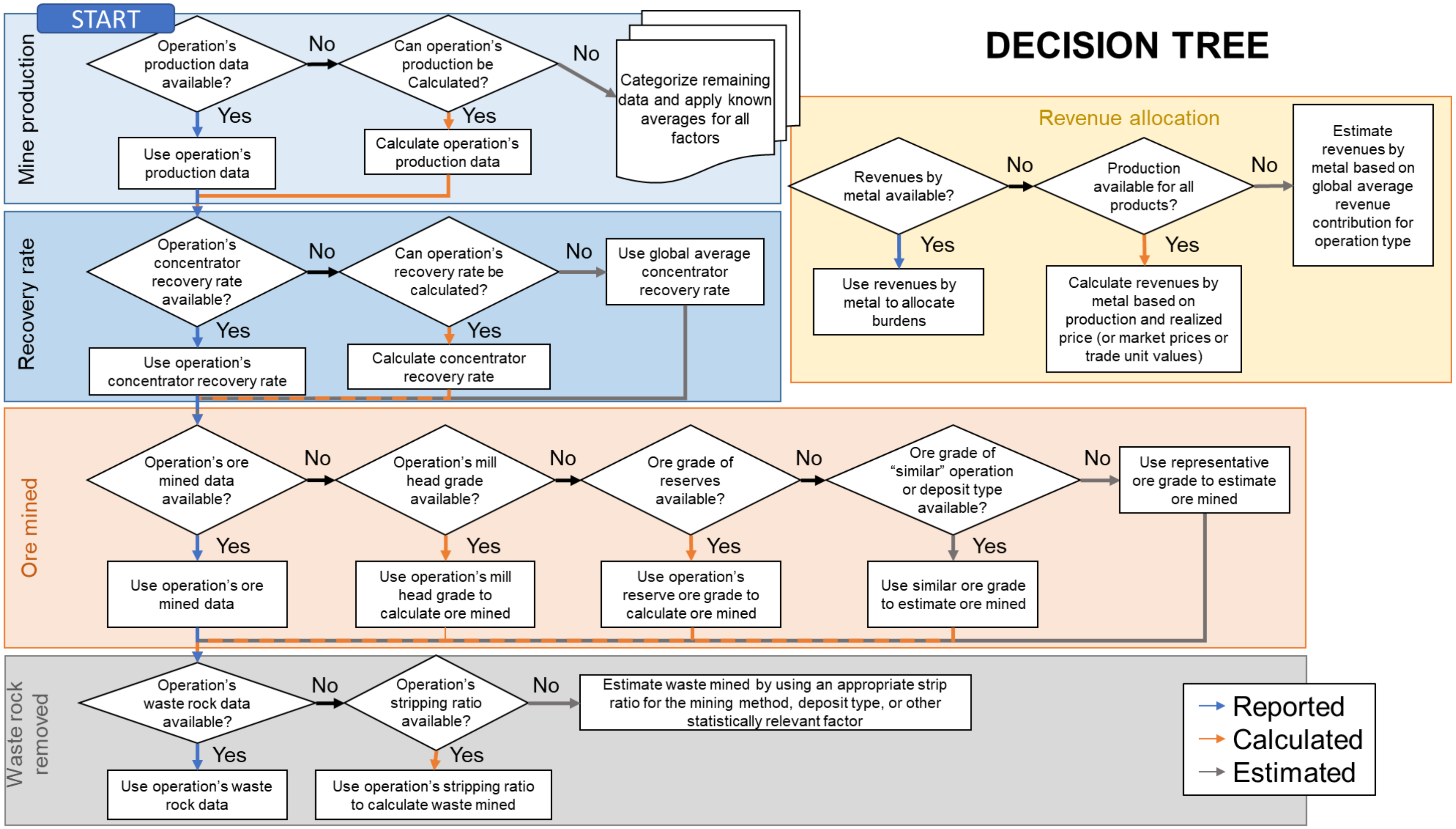

Figure S1. Decision tree for data selection and calculations.

Table S1. Number of operations, percent of global coverage, and descriptions of end products for each mineral commodity analyzed.

Mineral commodity	***Number of operations*** *(n)*	***Coverage*** *(% of global production)*	***End products***
Aluminum	68	93%	Refined aluminum
Chromium	23	100%	Industrial, metallurgical grade chromite
Cobalt	47	76%	Cobalt metal and chemicals
Copper	431	94%	Refined copper
Gallium	4	99%	Low-purity gallium
Gold	777	79%	Refined gold
Iridium	20	97%	Iridium metal
Iron	428	78%	Iron (DRI, not steel)
Lithium	16	100%	Lithium chemicals (excludes brines)
Magnesium	50	90%	Magnesium metal (excludes compounds & brines)
Molybdenum	67	100%	Technical grade molybdic oxide
Nickel	69	100%	Refined nickel
Palladium	32	93%	Palladium metal
Platinum	35	94%	Platinum metal
Rhodium	23	98%	Rhodium metal
Ruthenium	21	96%	Ruthenium metal
Silicon	1	85%	Silicon metal (excludes ferrosilicon)
Silver	627	100%	Refined silver
Tantalum	14	100%	Tantalum metal & chemicals (excludes tin slags)
Tin	43	100%	Refined tin
Titanium	35	90%	Titanium metal and oxide
Tungsten	64	99%	Tungsten metal and chemicals (mostly APT)
Vanadium	9	99%	Vanadium metal and chemicals (excludes coal stone)
Zinc	284	78%	Refined zinc
Zirconium	19	97%	Zirconium metal & chemicals

Table S2. Refinery recovery rates utilized in this analysis, for select commodities for which more representative data were available. Default value of 90% was used elsewhere.

COMMODITY	REFINERY RECOVERY RATE UTILIZED IN THIS ANALYSIS (percent)	REFERENCE	NOTES
Aluminum	98	1	
Gallium	95, 88	2	
Iridium	92.2	3	
Iron	92	4	
Molybdenum	95	5	Middle range of hydro- and pyro-metallurgy processes
Palladium	95.5	3	
Platinum	95.5	3	
Rhodium	94.1	3	
Ruthenium	95	3	
Silicon	79.6	6	
Silver	95	7	
Tantalum	90, except for Pitinga 88	8	Recovery rate for Pitinga calculated from MINSUR Annual Report 2018
Tin	90, except for Pitinga 95; Timah 91; San Rafael 97	8,9	Recovery rate for Pitiniga and San Rafael calculated from MINSUR Annual Report 2018; Recovery rate for PT Timah calculated from Annual Report 2018
Titanium	90, except for Chengde Tianbao, Desheng, and Sichuan Panzhihua 92	4	The recovery rate of 92 for iron was utilized for these three operations, because they are recovered during steel making.
Vanadium	90, except for Chengde Tianbao, Desheng, Chuanwei and Jianlong 92; Sichuan Panzhihua and Kachkanarsky GOK 72	4,10	The recovery rate of 92 for iron was utilized for four operations, because they are recovered during steel making. The recovery rate of 72 for two operations was utilized based on company reported data

Table S3. Operations for which the economic allocation was based on specific revenue shares information

OPERATION	COMMODITY(IES)	NOTES	REFERENCE
Bald Hill (Australia)	Lithium, Tantalum	Revenue share calculated from reported sales, by commodity	11
Pilgangoora 1 (Australia)	Lithium, Tantalum	Revenue share calculated from reported sales, by commodity	12
Mibra (Brazil)	Lithium	Revenue share calculated from reported market price	13
Yichun (China)	Lithium	Revenue share calculated from reported market price	14
Ningdu Heyuan (China)	Lithium	Revenue share calculated from reported sales, by commodity	15
Alvarroes (Portugal)	Lithium	Revenue share estimated from similar Li operation (Ningdu Heyuan)	15
Kachkanarsky GOK (Russia)	Vanadium	Revenue share calculated from reported sales, by commodity	10

Description of RMR results

Table S4. Summary of results for each component of the rock-to-metal ratio by mineral commodity.

Mineral commodity	Number of operations (n)	Coverage (% of global production)	Global quantities (million metric tons)										Global ratios				
			A* = B* + C*	B*	C*	A/A*	A = B + C	B	C	D	E	F	B/C	D/C*	E/D	F/E	A/F
			Total material extracted	Total waste rock removed	Total ore mined	Average revenue allocation	Attributable total material extracted	Attributable waste rock removed	Attributable ore mined	Commodity contained in processed ore	Commodity contained in concentrate produced	Finished commodity production	Waste to ore ratio	Ore grade	Concentrator recovery rate	Smelter/ refinery recovery rate	Rock to metal ratio
Aluminum	68	93%	535	220	314	100%	534	220	314	80.34	76.32	74.79	0.70	25.56%	95%	98%	7
Chromium	23	100%	505	344	161	93%	469	316	152	44.60	29.43	26.49	2.08	27.69%	66%	90%	18
Cobalt	47	76%	496	322	174	18%	87	59	28	2.07E-01	1.13E-01	1.02E-01	2.12	0.12%	55%	90%	859
Copper	431	94%	11,451	7,528	3,922	78%	8,881	5,771	3,110	25.18	19.22	17.32	1.86	0.64%	76%	90%	513
Gallium	4	99%	53	35	18	12%	6	4	2	6.79E-04	4.29E-04	4.07E-04	1.90	0.0037%	63%	95%	15,604
Gold	777	79%	13,870	9,819	4,051	52%	7,182	5,323	1,859	3.16E-03	2.62E-03	2.36E-03	2.86	0.00008%	83%	90%	3,046,349
Iridium	20	97%	397	276	121	2%	9	6	3	9.68E-06	7.94E-06	7.32E-06	1.97	0.00001%	82%	92%	1,253,310
Iron	428	78%	10,155	6,732	3,423	99%	10,062	6,665	3,398	1,534	1,185	1,090	1.96	44.81%	77%	92%	9
Lithium	16	100%	110	92	18	94%	103	87	16	1.06E-01	7.00E-02	6.30E-02	5.53	0.60%	66%	90%	1,634
Magnesium	50	90%	9	-	9	100%	9	-	9	1.03	1.00	0.90	0.00	11.78%	97%	90%	10
Molybdenum	67	100%	6,388	4,173	2,216	20%	1,276	831	445	4.88E-01	3.00E-01	2.85E-01	1.87	0.02%	62%	95%	4,478
Nickel	69	100%	963	586	377	57%	545	314	231	3.08	2.42	2.18	1.36	0.82%	79%	90%	250
Palladium	32	93%	556	388	168	25%	141	97	44	2.59E-04	2.14E-04	2.05E-04	2.20	0.00015%	83%	96%	688,473
Platinum	35	94%	559	391	168	27%	149	103	45	2.28E-04	1.85E-04	1.78E-04	2.28	0.00014%	81%	96%	834,932
Rhodium	23	98%	438	299	139	10%	45	31	14	2.82E-05	2.31E-05	2.17E-05	2.12	0.000020%	82%	94%	2,074,800
Ruthenium	21	96%	423	298	125	2%	7	4	2	3.87E-05	3.14E-05	2.98E-05	1.98	0.00003%	81%	95%	218,490
Silicon	1	85%	9	5	4	100%	9	5	4	4.13	3.97	3.16	1.22	98.33%	96%	80%	3
Silver	627	100%	12,812	8,977	3,834	4%	570	388	182	3.90E-02	2.68E-02	2.55E-02	2.13	0.0010%	69%	95%	22,378
Tantalum	14	100%	57	38	18	30%	17	7	10	1.34E-02	2.14E-03	1.91E-03	0.72	0.073%	16%	90%	8,946
Tin	43	100%	657	24	633	99%	650	20	630	4.54E-01	3.20E-01	2.91E-01	0.03	0.072%	70%	91%	2,231
Titanium	35	90%	592	119	473	61%	359	34	325	7.54	4.04	3.64	0.11	1.59%	54%	90%	99
Tungsten	64	99%	115	69	46	68%	78	45	33	1.10E-01	8.03E-02	7.23E-02	1.34	0.24%	73%	90%	1,081
Vanadium	9	99%	380	274	106	19%	73	53	21	1.32E-01	7.02E-02	5.50E-02	2.53	0.12%	53%	78%	1,336
Zinc	284	78%	1,552	1,087	465	40%	625	444	181	11.83	9.78	8.80	2.45	2.55%	83%	90%	71
Zirconium	19	97%	356	31	325	47%	167	17	151	8.19E-01	6.75E-01	6.07E-01	0.11	0.25%	82%	90%	275
Overall[a]	1,928[b]						32,055	20,845	11,210	1,714	1,333	1,229	1.86	8.04%	78%	92%	26

[a] Does not adjust for global coverage

[b] Number of unique operations or country remainders

Table S5. Summary statistics for the rock-to-metal ratio (RMR) by mineral commodity

Mineral commodity	***Number of operations*** *(n)*	***Coverage*** *(% of global production)*	***Minimum***	***25th percentile***	***Arithmetic mean***	***Median***	***Production-weighted mean*** *($\bar{x}$)*	***75th percentile***	***Maximum***	***Standard deviation***
Aluminum	68	93%	4.63E+00	5.64E+00	8.56E+00	6.77E+00	7.14E+00	1.01E+01	2.23E+01	4.49E+00
Chromium	23	100%	1.32E+01	1.51E+01	1.77E+01	1.68E+01	1.77E+01	1.92E+01	2.85E+01	3.70E+00
Cobalt	47	76%	1.90E+02	4.65E+02	2.04E+03	8.10E+02	8.59E+02	2.16E+03	1.54E+04	3.08E+03
Copper	431	94%	2.34E+00	1.81E+02	5.64E+02	3.87E+02	5.13E+02	6.90E+02	1.67E+04	9.44E+02
Gallium	4	99%	3.75E+02	5.49E+03	7.69E+03	7.26E+03	1.56E+04	9.46E+03	1.59E+04	6.35E+03
Gold	777	79%	1.04E+05	8.31E+05	3.34E+06	1.68E+06	3.05E+06	3.92E+06	2.21E+08	8.67E+06
Iridium	20	97%	3.37E+05	9.43E+05	1.43E+06	1.11E+06	1.25E+06	1.38E+06	5.19E+06	1.13E+06
Iron	428	78%	1.47E+00	1.18E+01	1.73E+01	1.33E+01	9.23E+00	1.98E+01	1.02E+02	1.26E+01
Lithium	16	100%	2.68E+02	5.81E+02	1.76E+03	1.06E+03	1.63E+03	1.71E+03	1.09E+04	2.55E+03
Magnesium	50	90%	8.69E+00	8.69E+00	9.34E+00	8.69E+00	9.73E+00	8.69E+00	2.04E+01	2.45E+00
Molybdenum	67	100%	5.77E+02	1.47E+03	3.91E+03	2.34E+03	4.48E+03	4.3E+03	5.25E+04	6.52E+03
Nickel	69	100%	1.52E+01	1.34E+02	3.29E+02	2.41E+02	2.50E+02	3.44E+02	2.09E+03	3.42E+02
Palladium	32	93%	1.55E+05	5.60E+05	1.17E+06	8.52E+05	6.88E+05	1.36E+06	5.22E+06	1.05E+06
Platinum	35	94%	1.32E+05	5.55E+05	1.27E+06	7.73E+05	8.35E+05	1.77E+06	4.51E+06	1.18E+06
Rhodium	23	98%	3.33E+05	1.40E+06	2.38E+06	1.82E+06	2.07E+06	2.20E+06	1.12E+07	2.26E+06
Ruthenium	21	96%	3.43E+04	1.75E+05	2.73E+05	2.04E+05	2.18E+05	2.45E+05	1.23E+06	2.57E+05
Silicon	1	85%	2.95E+00	2.95E+00	2.95E+00	2.95E+00	2.95E+00	2.95E+00	2.95E+00	Not applicable
Silver	627	100%	1.61E+03	8.92E+03	6.71E+04	1.77E+04	2.24E+04	4.48E+04	2.11E+07	8.41E+05
Tantalum	14	100%	2.13E+03	5.06E+03	1.85E+04	5.06E+03	8.95E+03	9.79E+03	8.28E+04	2.78E+04
Tin	43	100%	4.59E+01	1.59E+02	7.47E+02	2.74E+02	2.23E+03	3.51E+02	7.58E+03	1.60E+03
Titanium	35	90%	5.47E+00	6.05E+01	1.07E+02	1.04E+02	9.87E+01	1.39E+02	2.96E+02	5.39E+01
Tungsten	64	99%	9.34E+01	3.74E+02	8.37E+02	7.20E+02	1.08E+03	8.89E+02	3.69E+03	6.63E+02
Vanadium	9	99%	7.46E+02	9.78E+02	1.23E+03	1.14E+03	1.34E+03	1.21E+03	2.57E+03	5.37E+02
Zinc	284	78%	1.10E+01	4.23E+01	7.89E+01	6.43E+01	7.10E+01	9.83E+01	4.30E+02	5.85E+01
Zirconium	19	97%	1.01E+02	1.79E+02	2.80E+02	2.34E+02	2.75E+02	3.14E+02	5.81E+02	1.46E+02

Table S6. Total material mined, waste rock removed, and ore mined by mineral commodity after adjusting for global coverage

	**Coverage** (% of global production)	**Coverage-adjusted attributable total material extracted** (Million metric tons)	**Coverage-adjusted attributable waste rock removed** (Million metric tons)	**Coverage-adjusted attributable ore mined** (Million metric tons)
Aluminum	93%	5.76E+02	2.37E+02	3.38E+02
Chromium	100%	4.69E+02	3.17E+02	1.53E+02
Cobalt	76%	1.14E+02	7.76E+01	3.67E+01
Copper	94%	9.42E+03	6.12E+03	3.30E+03
Gallium	99%	6.44E+00	4.22E+00	2.22E+00
Gold	79%	9.07E+03	6.72E+03	2.35E+03
Iridium	97%	9.45E+00	6.27E+00	3.19E+00
Iron	78%	1.29E+04	8.53E+03	4.35E+03
Lithium	100%	1.03E+02	8.71E+01	1.58E+01
Magnesium	90%	9.69E+00	0.00E+00	9.69E+00
Molybdenum	100%	1.28E+03	8.31E+02	4.45E+02
Nickel	100%	5.45E+02	3.14E+02	2.31E+02
Palladium	93%	1.52E+02	1.04E+02	4.76E+01
Platinum	94%	1.59E+02	1.10E+02	4.83E+01
Rhodium	98%	4.60E+01	3.13E+01	1.47E+01
Ruthenium	96%	6.81E+00	4.52E+00	2.28E+00
Silicon	85%	1.10E+01	6.02E+00	4.94E+00
Silver	100%	5.73E+02	3.90E+02	1.83E+02
Tantalum	100%	1.71E+01	7.15E+00	9.98E+00
Tin	100%	6.50E+02	1.99E+01	6.30E+02
Titanium	90%	3.97E+02	3.81E+01	3.59E+02
Tungsten	99%	7.90E+01	4.52E+01	3.38E+01
Vanadium	99%	7.44E+01	5.33E+01	2.11E+01
Zinc	78%	7.97E+02	5.66E+02	2.31E+02
Zirconium	97%	1.73E+02	1.73E+01	1.56E+02
Sum		**3.76E+04**	**2.46E+04**	**1.30E+04**

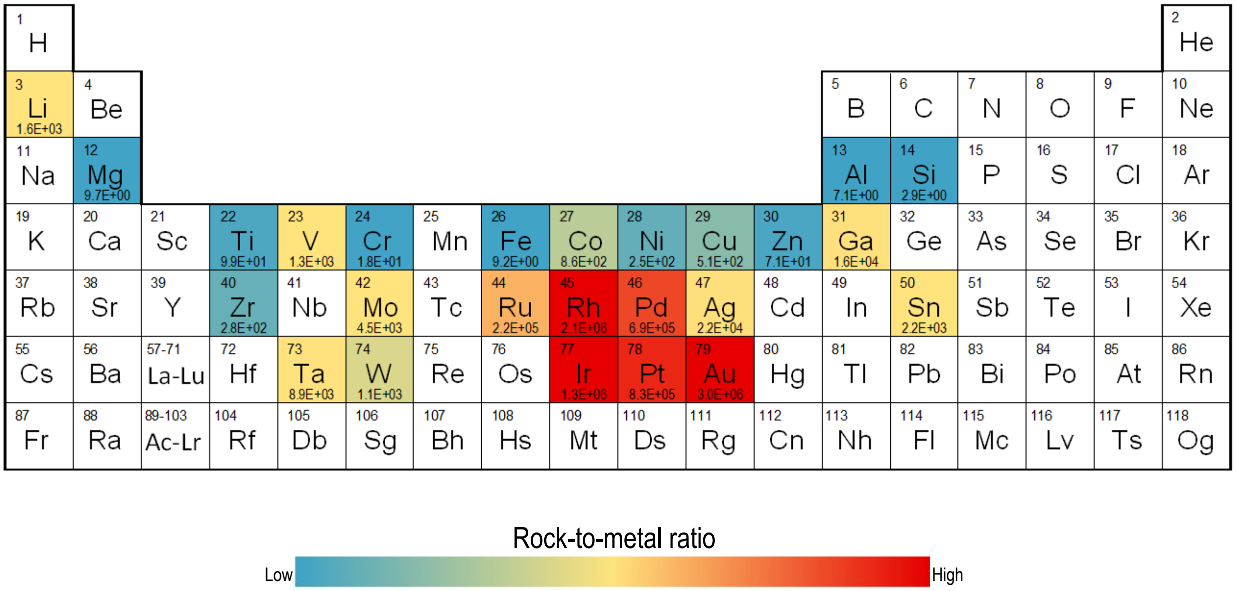

Figure S2. *Periodic table displaying the 2018 global RMR for the mineral commodities analyzed*

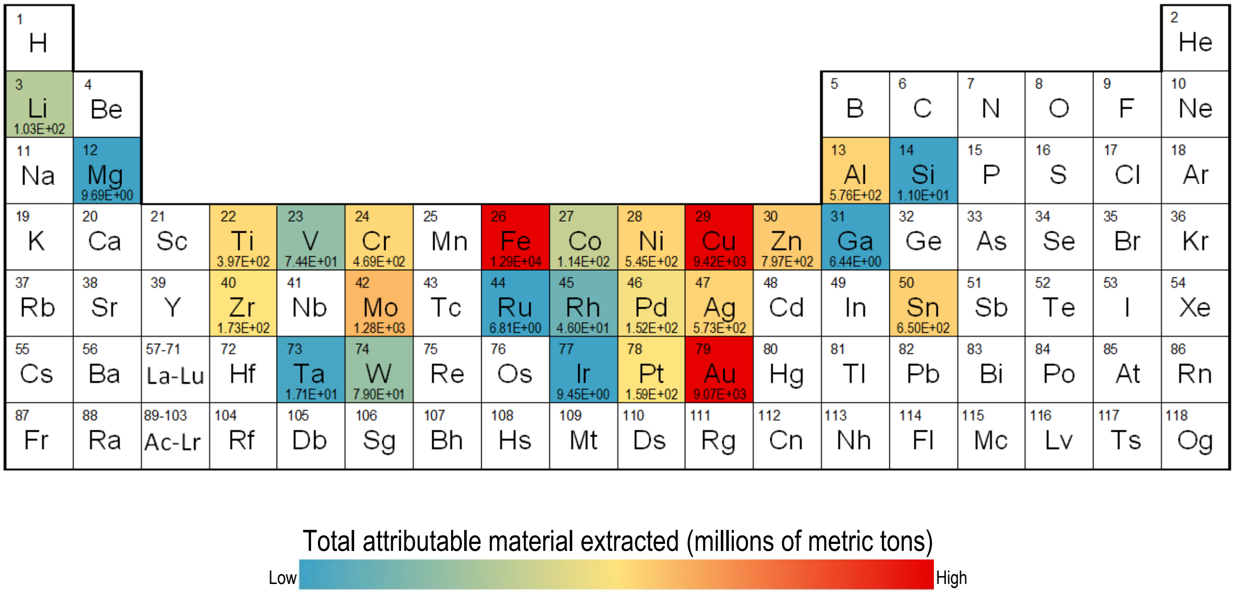

Figure S3. Periodic table displaying the 2018 global total attributable material extracted (ore mined and waste rock removed) in millions of metric tons for the commodities analyzed, after adjusting for global coverage

Table S7. Percentages of global production with rock-to-metal ratio (RMR) values equal to or less than noted levels

Mineral commodity	Percentages of global production with RMR values equal to or less than…								
	5%	10%	25%	33.3%	50%	66.6%	75%	90%	95%
Aluminum	5.5E+00	5.5E+00	5.5E+00	5.8E+00	6.2E+00	7.3E+00	9.3E+00	1.4E+01	Not available
Chromium	1.3E+01	1.3E+01	1.4E+01	1.7E+01	1.7E+01	1.9E+01	1.9E+01	2.3E+01	2.9E+01
Cobalt	2.0E+02	2.0E+02	3.2E+02	4.8E+02	6.9E+02	1.5E+03	3.2E+03	Not available	Not available
Copper	6.9E+01	1.1E+02	2.0E+02	3.0E+02	3.9E+02	6.4E+02	7.3E+02	1.5E+03	Not available
Gallium	1.6E+04	1.6E+04	1.6E+04	1.6E+04	1.6E+04	1.6E+04	1.6E+04	1.6E+04	1.6E+04
Gold	2.9E+05	5.3E+05	1.1E+06	1.5E+06	3.1E+06	5.5E+06	8.6E+06	Not available	Not available
Iridium	3.8E+05	3.8E+05	9.5E+05	1.1E+06	1.1E+06	1.2E+06	1.3E+06	2.6E+06	5.2E+06
Iron	3.4E+00	4.0E+00	6.9E+00	7.1E+00	8.6E+00	1.3E+01	2.1E+01	Not available	Not available
Lithium	4.3E+02	4.3E+02	4.3E+02	6.3E+02	1.6E+03	1.7E+03	1.9E+03	2.5E+03	2.8E+03
Magnesium	8.7E+00	8.7E+00	8.7E+00	8.7E+00	8.7E+00	8.7E+00	8.7E+00	2.0E+01	Not available
Molybdenum	9.3E+02	9.9E+02	1.8E+03	2.4E+03	3.3E+03	4.9E+03	8.8E+03	9.3E+03	9.3E+03
Nickel	2.1E+01	4.6E+01	1.0E+02	1.8E+02	2.9E+02	3.0E+02	3.0E+02	3.0E+02	3.0E+02
Palladium	1.6E+05	2.3E+05	2.7E+05	2.7E+05	3.0E+05	8.6E+05	9.5E+05	2.1E+06	Not available
Platinum	1.9E+05	2.3E+05	2.6E+05	6.5E+05	7.5E+05	8.1E+05	1.3E+06	2.3E+06	Not available
Rhodium	6.4E+05	6.5E+05	1.2E+06	1.7E+06	1.9E+06	2.0E+06	2.2E+06	4.4E+06	5.6E+06
Ruthenium	7.1E+04	7.1E+04	1.3E+05	2.0E+05	2.1E+05	2.3E+05	2.5E+05	3.6E+05	6.2E+05
Silicon	2.9E+00	2.9E+00	2.9E+00	2.9E+00	2.9E+00	2.9E+00	2.9E+00	Not available	Not available
Silver	3.5E+03	4.6E+03	8.2E+03	8.7E+03	1.4E+04	1.7E+04	2.2E+04	5.4E+04	8.5E+04
Tantalum	5.1E+03	5.1E+03	5.1E+03	5.1E+03	5.1E+03	5.1E+03	6.0E+03	1.1E+04	3.7E+04
Tin	6.2E+01	6.3E+01	1.0E+02	1.6E+02	3.0E+02	1.1E+03	7.6E+03	7.6E+03	7.6E+03
Titanium	5.5E+00	5.5E+00	6.8E+01	8.5E+01	1.2E+02	1.4E+02	1.4E+02	3.0E+02	Not available
Tungsten	1.7E+02	2.7E+02	4.4E+02	5.3E+02	8.9E+02	8.9E+02	1.9E+03	2.4E+03	2.4E+03
Vanadium	8.3E+02	8.3E+02	8.3E+02	8.3E+02	9.8E+02	1.2E+03	2.6E+03	2.6E+03	2.6E+03
Zinc	1.8E+01	1.8E+01	3.3E+01	4.3E+01	6.8E+01	1.3E+02	1.9E+02	Not available	Not available
Zirconium	1.0E+02	1.0E+02	1.7E+02	1.8E+02	2.7E+02	3.2E+02	3.2E+02	5.6E+02	5.6E+02

Precious metals: Gold RMRs for individual operations vary significantly, spanning several orders of magnitude from 1.0×10^5 to 2.2×10^8, and a global, production-weighted mean, ($\bar{x}$) RMR of 3.0×10^6 (n = 777). The one operation with extremely high RMR value of 2.2×10^8, which is the highest RMR of any commodity-operation analyzed, is a result of very low ore grade (0.016 ppm Au) combined with a revenue allocation of 100%. RMR for the platinum-group metals mainly range (minimum and maximum of across their individual interquartile ranges) from 1.7×10^5 to 2.2×10^6. Global RMR are highest for rhodium ($\bar{x} = 2.1 \times 10^6$), followed by iridium ($\bar{x} = 1.3 \times 10^6$) and platinum ($\bar{x} = 8.3 \times 10^5$), and lowest for palladium ($\bar{x} = 6.9 \times 10^5$) and ruthenium ($\bar{x} = 2.2 \times 10^5$) due mainly to a combination of revenue allocation and ore grades. Silver has the lowest global RMRs of the precious metals ($\bar{x} = 2.2 \times 10^4$; n = 627), and individual RMRs ranging from 1.6×10^3 to 2.1×10^7. The extremely high RMR for one operation is a consequence of relatively low ore grade (0.5 ppm Ag), low concentrator recovery rate (30%) and 100% revenue allocation.

Ferrous and nonferrous metals: Molybdenum RMRs mostly range (interquartile) between 1.5×10^3 and 4.3×10^3, with a global RMR of 4.5×10^3 (n = 67). Vanadium RMRs vary (minimum and maximum) from 7.5×10^2 to 2.6×10^3 ($\bar{x} = 1.3 \times 10^3$; n = 9), with the global RMR heavily influenced by China and Russia operations which produce roughly 80% of the global primary vanadium. Similarly, the tungsten's global RMR ($\bar{x} = 1.1 \times 10^3$; n = 64) is notably influenced by China operations, which account for nearly 80% of global production, and individual ratios span from 93 to 3.7×10^3. Copper RMRs mainly range (interquartile) from 1.8×10^2 to 6.9×10^2 ($\bar{x} = 5.1 \times 10^2$; n = 431), with one China operation having an outlier ratio of 1.7×10^4, due to a low grade (0.04% Cu), low concentrator recovery rate (50%) and high revenue allocation (100%). Tin RMRs range (minimum and maximum) from 46 to 7.6×10^3 ($\bar{x} = 2.2 \times 10^3$; n = 43), with the global RMR heavily influenced by a single Indonesia operation (the leading global tin producer having the largest individual RMR). Nickel RMRs range (minimum and maximum) between 15 and 2.1×10^3 ($\bar{x} = 2.5 \times 10^2$; n=69), with 90% of global production having a ratio below 3.0×10^2. Zinc RMRs range (minimum and maximum) from 11 to 4.3×10^2 ($\bar{x} = 71$; n = 284), with 75% of global production having a ratio below 1.9×10^2. Chromium RMRs range (minimum and maximum) from 13 to 29 ($\bar{x} = 18$; n = 23), with 75% of global production operations having ratios below 19. Iron includes the lowest calculated individual operation RMR at 1.5 and range up to 1.0×10^2 ($\bar{x} = 9.2$; n = 428), with 75% of global production having a ratio below 20. Aluminum RMRs range (minimum and maximum) from 4.6 to 22 ($\bar{x} = 7.1$; n = 68), with 75% of global production having an RMR below 9.3. Cobalt RMRs range (interquartile) from 4.6×10^2 to 2.2×10^3 ($\bar{x} = 8.6 \times 10^2$; n = 47), with two outliers extending the range up to 1.5×10^4, and the global RMR significantly influenced by six operations in Congo (Kinshasa) that account for nearly half of global cobalt production.

Minor metals and other commodities: Tantalum RMR for individual operations range (minimum and maximum) from 2.1×10^3 to 8.3×10^4 ($\bar{x} = 8.9 \times 10^3$; n = 14), with 90% production having an RMR below 1.1×10^4. Gallium RMRs, which were calculated at the country level, range from 3.8×10^2 to 1.6×10^4 ($\bar{x} = 1.6 \times 10^4$; n = 4), with the global RMR mainly reflecting Chinese operations that account for approximately 96% of global gallium production. The hard-rock lithium global RMR ($\bar{x} = 1.6 \times 10^3$; n = 16) is largely controlled by Australian operations which account for 89% of global production from lithium hard-rock sources (brine sources are excluded from our calculation), and ratios for individual operations range from 2.7×10^2 to 1.1×10^4. Zirconium RMRs range (minimum and maximum) from 1.0×10^2 to 5.8×10^2 ($\bar{x} = 2.8 \times 10^2$; n = 19), with more than 75% of global production having a ratio below 3.2×10^2. Titanium RMRs mostly range (interquartile) from 60 and 1.4×10^2 ($\bar{x} = 99$; n = 35), with two outlier operations extending the full range to 5.5 and 3.0×10^2, mainly due to ore grades. Magnesium RMRs are relatively well-constrained between 8.7 and 20 ($\bar{x} = 9.7$; n = 50) largely due to the similarity in the ore sources (carbonate minerals; brine sources are excluded from our calculation) and the dissolution-mining methods (which generate negligible mined waste) across all operations, with a visible bimodal distribution in RMR between dolomite ($<\bar{x}$) and carnallite ($>\bar{x}$) operations. We estimate a RMR of 2.9 for silicon metal, calculated as a single ratio at the global level.

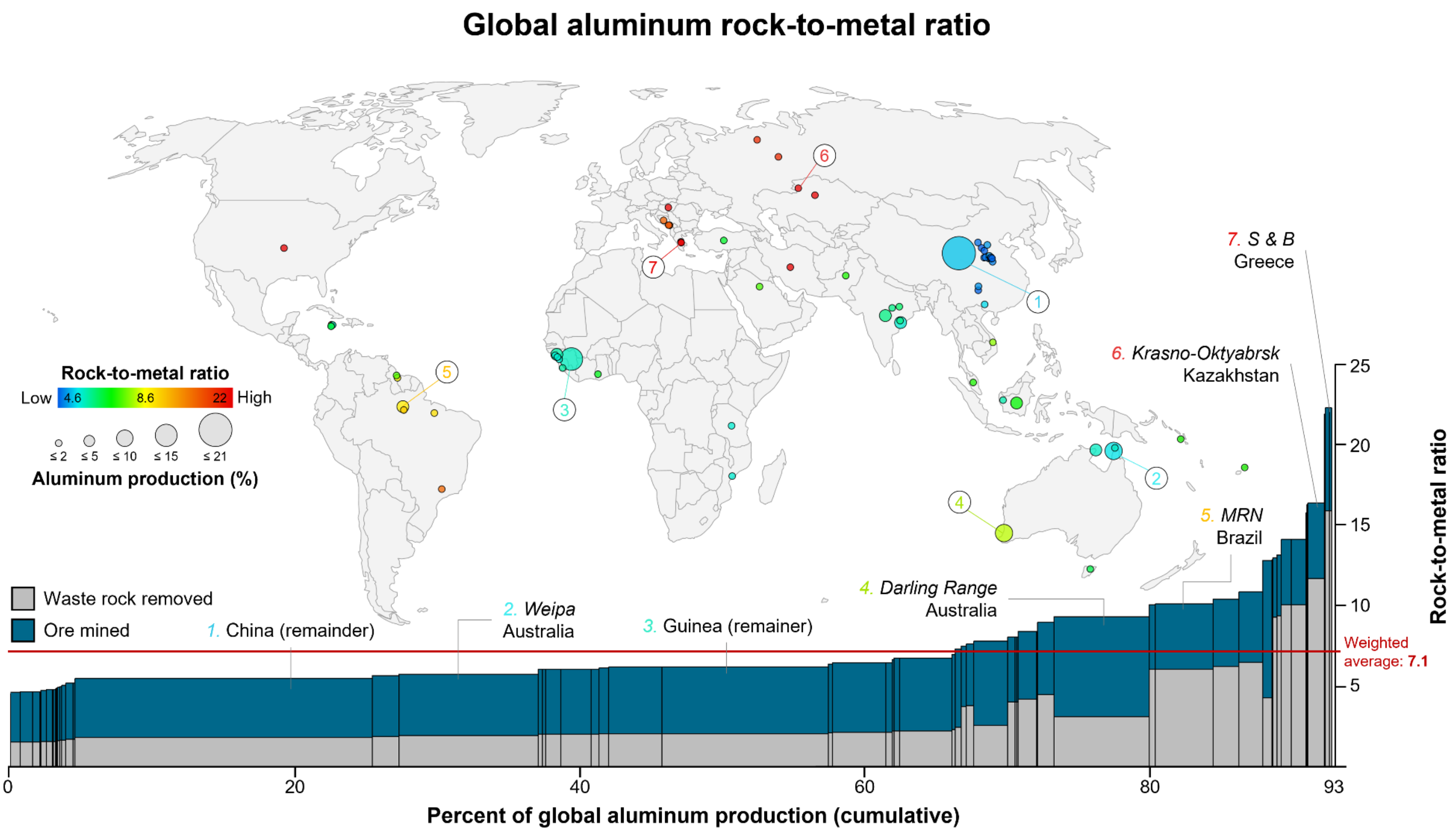

Figure S4. Map of the global distribution of aluminum operations and bar plot of cumulative share of total global aluminum production. Each individual operation is plotted as a single circle on the map and single bar on the plot. The colors of the circles indicate individual rock-to-metal ratios (RMRs), which range from a low of 4.6 to a high of 2.2×10^1 and yield a global RMR of 7.1 (n = 68). The sizes of the circles are proportional to an operation's share (in percent) of total global aluminum production, which range from a low of <0.001% to a high of 21% for a total global coverage of 93% of 2018 global aluminum production reported by the U.S. Geological Survey.[16] Operations are ordered from lowest to highest RMR on the bar plot.

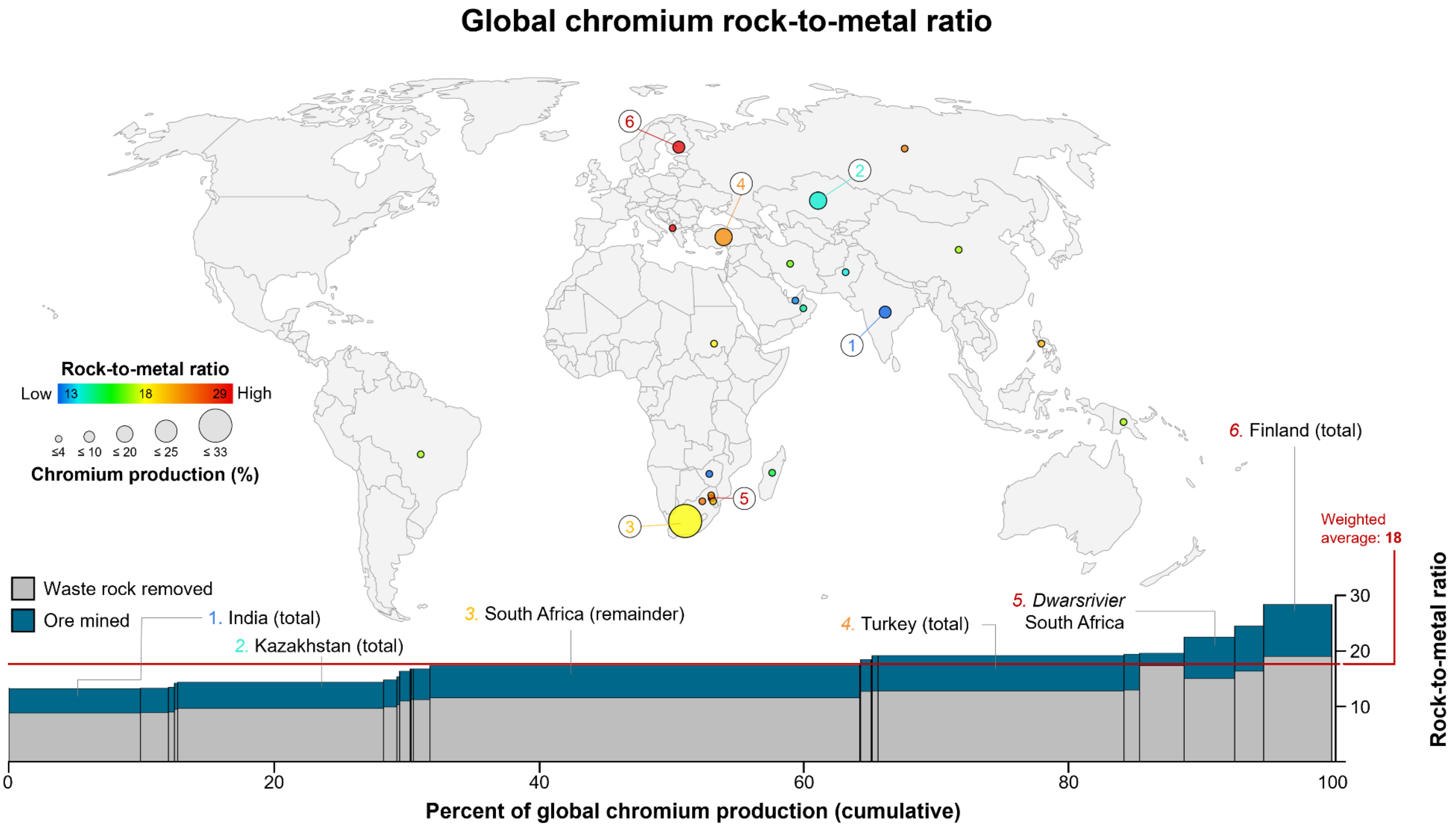

Figure S5. Map of the global distribution of chromium operations and bar plot of cumulative share of total global chromium production. Each individual operation is plotted as a single circle on the map and single bar on the plot. The colors of the circles indicate individual rock-to-metal ratios (RMRs), which range from a low of 1.3×10^1 to a high of 2.9×10^1 and yield a global RMR of 1.8×10^1 ($n = 23$). The sizes of the circles are proportional to an operation's share (in percent) of total global chromium production, which range from a low of 0.015% to a high of 32% for a total global coverage of 100% of 2018 global chromium production reported by the U.S. Geological Survey.[16] Operations are ordered from lowest to highest RMR on the bar plot.

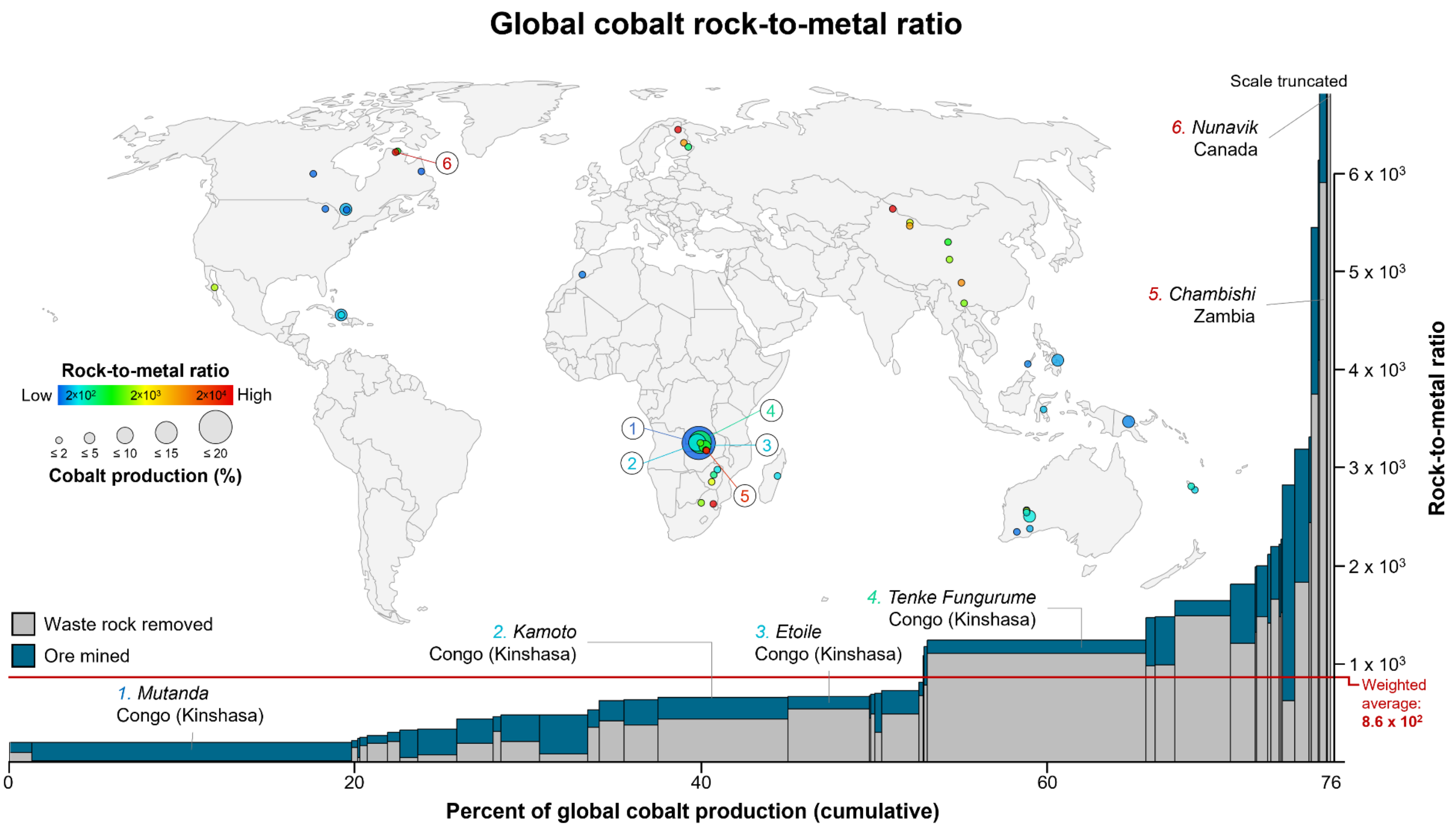

Figure S6. Map of the global distribution of cobalt operations and bar plot of cumulative share of total global cobalt production. Each individual operation is plotted as a single circle on the map and single bar on the plot. The colors of the circles indicate individual rock-to-metal ratios (RMRs), which range from a low of 1.9×10^2 to a high of 1.5×10^4 and yield a global RMR of 8.6×10^2 ($n = 47$). The sizes of the circles are proportional to an operation's share (in percent) of total global cobalt production, which range from a low of 0.008% to a high of 18.5% for a total global coverage of 76% of 2018 global cobalt production reported by the U.S. Geological Survey. [16] *Operations are ordered from lowest to highest RMR on the bar plot.*

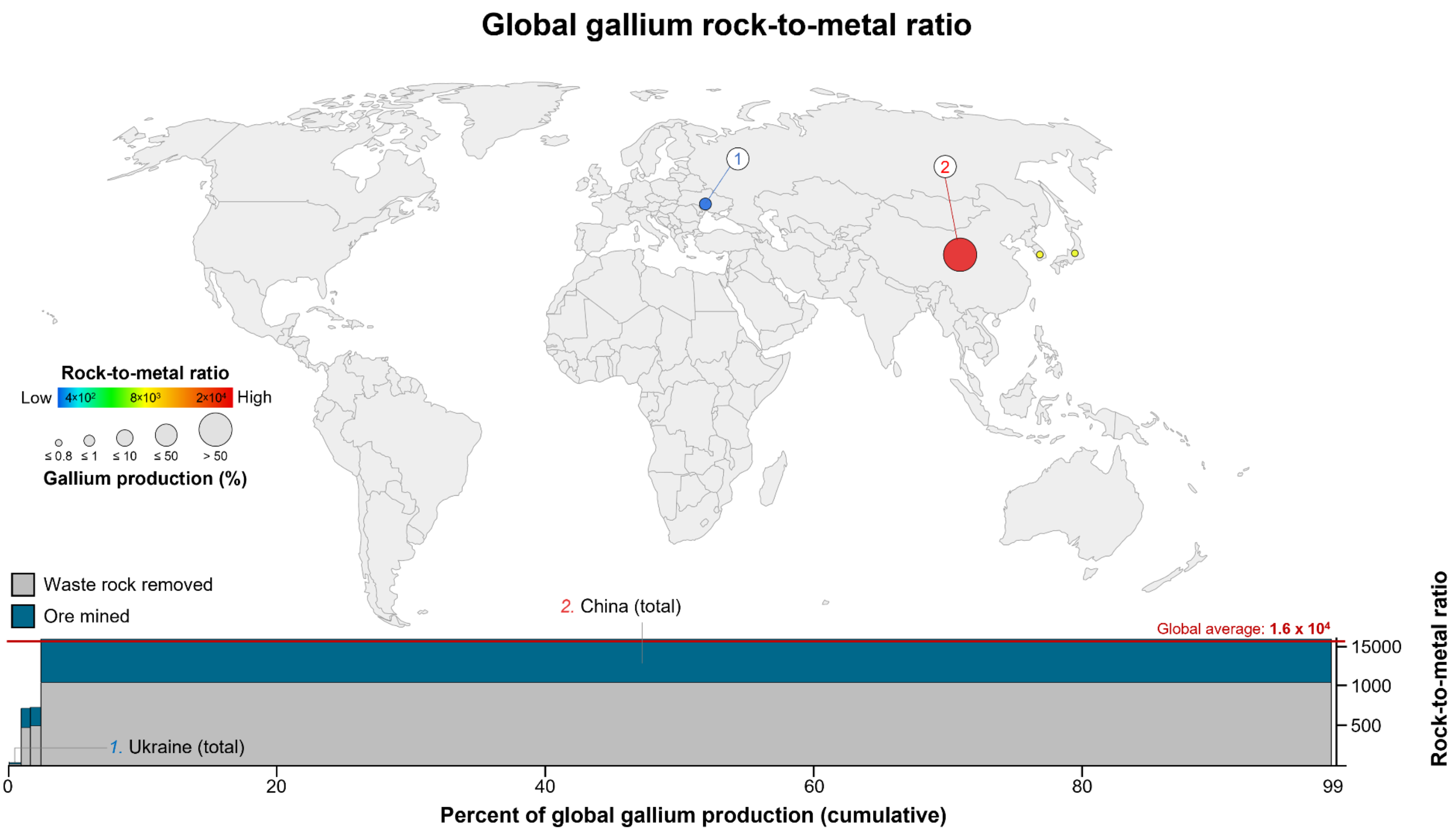

Figure S7. Map of the global distribution of gallium operations and bar plot of cumulative share of total global gallium production. Each individual operation is plotted as a single circle on the map and single bar on the plot. The colors of the circles indicate individual rock-to-metal ratios (RMRs), which range from a low of 3.8×10^2 to a high of 1.6×10^4 and yield a global RMR of 1.6×10^4 ($n = 4$). The sizes of the circles are proportional to an operation's share (in percent) of total global gallium production, which range from a low of 1% to a high of 96% for a total global coverage of 99% of 2018 global gold production reported by the U.S. Geological Survey. [16] *Operations are ordered from lowest to highest RMR on the bar plot.*

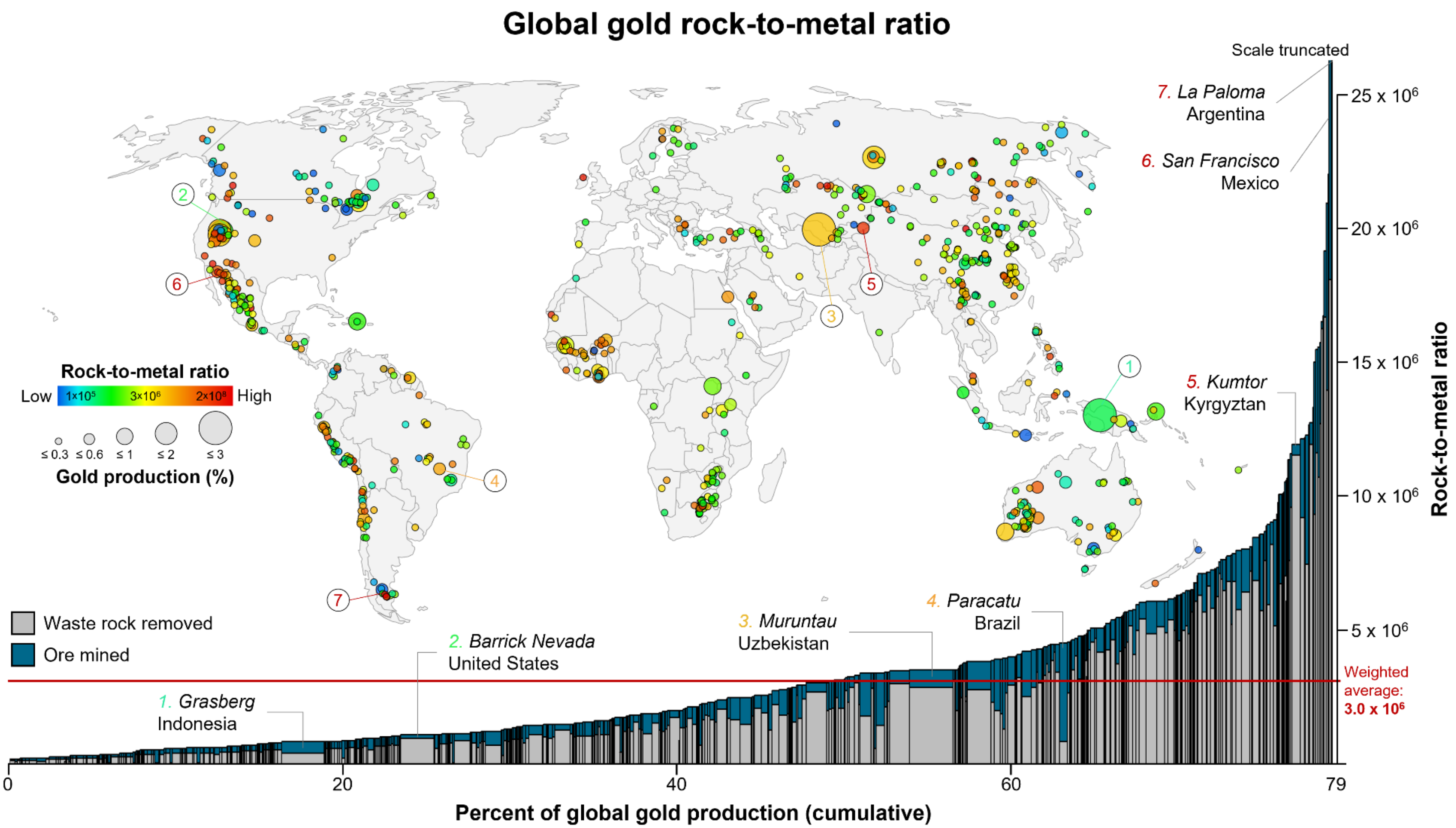

Figure S8. Map of the global distribution of gold operations and bar plot of cumulative share of total global gold production. Each individual operation is plotted as a single circle on the map and single bar on the plot. The colors of the circles indicate individual rock-to-metal ratios (RMRs), which range from a low of 1.0×10^5 to a high of 2.2×10^8 and yield a global RMR of 3.0×10^6 ($n = 777$). The sizes of the circles are proportional to an operation's share (in percent) of total global gold production, which range from a low of <0.001% to a high of 2.6% for a total global coverage of 79% of 2018 global gold production reported by the U.S. Geological Survey.[16] Operations are ordered from lowest to highest RMR on the bar plot.

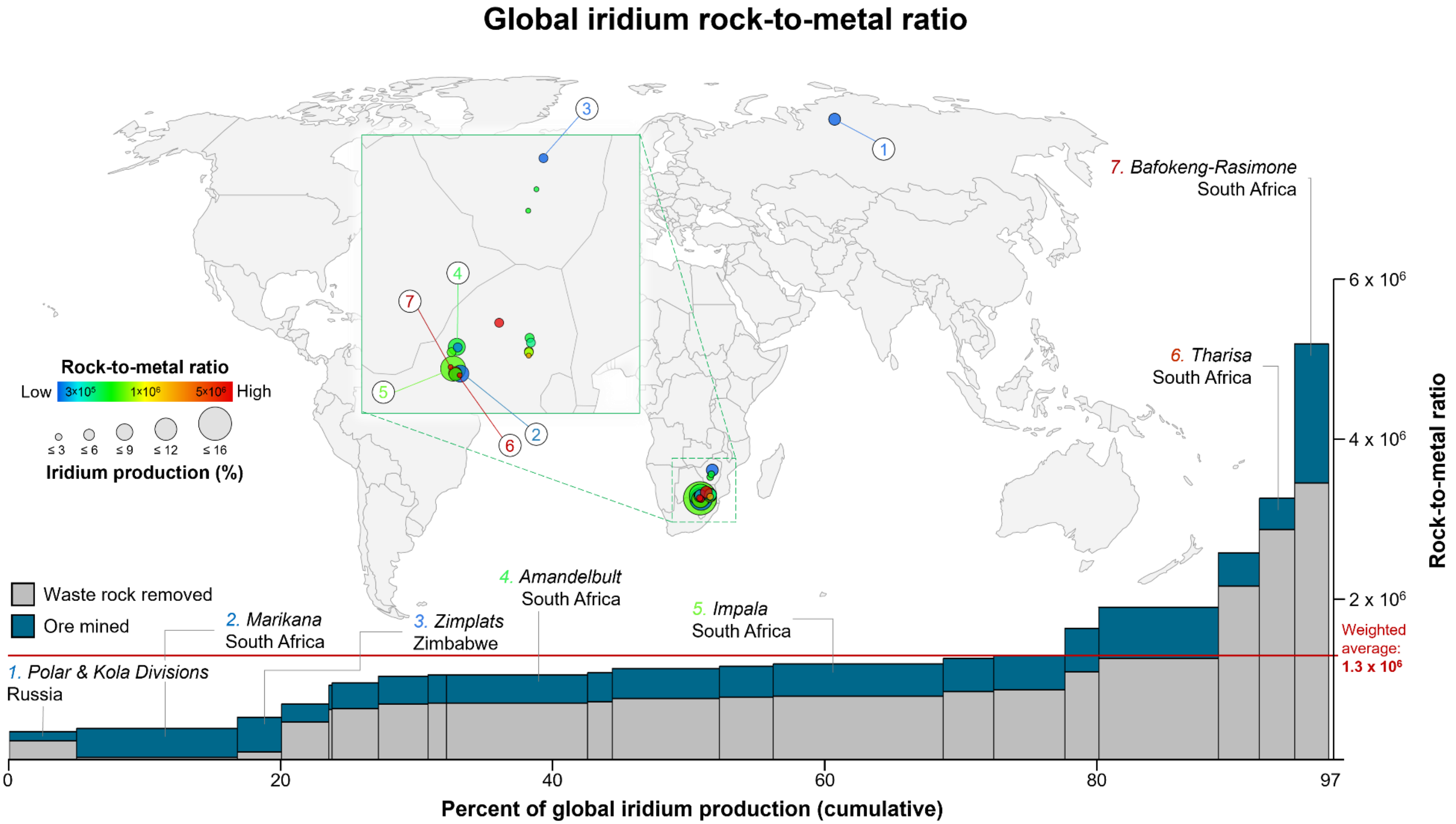

Figure S9. Map of the global distribution of iridium operations and bar plot of cumulative share of total global iridium production. Each individual operation is plotted as a single circle on the map and single bar on the plot. The colors of the circles indicate individual rock-to-metal ratios (RMRs), which range from a low of 3.4×10^5 to a high of 5.2×10^6 and yield a global RMR of 1.3×10^6 ($n = 20$). The sizes of the circles are proportional to an operation's share (in percent) of total global iridium production, which range from a low of 0.12% to a high of 12% for a total global coverage of 97% of 2018 global iridium production reported by the U.S. Geological Survey.[17] Operations are ordered from lowest to highest RMR on the bar plot.

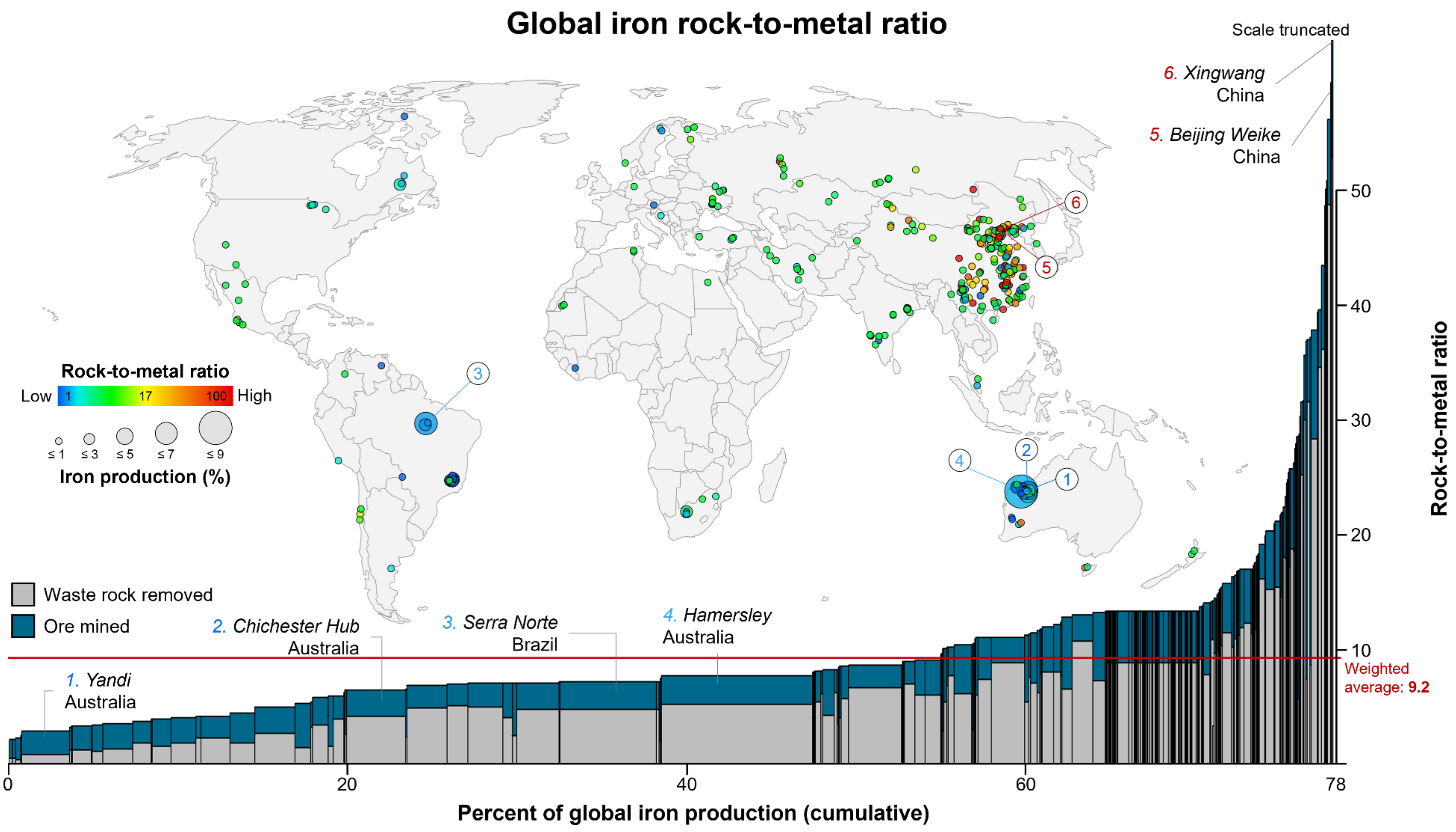

Figure S10. Map of the global distribution of iron operations and bar plot of cumulative share of total global iron production. Each individual operation is plotted as a single circle on the map and single bar on the plot. The colors of the circles indicate individual rock-to-metal ratios (RMRs), which range from a low of 1.5 to a high of 1.0 × 10^2 and yield a global RMR of 9.2 (n = 428). The sizes of the circles are proportional to an operation's share (in percent) of total global iron production, which range from a low of <0.001% to a high of 8.9% for a total global coverage of 78% of 2018 global iron production reported by the U.S. Geological Survey.[16] Operations are ordered from lowest to highest RMR on the bar plot.

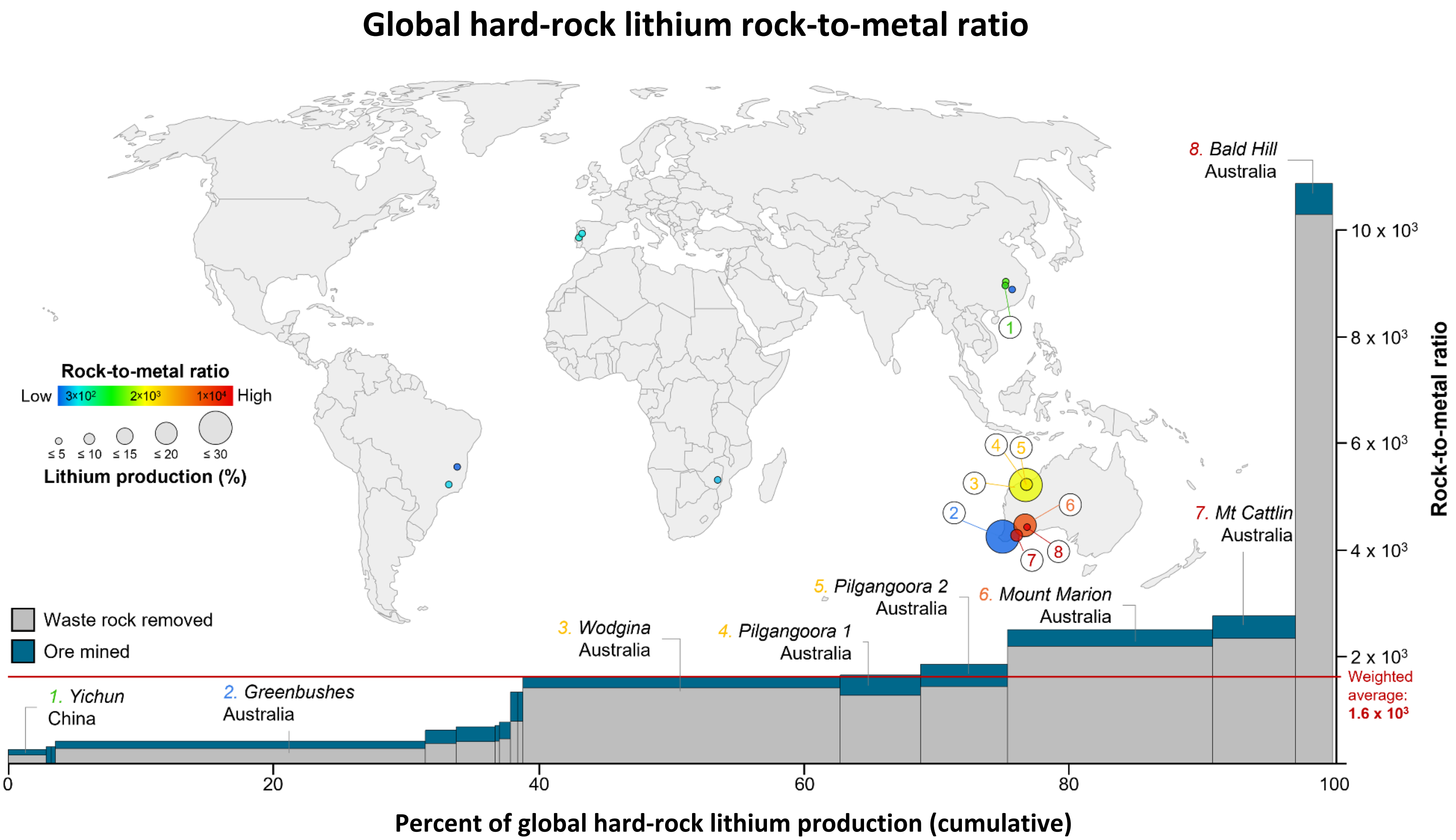

Figure S11. Map of the global distribution of hard-rock lithium operations and bar plot of cumulative share of total global hard-rock lithium production. Each individual operation is plotted as a single circle on the map and single bar on the plot. The colors of the circles indicate individual rock-to-metal ratios (RMRs), which range from a low of 2.7×10^2 to a high of 1.1×10^4 and yield a global RMR of 1.6×10^3 ($n = 16$). The sizes of the circles are proportional to an operation's share (in percent) of total global lithium production, which range from a low of <0.32% to a high of 28% for a total global coverage of 100% of 2018 global hard-rock lithium production (excluding brine) revised from that reported by the U.S. Geological Survey.[16] Operations are ordered from lowest to highest RMR on the bar plot.

Global hard-rock magnesium rock-to-metal ratio

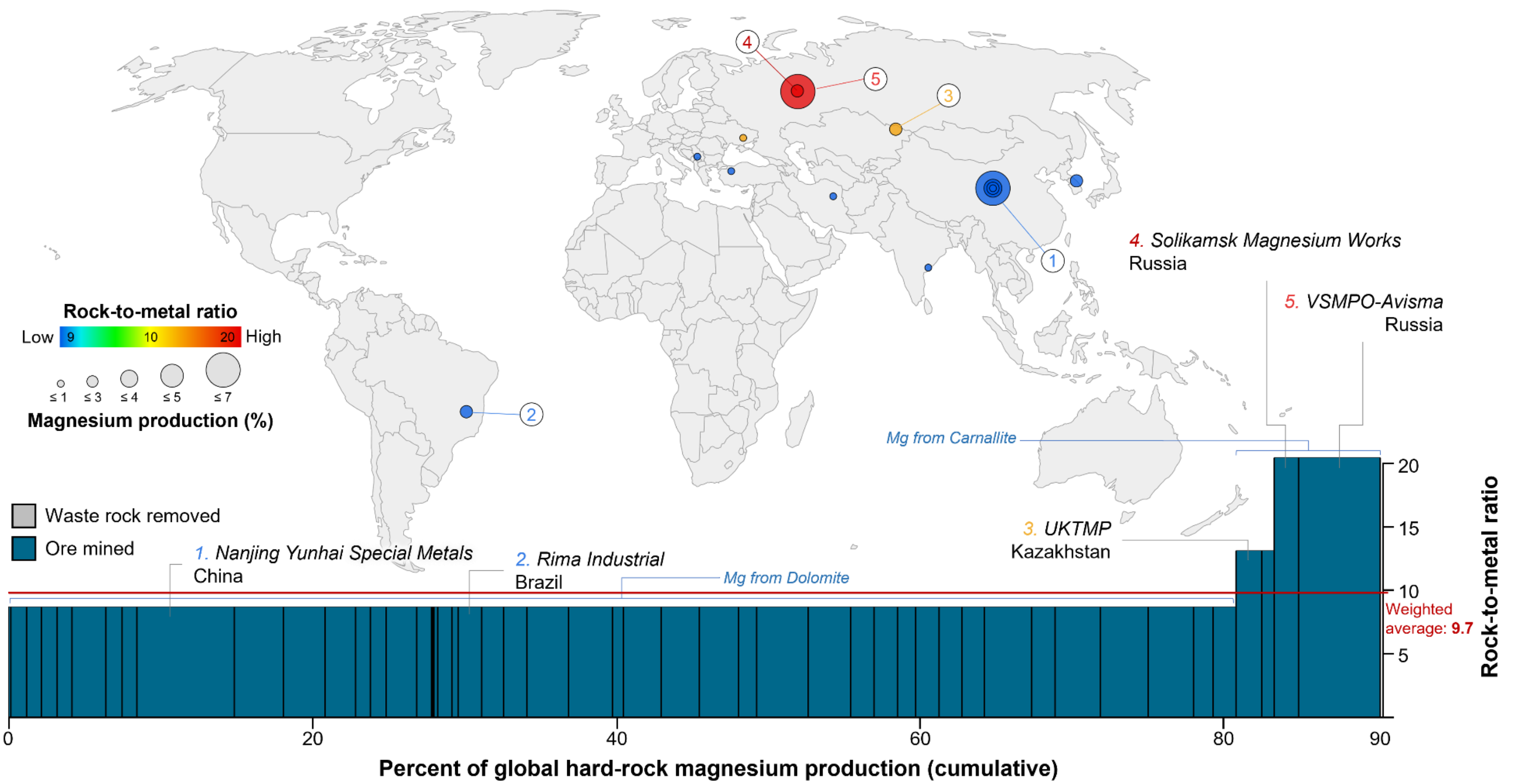

Figure S12. Map of the global distribution of hard-rock magnesium operations and bar plot of cumulative share of total global hard-rock magnesium production. Each individual operation is plotted as a single circle on the map and single bar on the plot. The colors of the circles indicate individual rock-to-metal ratios (RMRs), which range from a low of 8.7 to a high of 2.0×10^1 and yield a global RMR of 9.7 (n = 50). The sizes of the circles are proportional to an operation's share (in percent) of total global magnesium production, which range from a low of 0.06% to a high of 6.4% for a total global coverage of 90% of 2018 global magnesium hard-rock production (excluding brine) reported by the U.S. Geological Survey.[16] Operations are ordered from lowest to highest RMR on the bar plot.

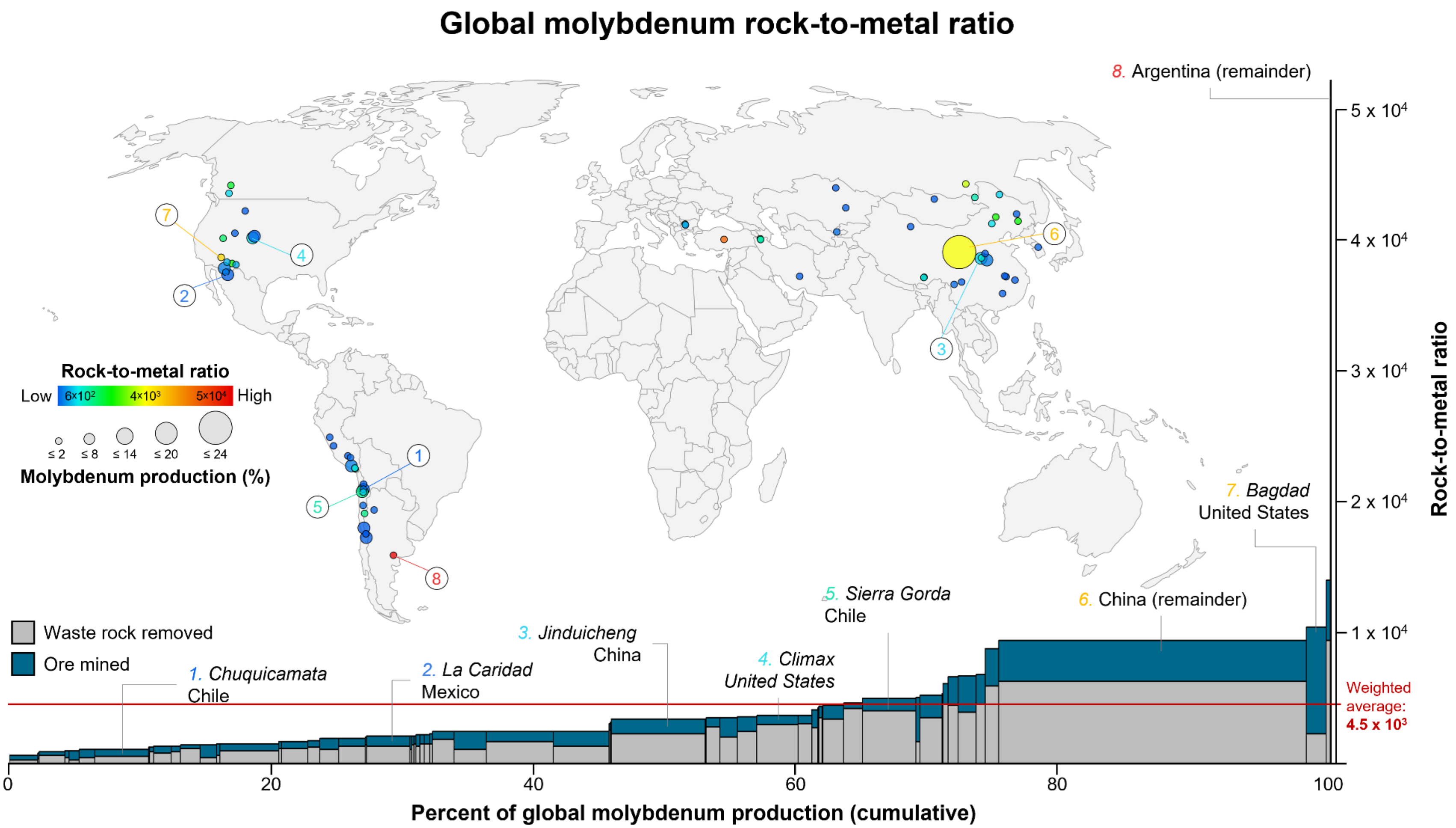

Figure S13. Map of the global distribution of molybdenum operations and bar plot of cumulative share of total global molybdenum production. Each individual operation is plotted as a single circle on the map and single bar on the plot. The colors of the circles indicate individual rock-to-metal ratios (RMRs), which range from a low of 5.8 × 10^2 to a high of 5.2 × 10^4 and yield a global RMR of 4.5 × 10^3 (n = 67). The sizes of the circles are proportional to an operation's share (in percent) of total global molybdenum production, which range from a low of 0.002% to a high of 23% for a total global coverage of 100% of 2018 global molybdenum production reported by the U.S. Geological Survey.[16] Operations are ordered from lowest to highest RMR on the bar plot.

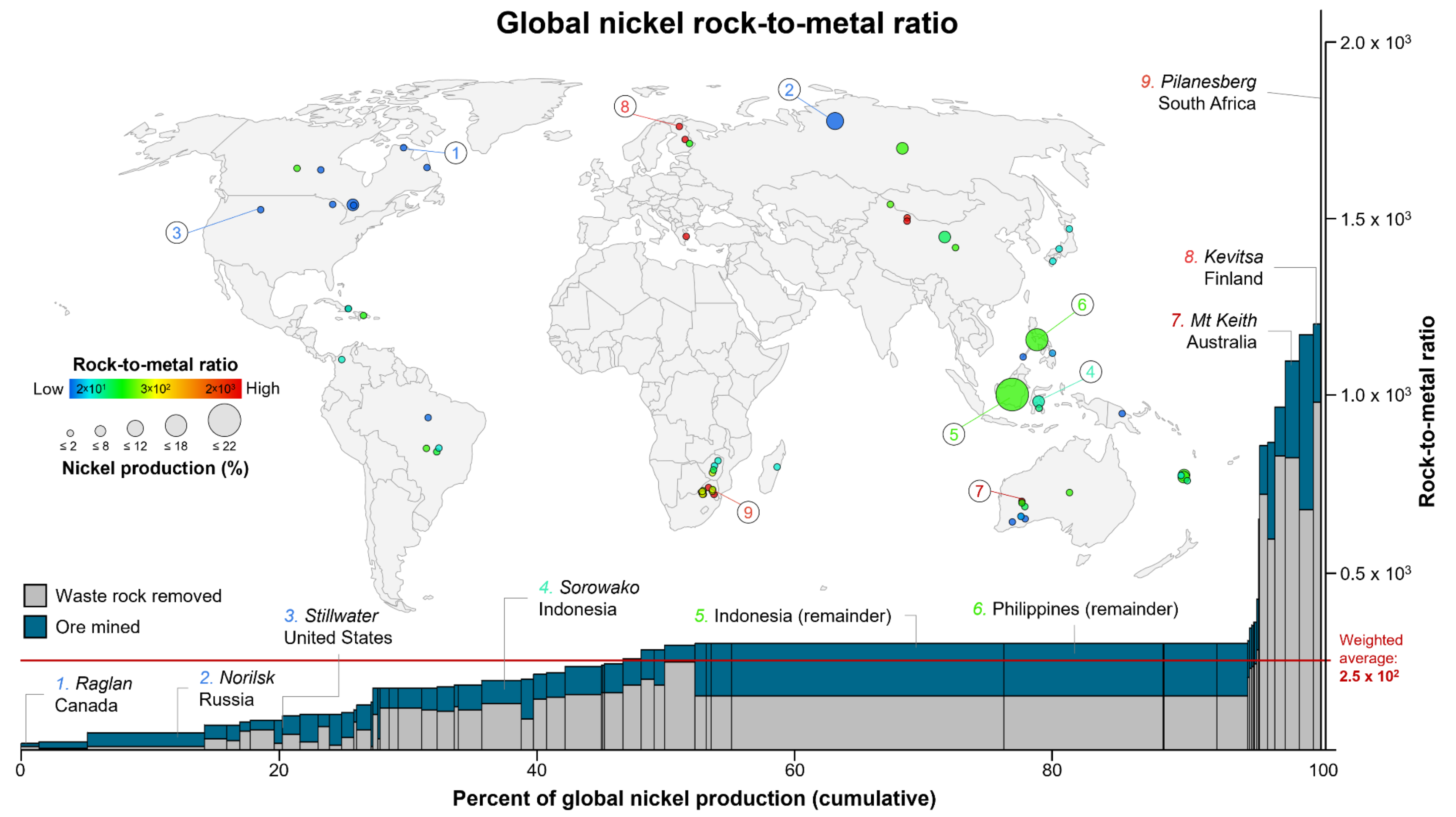

Figure S14. Map of the global distribution of nickel operations and bar plot of cumulative share of total global nickel production. Each individual operation is plotted as a single circle on the map and single bar on the plot. The colors of the circles indicate individual rock-to-metal ratios (RMRs), which range from a low of 1.5×10^1 to a high of 2.1×10^3 and yield a global RMR of 2.5×10^2 ($n = 69$). The sizes of the circles are proportional to an operation's share (in percent) of total global nickel production, which range from a low of 0.01% to a high of 21% for a total global coverage of 100% of 2018 global nickel production reported by the U.S. Geological Survey.[16] Operations are ordered from lowest to highest RMR on the bar plot.

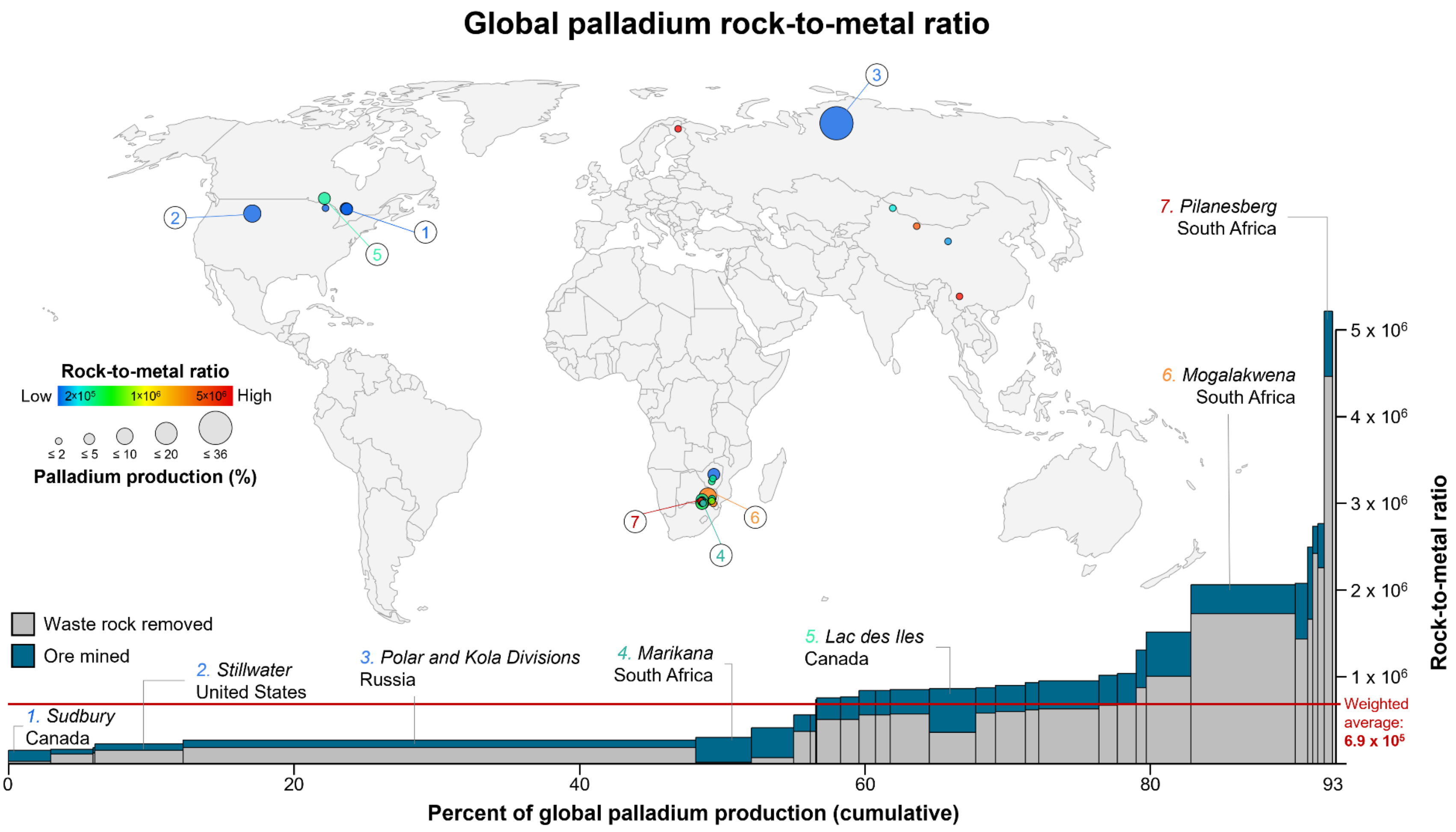

Figure S15. Map of the global distribution of palladium operations and bar plot of cumulative share of total global palladium production. Each individual operation is plotted as a single circle on the map and single bar on the plot. The colors of the circles indicate individual rock-to-metal ratios (RMRs), which range from a low of 1.6×10^5 to a high of 5.2×10^6 and yield a global RMR of 6.9×10^5 ($n = 32$). The sizes of the circles are proportional to an operation's share (in percent) of total global palladium production, which range from a low of 0.006% to a high of 36% for a total global coverage of 93% of 2018 global palladium production reported by the U.S. Geological Survey.[16] Operations are ordered from lowest to highest RMR on the bar plot.

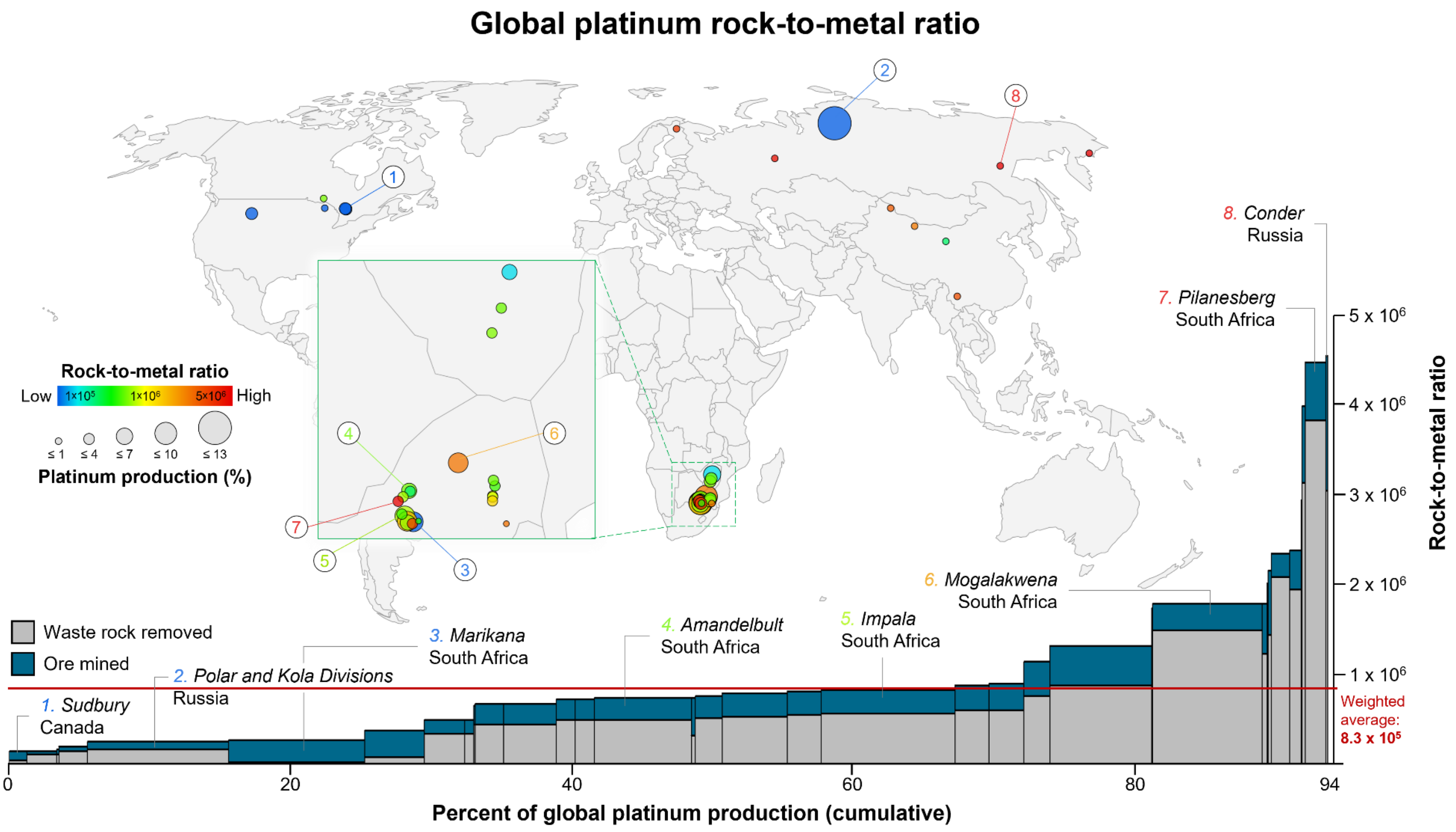

Figure S16. Map of the global distribution of platinum operations and bar plot of cumulative share of total global platinum production. Each individual operation is plotted as a single circle on the map and single bar on the plot. The colors of the circles indicate individual rock-to-metal ratios (RMRs), which range from a low of 1.3×10^5 to a high of 4.5×10^6 and yield a global RMR of 8.3×10^5 ($n = 35$). The sizes of the circles are proportional to an operation's share (in percent) of total global platinum production, which range from a low of 0.03% to a high of 10% for a total global coverage of 94% of 2018 global platinum production reported by the U.S. Geological Survey.[16] Operations are ordered from lowest to highest RMR on the bar plot.

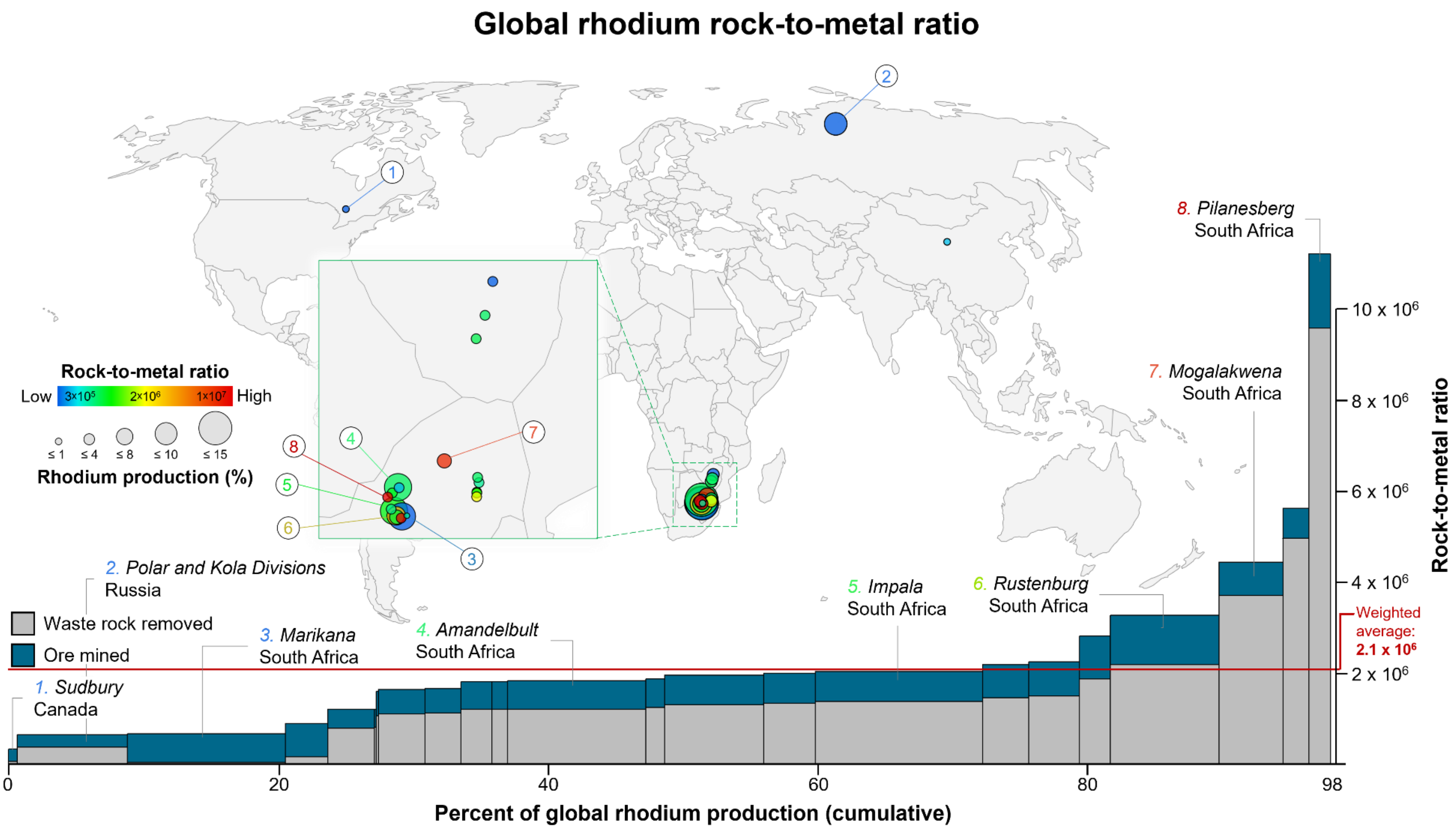

Figure S17. Map of the global distribution of rhodium operations and bar plot of cumulative share of total global rhodium production. Each individual operation is plotted as a single circle on the map and single bar on the plot. The colors of the circles indicate individual rock-to-metal ratios (RMRs), which range from a low of 3.3×10^5 to a high of 1.1×10^7 and yield a global RMR of 2.1×10^6 ($n = 23$). The sizes of the circles are proportional to an operation's share (in percent) of total global rhodium production, which range from a low of 0.085% to a high of 12% for a total global coverage of 98% of 2018 global rhodium production reported by the U.S. Geological Survey.[17] Operations are ordered from lowest to highest RMR on the bar plot.

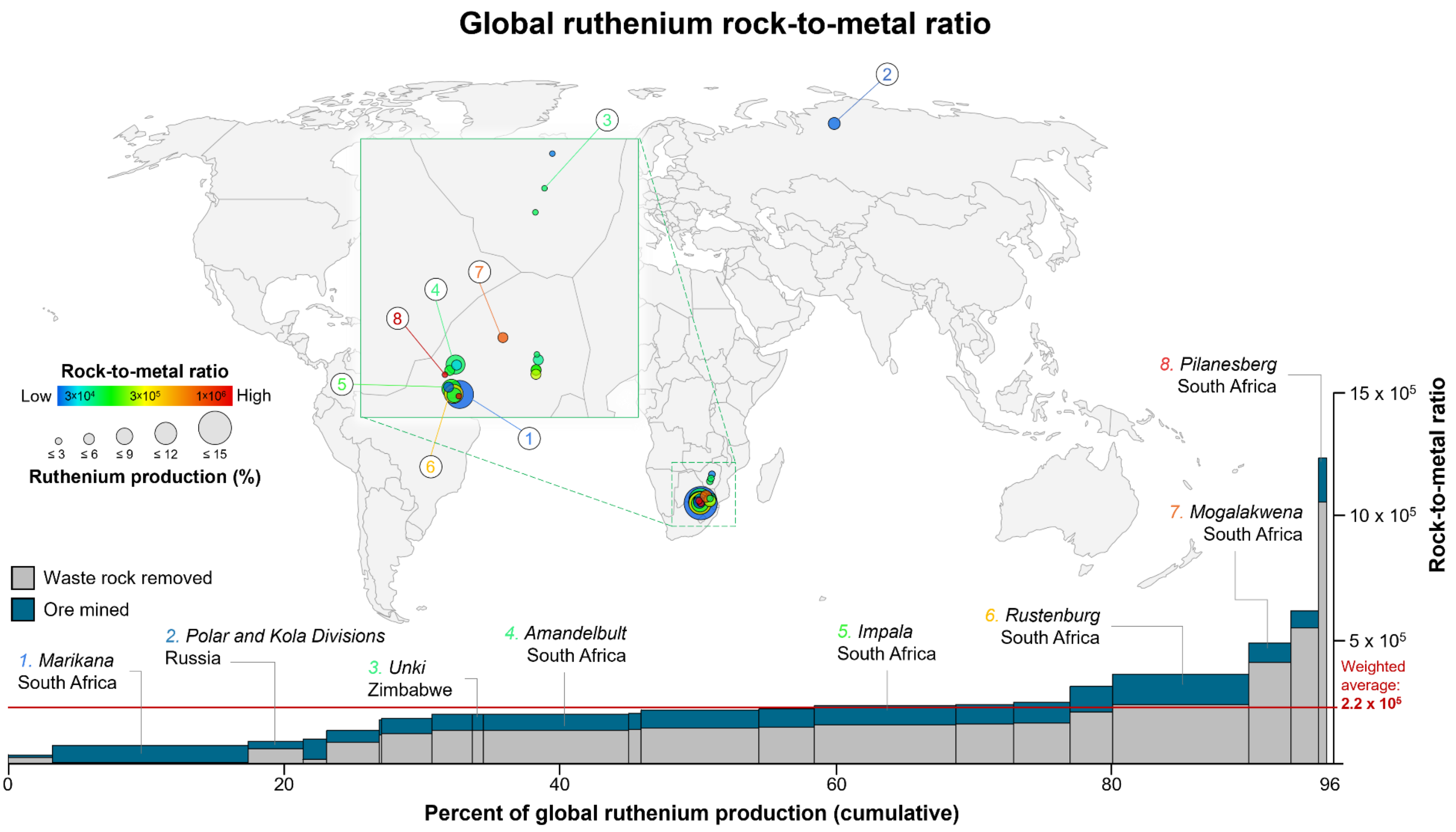

Figure S18. Map of the global distribution of ruthenium operations and bar plot of cumulative share of total global ruthenium production. Each individual operation is plotted as a single circle on the map and single bar on the plot. The colors of the circles indicate individual rock-to-metal ratios (RMRs), which range from a low of 3.4×10^4 to a high of 1.2×10^6 and yield a global RMR of 2.2×10^5 ($n = 21$). The sizes of the circles are proportional to an operation's share (in percent) of total global ruthenium production, which range from a low of 0.2% to a high of 14% for a total global coverage of 96% of 2018 global ruthenium production reported by the U.S. Geological Survey.[16] Operations are ordered from lowest to highest RMR on the bar plot.

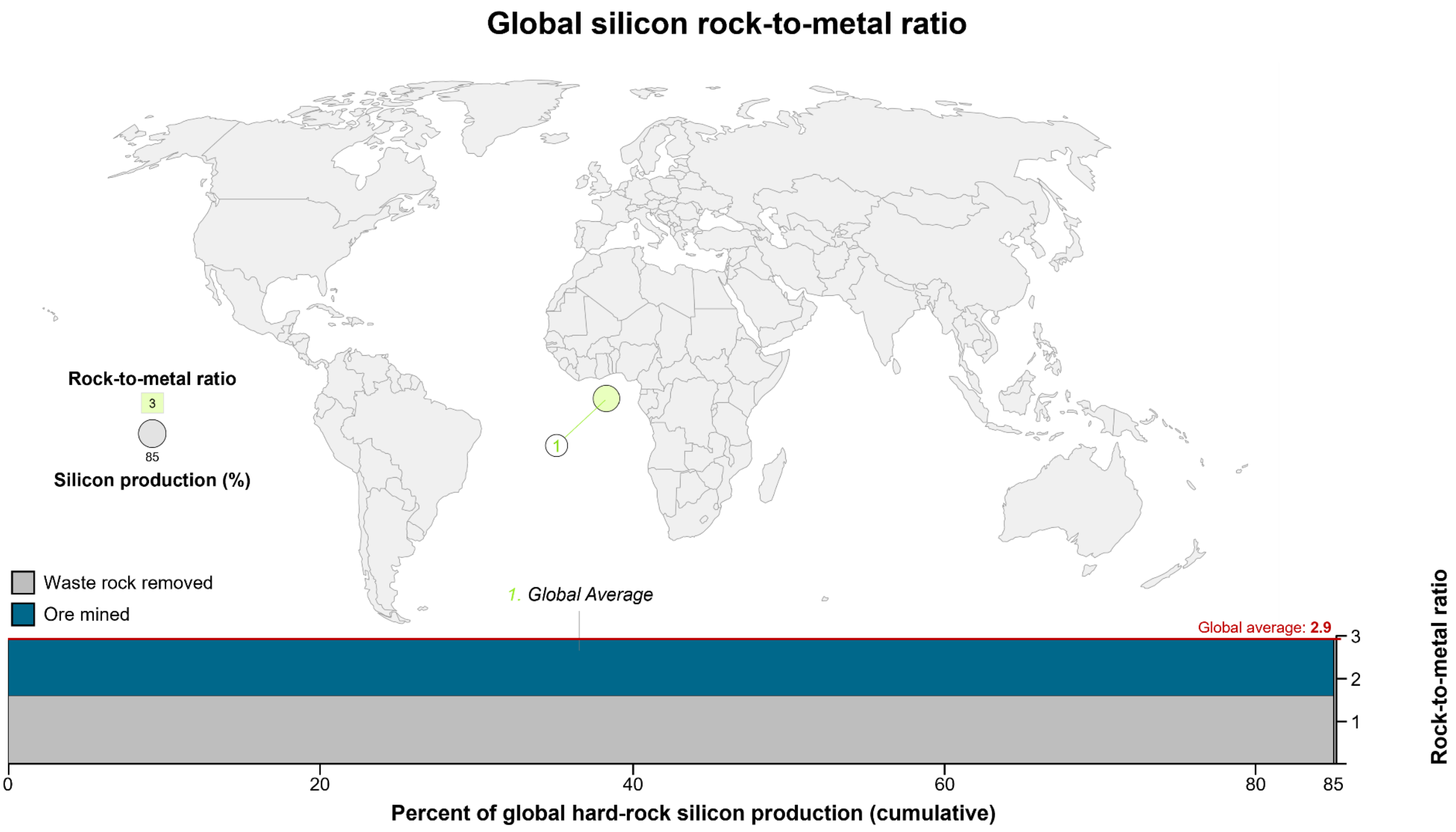

Figure S19. Map of the global distribution of silicon operations and bar plot of cumulative share of total global silicon production. Due to lack of data availability, the rock-to-metal ratio for silicon was calculated as a single global average at 2.9, representing an estimated 85% of 2018 global silicon production reported by the U.S. Geological Survey.[16]

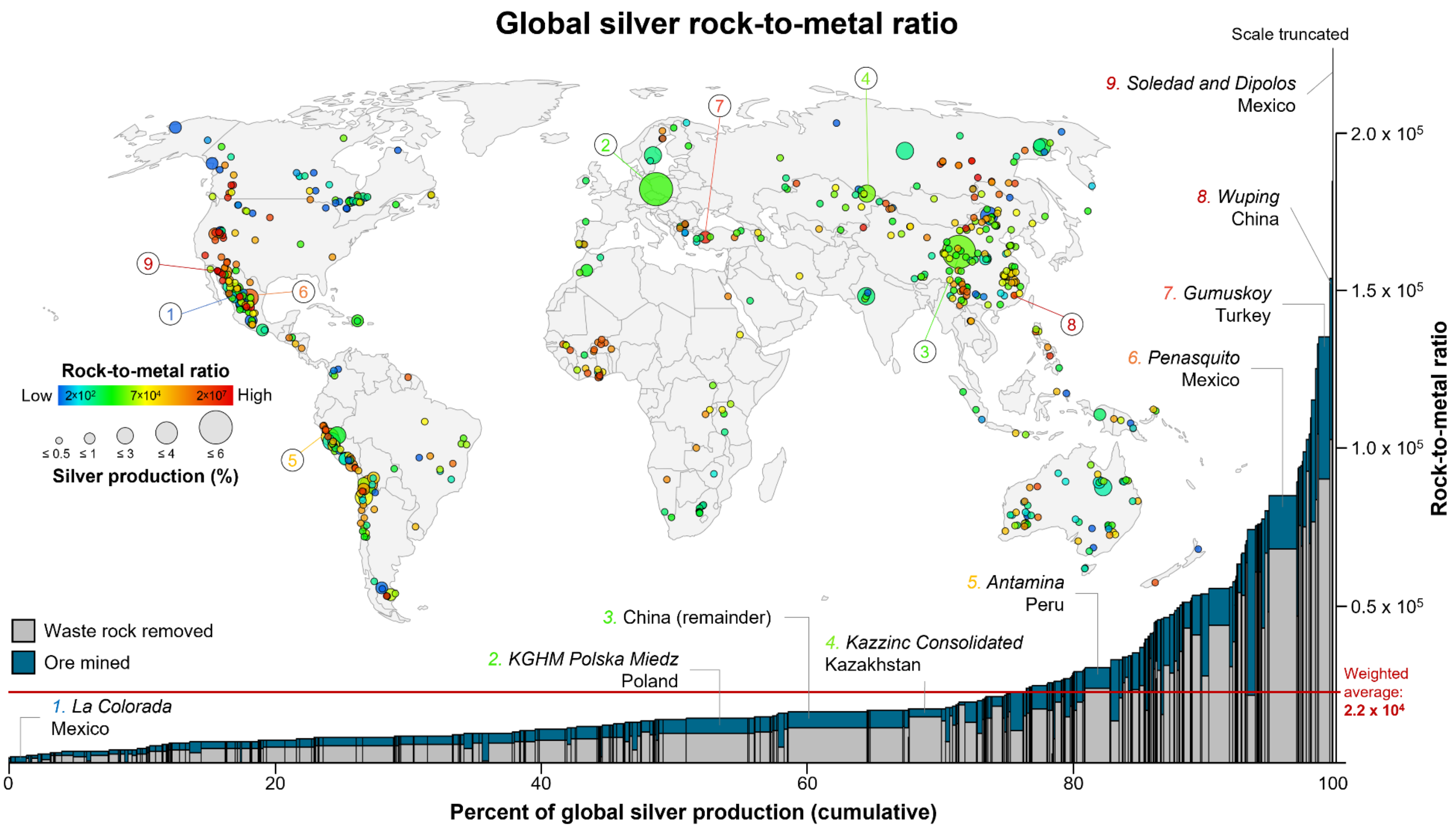

Figure S20. Map of the global distribution of silver operations and bar plot of cumulative share of total global silver production. Each individual operation is plotted as a single circle on the map and single bar on the plot. The colors of the circles indicate individual rock-to-metal ratios (RMRs), which range from a low of 1.6×10^3 to a high of 2.1×10^7 and yield a global RMR of 2.2×10^4 ($n = 627$). The sizes of the circles are proportional to an operation's share (in percent) of total global silver production, which range from a low of <0.001% to a high of 5.9% for a total global coverage of 100% of 2018 global silver production reported by the U.S. Geological Survey.[16] Operations are ordered from lowest to highest RMR on the bar plot.

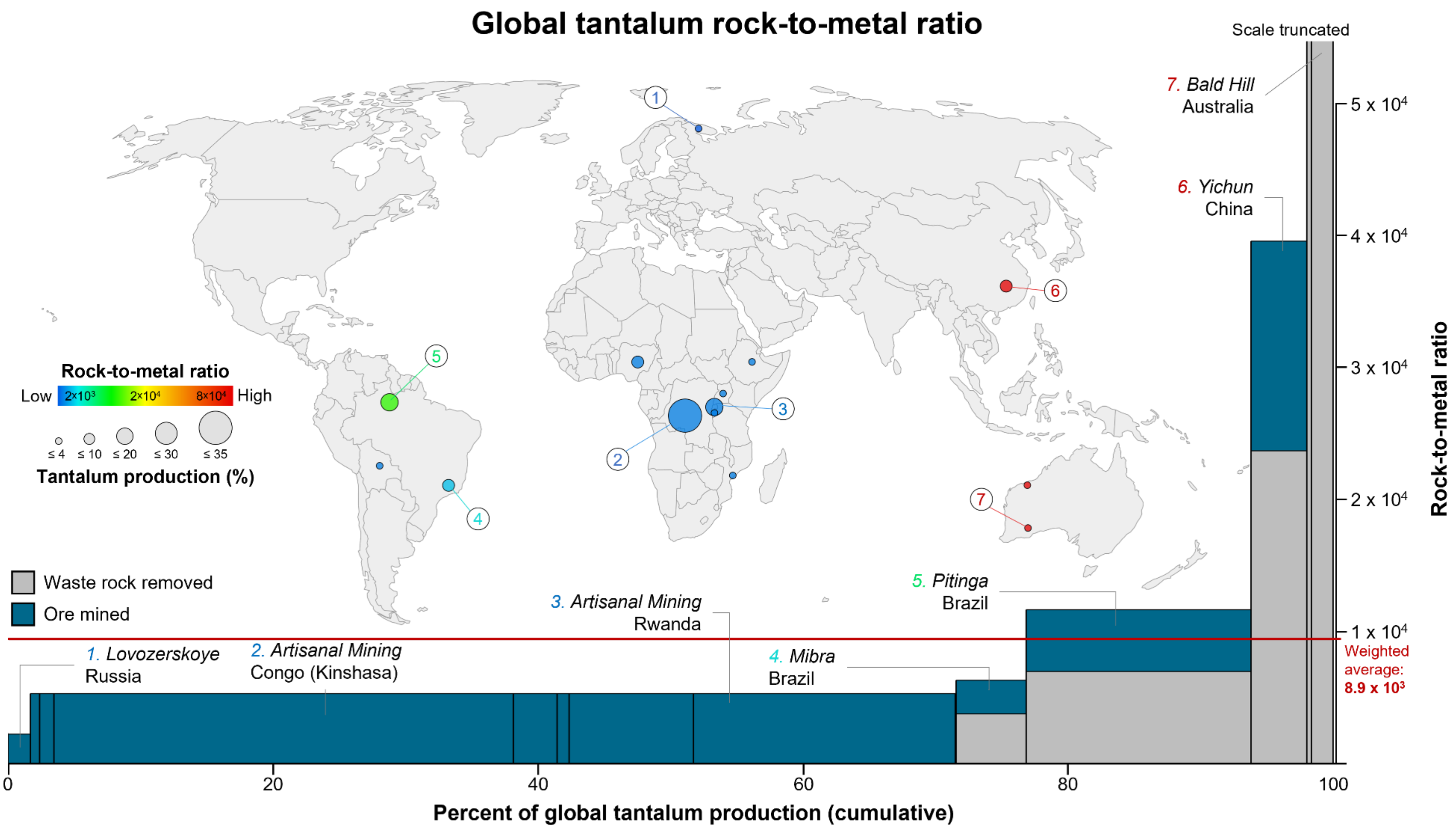

Figure S21. Map of the global distribution of tantalum operations and bar plot of cumulative share of total global tantalum production. Each individual operation is plotted as a single circle on the map and single bar on the plot. The colors of the circles indicate individual rock-to-metal ratios (RMRs), which range from a low of 2.1 × 10^3 to a high of 8.3 × 10^4 and yield a global RMR of 8.9 × 10^3 (n = 14). The sizes of the circles are proportional to an operation's share (in percent) of total global tantalum production, which range from a low of 0.14% to a high of 35% for a total global coverage of 100% of 2018 global tantalum production revised from that reported by the U.S. Geological Survey.[16] Operations are ordered from lowest to highest RMR on the bar plot.

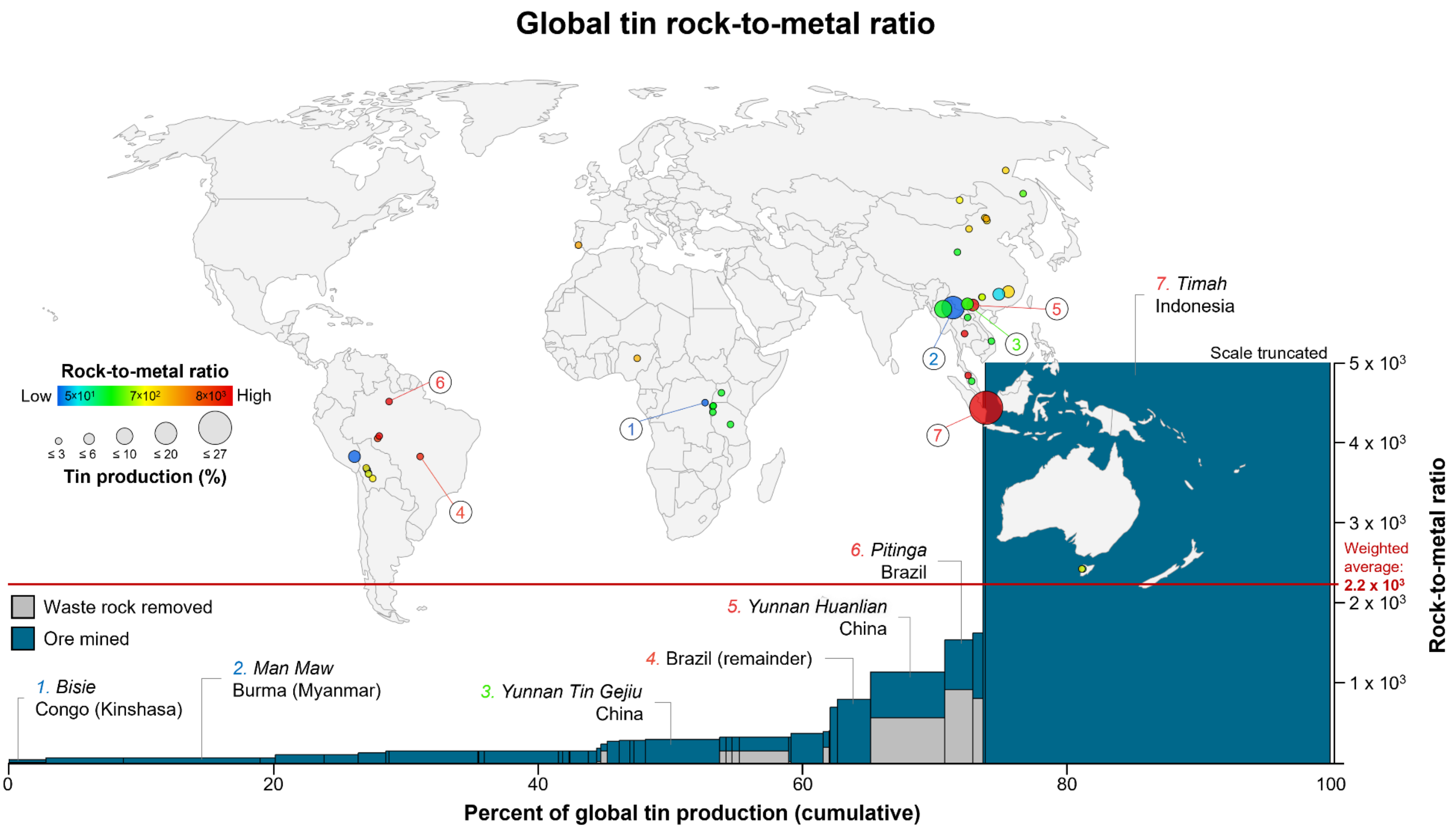

Figure S22. Map of the global distribution of tin operations and bar plot of cumulative share of total global tin production. Each individual operation is plotted as a single circle on the map and single bar on the plot. The colors of the circles indicate individual rock-to-metal ratios (RMRs), which range from a low of 46 to a high of 7.6 × 10³ and yield a global RMR of 2.2 × 10³ (n = 43). The sizes of the circles are proportional to an operation's share (in percent) of total global tin production, which range from a low of 0.001% to a high of 26% for a total global coverage of 100% of 2018 global tin production revised from that reported by the U.S. Geological Survey.[16] Operations are ordered from lowest to highest RMR on the bar plot.

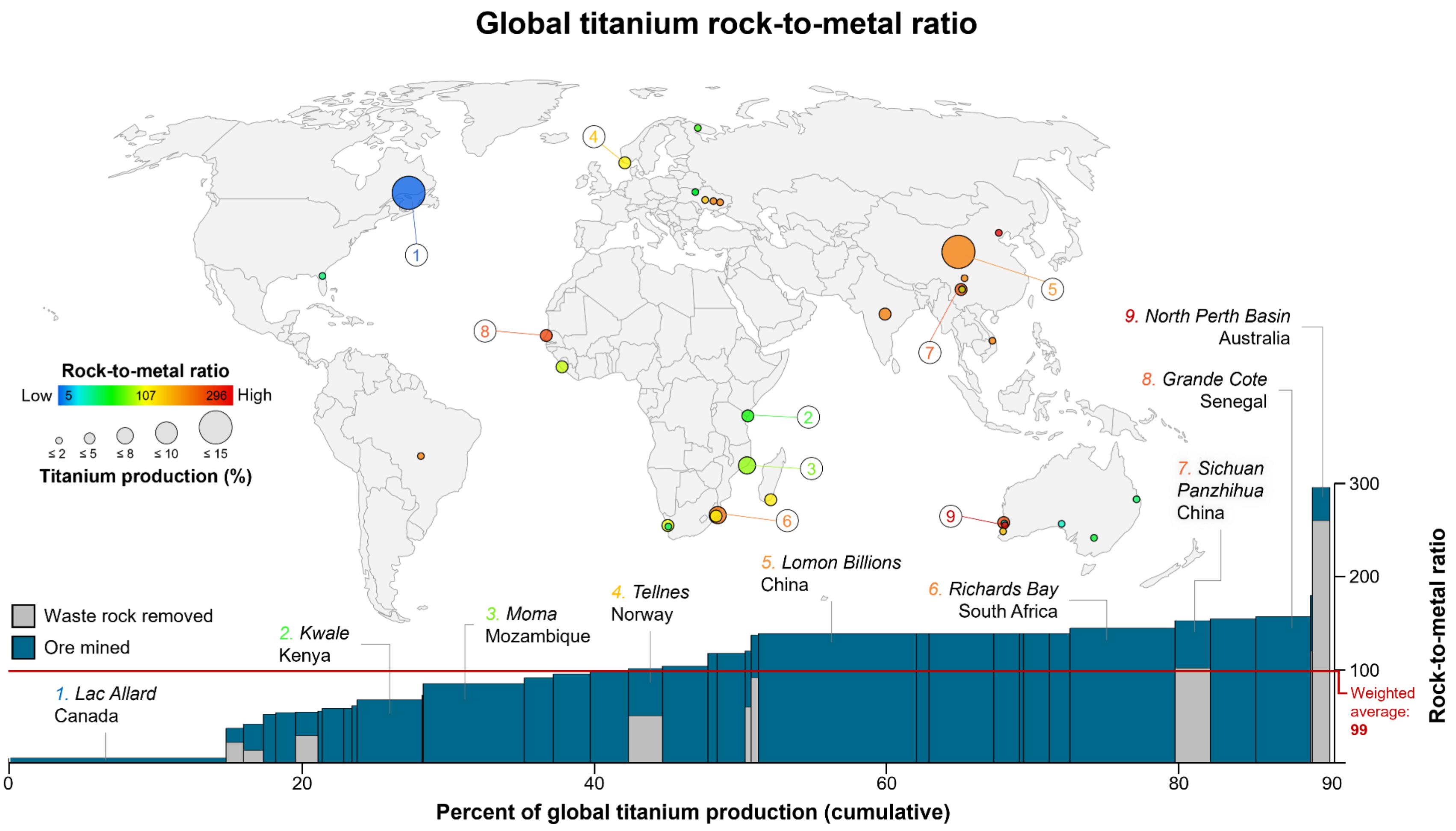

Figure S23. Map of the global distribution of titanium operations and bar plot of cumulative share of total global titanium production. Each individual operation is plotted as a single circle on the map and single bar on the plot. The colors of the circles indicate individual rock-to-metal ratios (RMRs), which range from a low of 5.5 to a high of 3.0 × 10^2 and yield a global RMR of 9.9 × 10^1 (n = 35). The sizes of the circles are proportional to an operation's share (in percent) of total global titanium production, which range from a low of 0.049% to a high of 15% for a total global coverage of 90% of 2018 global titanium production reported by the U.S. Geological Survey.[16] Operations are ordered from lowest to highest RMR on the bar plot.

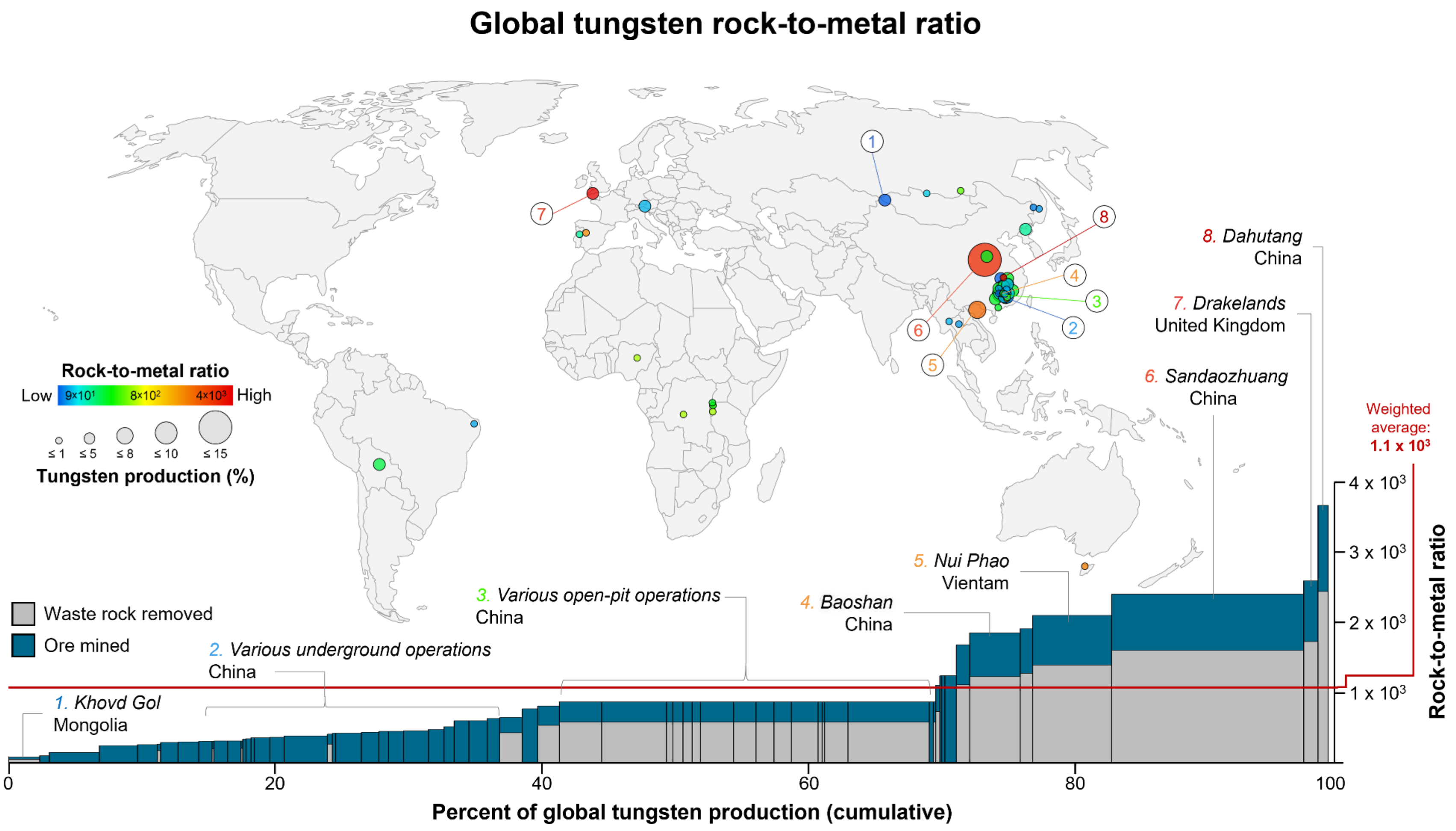

Figure S24. Map of the global distribution of tungsten operations and bar plot of cumulative share of total global tungsten production. Each individual operation is plotted as a single circle on the map and single bar on the plot. The colors of the circles indicate individual rock-to-metal ratios (RMRs), which range from a low of 9.3×10^1 to a high of 3.7×10^3 and yield a global RMR of 1.1×10^3 ($n = 64$). The sizes of the circles are proportional to an operation's share (in percent) of total global tungsten production, which range from a low of 0.025% to a high of 14% for a total global coverage of 99% of 2018 global tungsten production reported by the U.S. Geological Survey.[16] Operations are ordered from lowest to highest RMR on the bar plot.

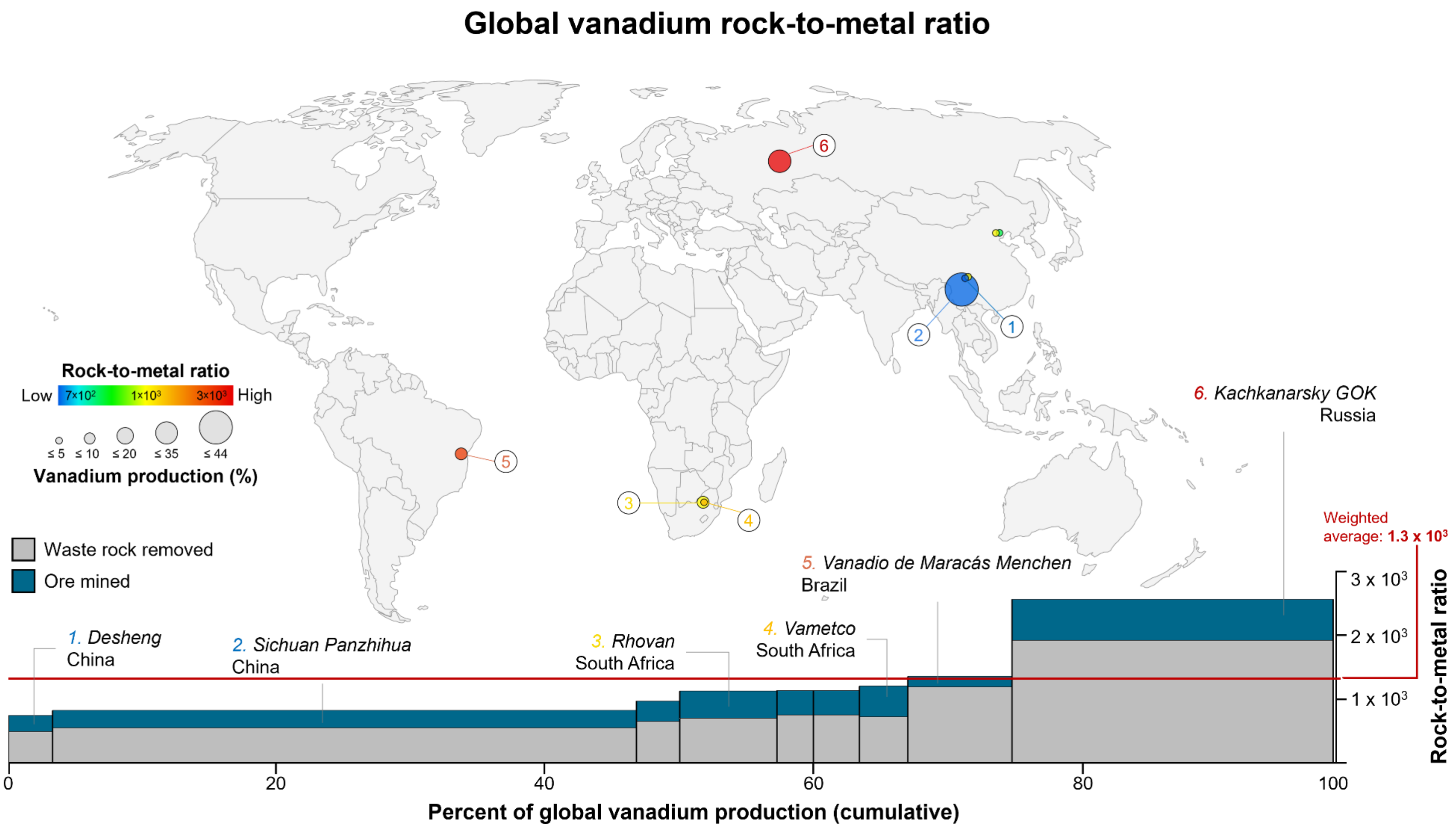

Figure S25. Map of the global distribution of vanadium operations and bar plot of cumulative share of total global vanadium production. Each individual operation is plotted as a single circle on the map and single bar on the plot. The colors of the circles indicate individual rock-to-metal ratios (RMRs), which range from a low of 7.5 × 10^2 to a high of 2.6 × 10^3 and yield a global RMR of 1.3 × 10^3 (n = 9). The sizes of the circles are proportional to an operation's share (in percent) of total global vanadium production, which range from a low of 2.7 % to a high of 43% for a total global coverage of 99% of 2018 global vanadium production reported by the U.S. Geological Survey.[16] Operations are ordered from lowest to highest RMR on the bar plot.

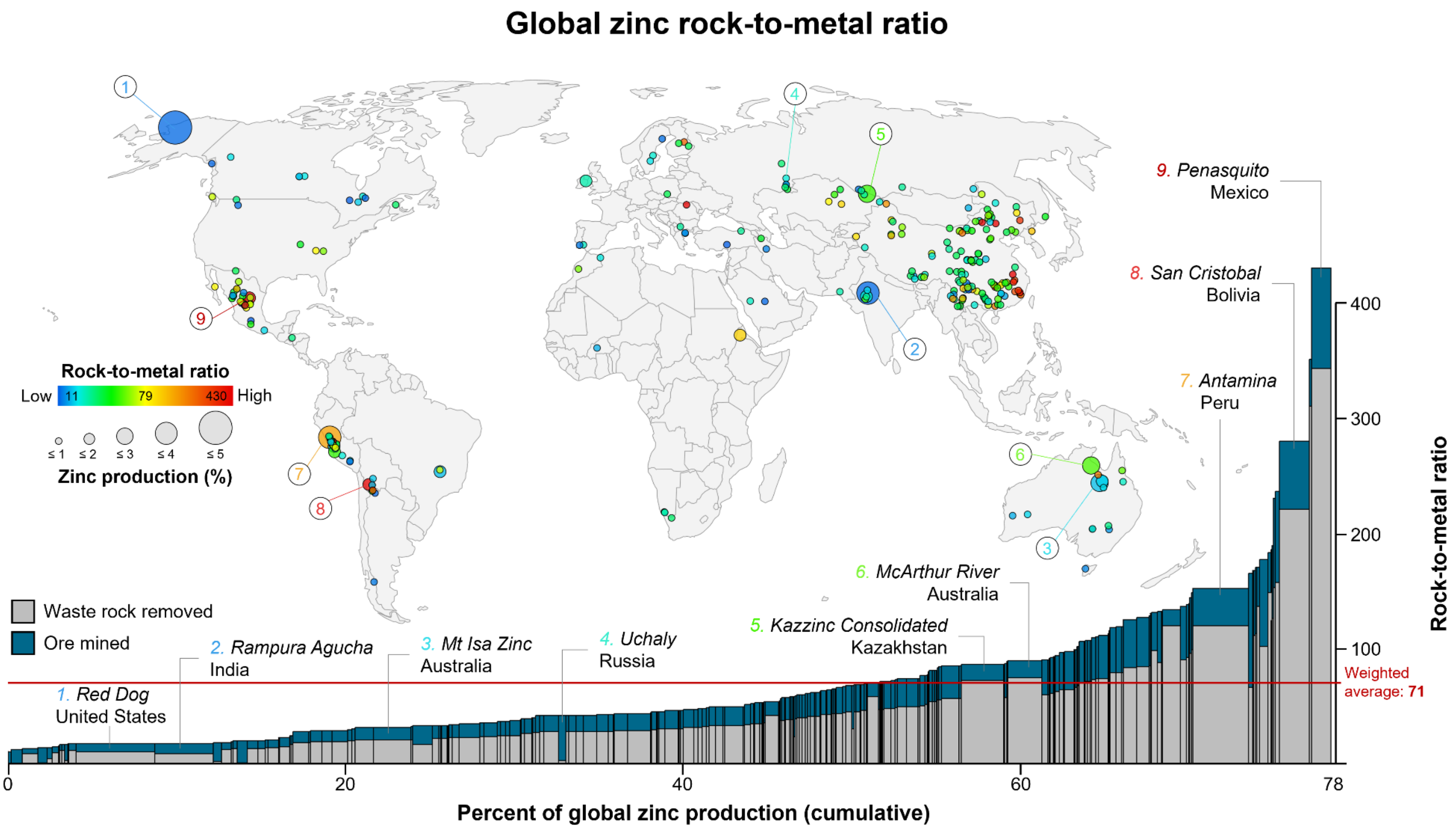

Figure S26. Map of the global distribution of zinc operations and bar plot of cumulative share of total global zinc production. Each individual operation is plotted as a single circle on the map and single bar on the plot. The colors of the circles indicate individual rock-to-metal ratios (RMRs), which range from a low of 1.1×10^1 to a high of 4.3×10^2 and yield a global RMR of 7.1×10^2 (n = 284). The sizes of the circles are proportional to an operation's share (in percent) of total global zinc production, which range from a low of <0.001% to a high of 4.7% for a total global coverage of 78% of 2018 global zinc production reported by the U.S. Geological Survey.[16] Operations are ordered from lowest to highest RMR on the bar plot.

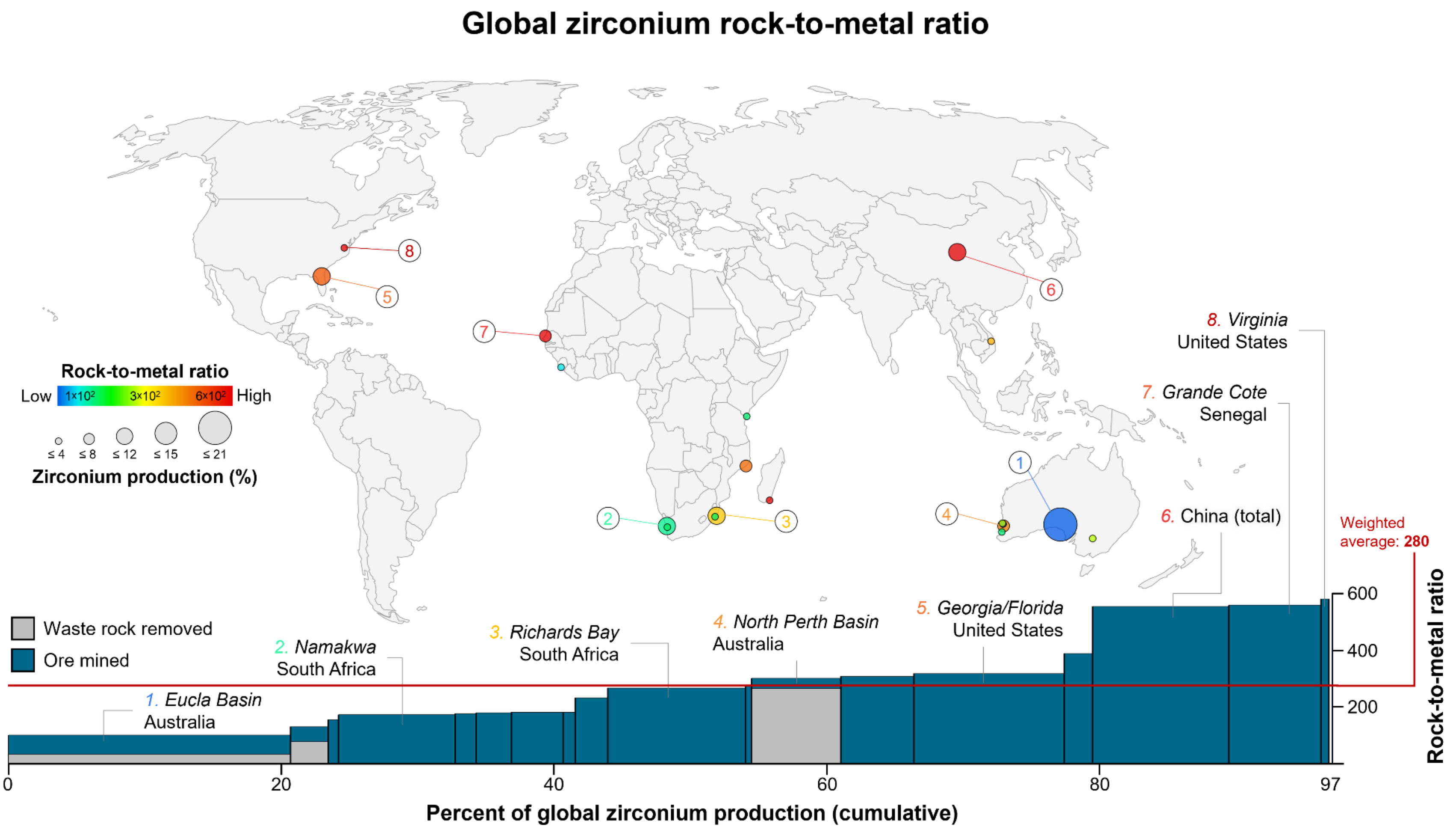

Figure S27. Map of the global distribution of zirconium operations and bar plot of cumulative share of total global zirconium production. Each individual operation is plotted as a single circle on the map and single bar on the plot. The colors of the circles indicate individual rock-to-metal ratios (RMRs), which range from a low of 1.0 × 10^2 to a high of 5.8 × 10^2 and yield a global RMR of 2.8 × 10^2 (n = 19). The sizes of the circles are proportional to an operation's share (in percent) of total global zirconium production, which range from a low of 0.007% to a high of 21% for a total global coverage of 97% of 2018 global zirconium production reported by the U.S. Geological Survey.[16] Operations are ordered from lowest to highest RMR on the bar plot.

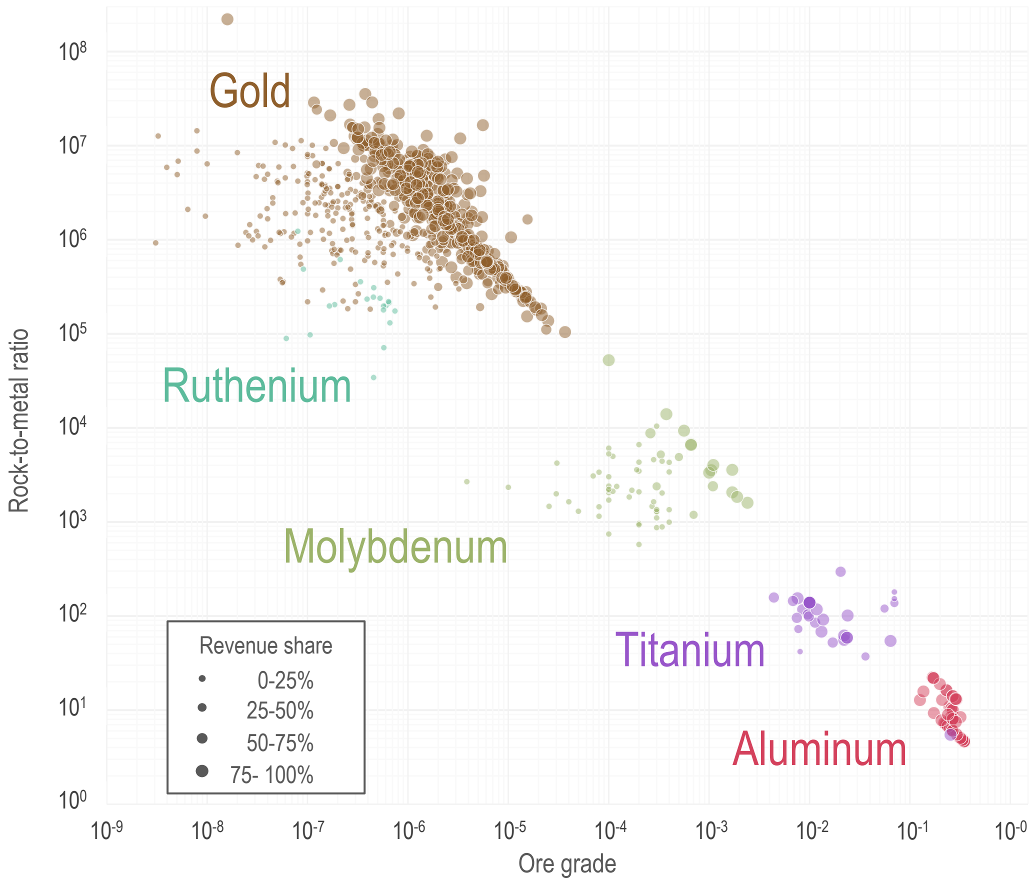

Figure S28. Rock-to-metal ratio (vertical axis) versus ore grade (horizontal axis) for aluminum, gold, molybdenum, ruthenium, and titanium by individual operation. Axes are on a $\log_{10}$-$\log_{10}$ scale. Colors correspond to different commodities. Marker size corresponds to revenue share (economic allocation) attributable to the mineral commodity at the specific operation.

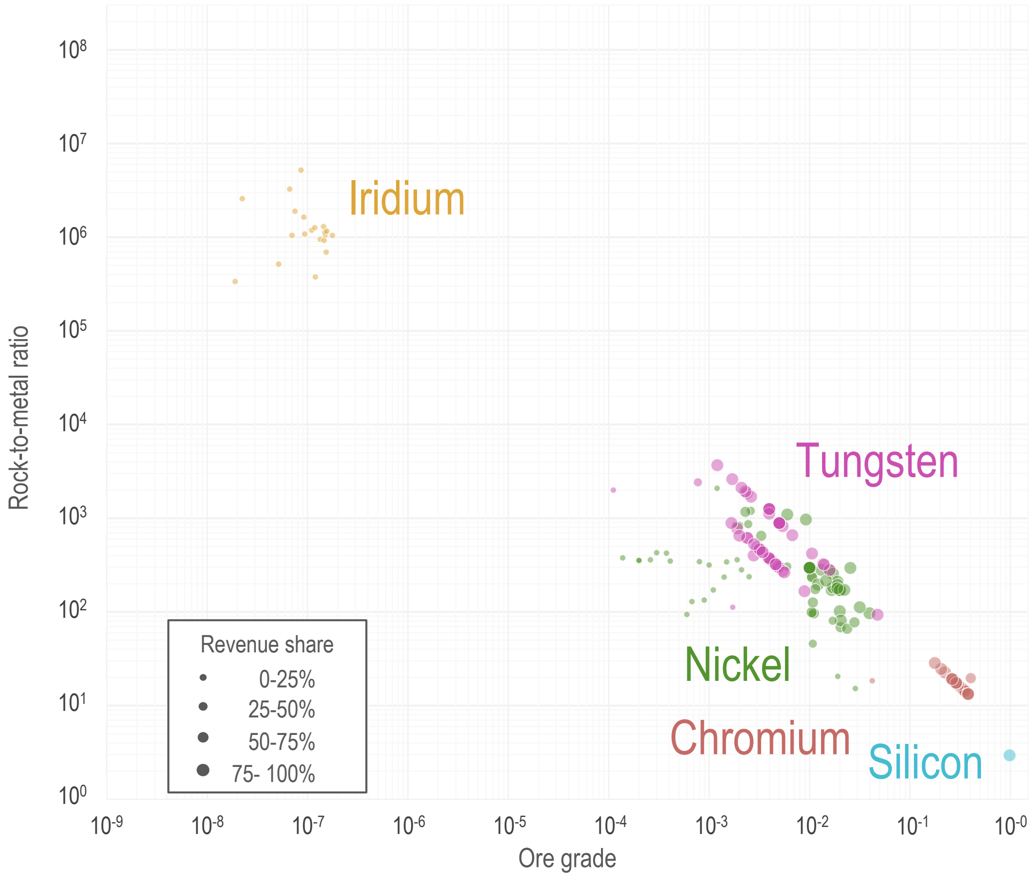

Figure S29. Rock-to-metal ratio (vertical axis) versus ore grade (horizontal axis) chromium, iridium, nickel, silicon, and tungsten by individual operation. Axes are on a log_{10}-log_{10} scale. Colors correspond to different commodities. Marker size corresponds to revenue share (economic allocation) attributable to the mineral commodity at the specific operation.

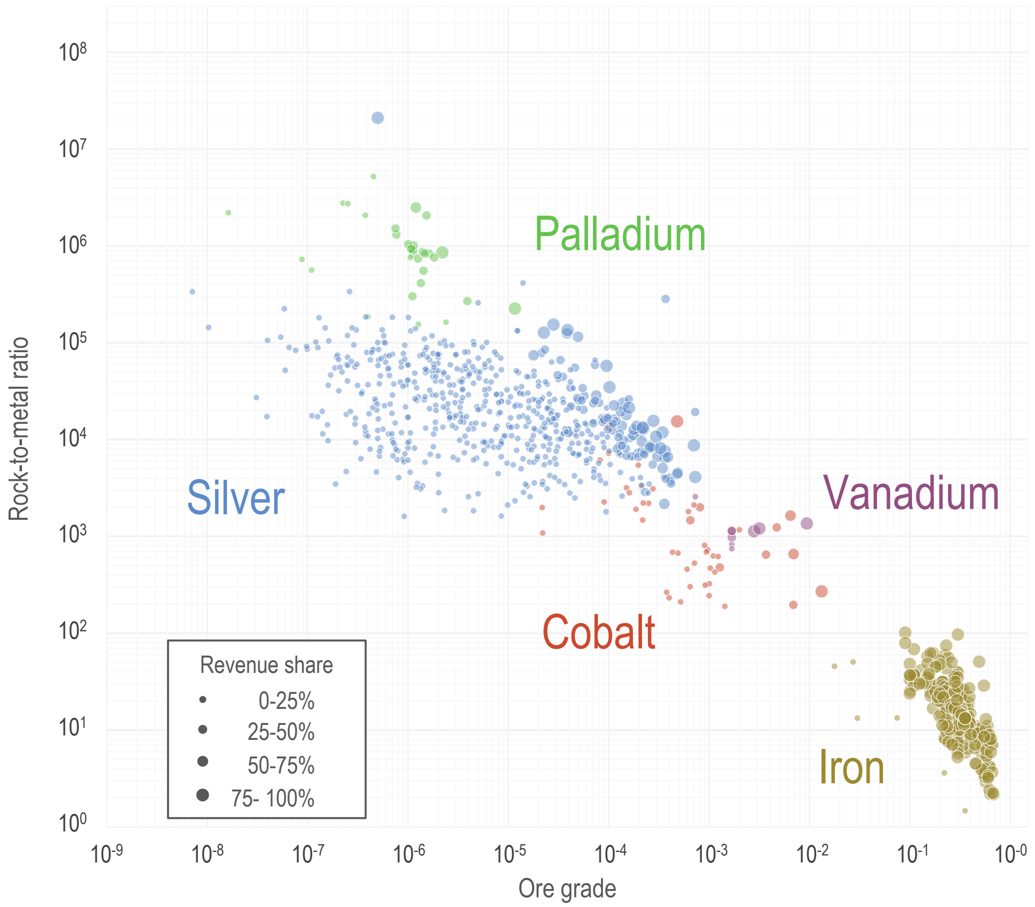

Figure S30. Rock-to-metal ratio (vertical axis) versus ore grade (horizontal axis) cobalt, iron, palladium, silver, and vanadium by individual operation. Axes are on a $\log_{10}$-$\log_{10}$ scale. Colors correspond to different commodities. Marker size corresponds to revenue share (economic allocation) attributable to the mineral commodity at the specific operation.

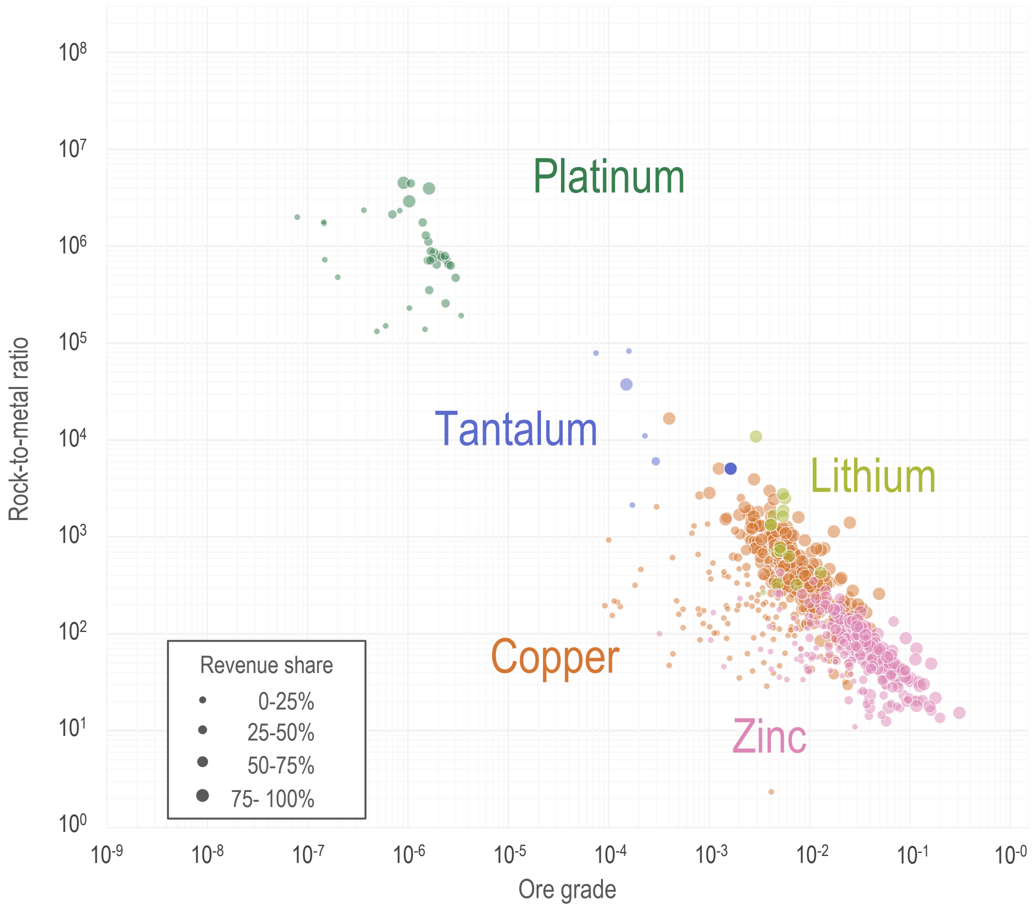

Figure S31. Rock-to-metal ratio (vertical axis) versus ore grade (horizontal axis) copper, lithium, platinum, tantalum, and zinc by individual operation. Axes are on a $\log_{10}$-$\log_{10}$ scale. Colors correspond to different commodities. Marker size corresponds to revenue share (economic allocation) attributable to the mineral commodity at the specific operation.

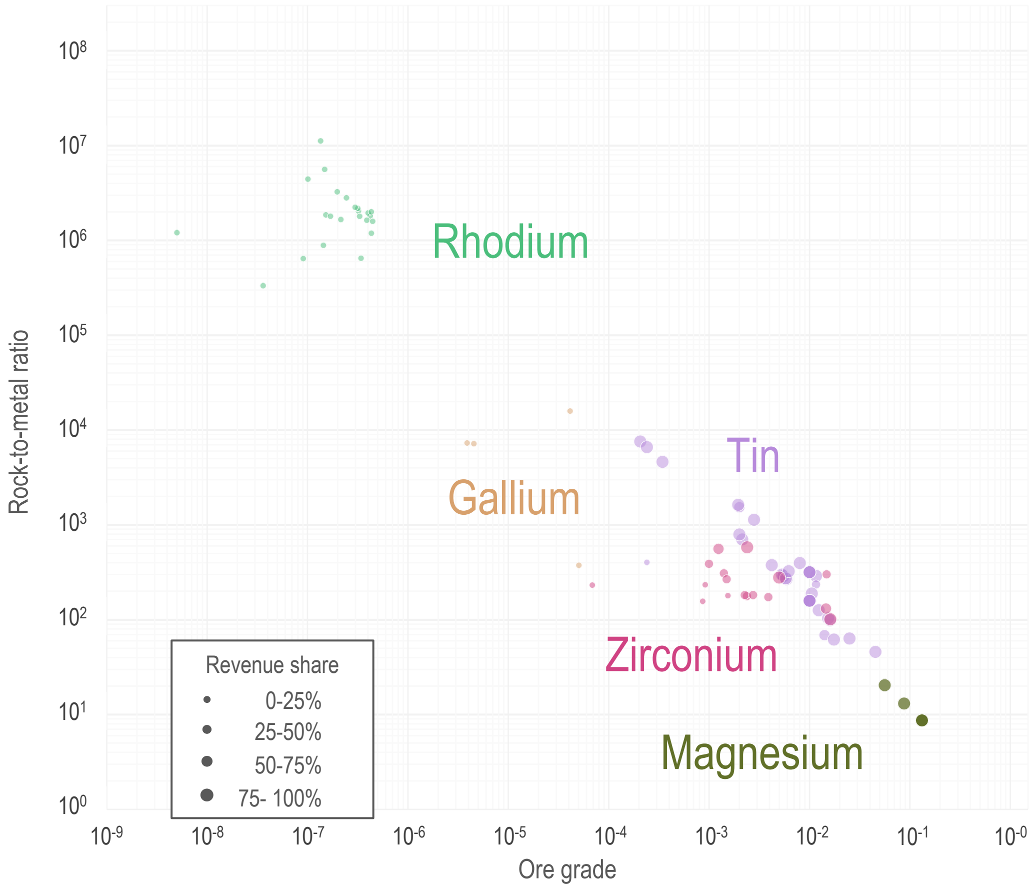

Figure S32. Rock-to-metal ratio (vertical axis) versus ore grade (horizontal axis) gallium, magnesium, rhodium, tin, and zirconium by individual operation. Axes are on a $\log_{10}$-$\log_{10}$ scale. Colors correspond to different commodities. Marker size corresponds to revenue share (economic allocation) attributable to the mineral commodity at the specific operation.

Factor analysis

Table S8. Contribution of each factor to the overall rock to metal ratio (RMR) expressed as a percentage.

Commodity	Percent contribution to rock to metal ratio (RMR)				
	Concentrator recovery rate	Ore grade	Refinery recovery rate	Revenue share	Waste to ore ratio
Aluminum	0.0%	28.0%	0.0%	0.0%	72.0%
Chromium	0.0%	95.6%	0.0%	1.8%	2.6%
Cobalt	1.8%	56.4%	0.0%	39.7%	2.0%
Copper	19.5%	48.0%	0.0%	26.3%	6.2%
Gallium	2.0%	13.8%	1.0%	68.6%	14.6%
Gold	12.4%	64.3%	0.0%	15.2%	8.1%
Iridium	48.6%	16.4%	0.0%	18.8%	16.3%
Iron	14.2%	42.3%	0.0%	0.2%	43.3%
Lithium	1.1%	47.3%	0.0%	1.2%	50.4%
Magnesium	0.0%	100.0%	0.0%	0.0%	0.0%
Molybdenum	33.6%	18.2%	0.0%	45.7%	2.4%
Nickel	14.5%	21.5%	0.0%	14.1%	49.9%
Palladium	33.3%	8.4%	0.0%	3.8%	54.5%
Platinum	11.5%	10.9%	0.0%	66.4%	11.2%
Rhodium	4.4%	6.0%	0.0%	7.9%	81.7%
Ruthenium	10.5%	20.2%	0.0%	6.6%	62.7%
Silicon	-	-	-	-	-
Silver	17.9%	13.8%	0.0%	68.1%	0.2%
Tantalum	1.0%	18.1%	0.1%	66.7%	14.1%
Tin	0.1%	97.8%	1.2%	0.2%	0.8%
Titanium	3.9%	34.5%	3.4%	20.8%	37.4%
Tungsten	4.4%	74.8%	0.0%	0.2%	20.6%
Vanadium	11.1%	23.9%	2.1%	58.8%	4.2%
Zinc	6.3%	52.1%	0.0%	30.9%	10.7%
Zirconium	3.2%	29.3%	0.0%	62.2%	5.3%
All	**4.0%**	**68.9%**	**4.9%**	**16.9%**	**5.4%**

Figure S33. Contribution of each factor to the overall rock to metal ratio (RMR) expressed as a percentage.

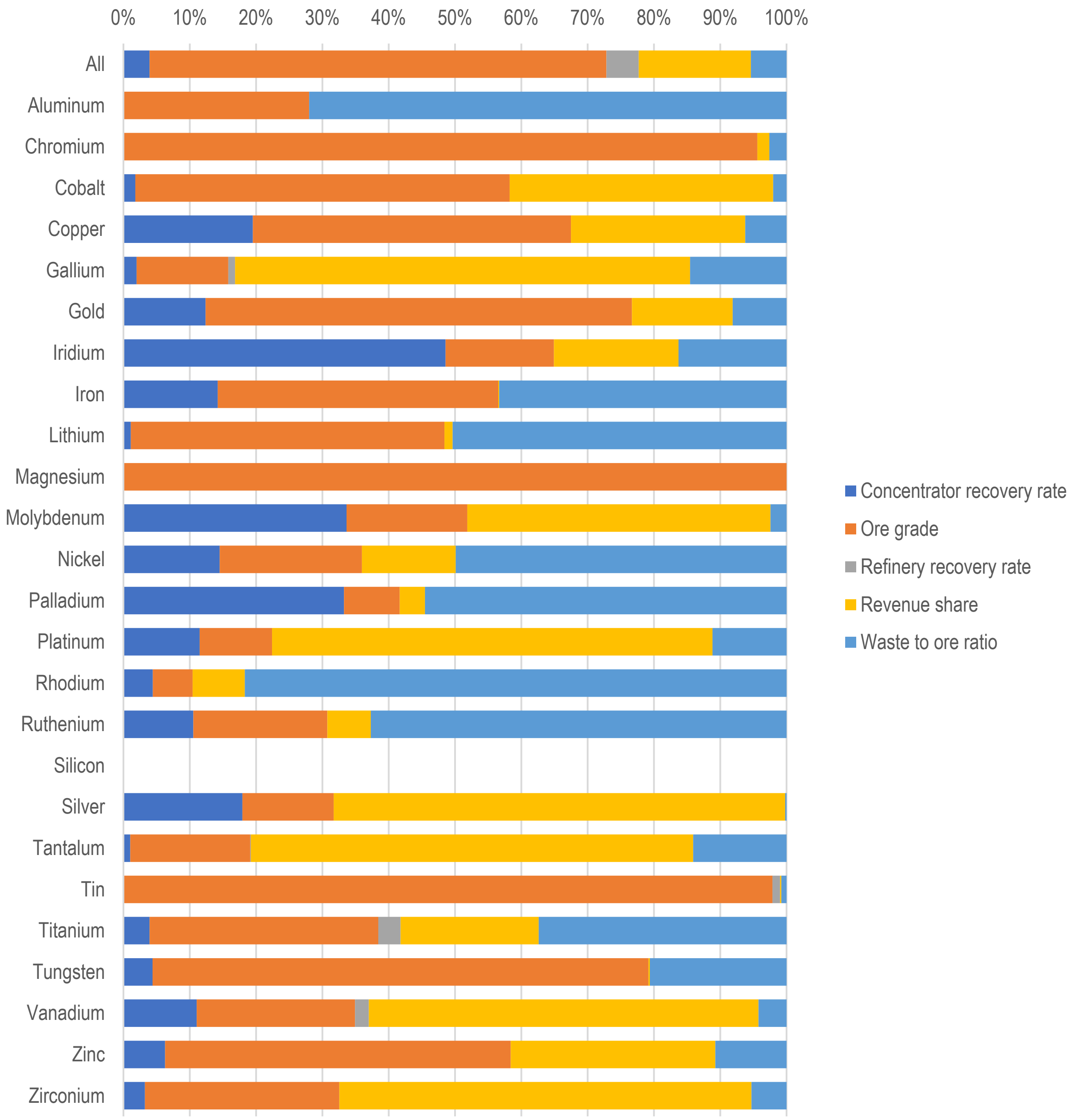

References

(1) Tabereaux, A. T.; Peterson, R. D. Aluminum Production. In *Treatise on Process Metallurgy*; Elsevier, 2014; pp 839–917.

(2) Frenzel, M.; Ketris, M. P.; Seifert, T.; Gutzmer, J. On the Current and Future Availability of Gallium. *Resour. Policy* **2016**, *47*, 38–50.

(3) Nassar, N. T. Global Stocks and Flows, Losses, and Recoveries of Platinum-Group Elements, Yale University, 2015.

(4) Sun, S. S. A Study of Kinetics and Mechanisms of Iron Ore Reduction in Ore/Coal Composites, McMaster University, 1997.

(5) Lasheen, T. A.; El-Ahmady, M. E.; Hassib, H. B.; Helal, A. S. Molybdenum Metallurgy Review: Hydrometallurgical Routes to Recovery of Molybdenum from Ores and Mineral Raw Materials. *Miner. Process. Extr. Metall. Rev.* **2015**, *36* (3), 145–173.

(6) Frischknecht, R.; Jungbluth, N.; Althaus, H.-J.; Doka, G.; Dones, R.; Heck, T.; Hellweg, S.; Hischier, R.; Nemecek, T.; Rebitzer, G.; Spielmann, M. The Ecoinvent Database: Overview and Methodological Framework (7 Pp). *Int. J. Life Cycle Assess.* **2005**, *10* (1), 3–9.

(7) Johnson, J.; Jirikowic, J.; Bertram, M.; van Beers, D.; Gordon, R. B.; Henderson, K.; Klee, R. J.; Lanzano, T.; Lifset, R.; Oetjen, L.; Graedel, T. E. Contemporary Anthropogenic Silver Cycle: A Multilevel Analysis. *Environ. Sci. Technol.* **2005**, *39* (12), 4655–4665.

(8) MINSUR S.A. *Memoria Annual 2018*; MINSUR S.A.: Lima, Peru, 2018.

(9) PT Timah. *Annaul Report 2018*; PT Timah: Bangka, Indonesia, 2019.

(10) Evraz plc. *Evraz Annual Report & Accounts 2018*; Evraz plc: London, 2019.

(11) Alliance Mineral Assets Ltd. *Interim Financial Report for the 12 Months Ended 31 December 2018*; Alliance Mineral Assets Ltd.: Osborne Park, Western Australia, Australia, 2019.

(12) Pilbara Minerals Ltd. *2020 Annual Report*; Pilbara Minerals Ltd.: West Pert, Western Australia, Australia, 2020.

(13) AMG Advanced Metallurgical Group N.V. *Investor Presentation February 2019*; AMG Advanced Metallurgical Group N.V.: Amsterdam, Netherlands, 2019.

(14) Jaskula, B. W. Lithium. In *Minerals Yearbook 2017*; U.S. Geological Survey: Reston, 2020; pp 44.1-44.10.

(15) Jiangxi Western Resources Lithium Industry Co., L. *Assessment Report on Mining Rights of Heyuan Lithium (Spodumene) Mine [in Mandarin]*; Jiangxi Western Resources Lithium Industry Co., Ltd.: Ganzhou City, China, 2015.

(16) U.S. Geological Survey. *Mineral Commodity Summaries 2020*; U.S. Geological Survey: Reston, 2020.

(17) Singerling, S. A.; Schulte, R. F. Platinum-Group Metals. In *Minerals Yearbook 2018*; U.S. Geological Survey: Reston, 2021.
